# Interdomain Linker of the Bioelecrocatalyst Cellobiose Dehydrogenase Governs the Electron Transfer

**DOI:** 10.1021/acscatal.3c02116

**Published:** 2023-06-05

**Authors:** Lan Zhang, Christophe V. F. P. Laurent, Lorenz Schwaiger, Lushan Wang, Su Ma, Roland Ludwig

**Affiliations:** † Department of Food Science and Technology, Biocatalysis and Biosensing Laboratory, University of Natural Resources and Life Sciences (BOKU), Vienna, Muthgasse 18, Vienna 1190, Austria; ‡ Institute of Molecular Modeling and Simulation, Department of Material Sciences and Process Engineering, University of Natural Resources and Life Sciences (BOKU), Vienna, Muthgasse 18, Vienna 1190, Austria; § State Key Laboratory of Microbial Technology, Shandong University, Binhai Road 72/N2, Qingdao 266237, China

**Keywords:** bioelectrocatalyst, cellobiose dehydrogenase, direct electron transfer, interdomain electron transfer, linker design, linker evolution

## Abstract

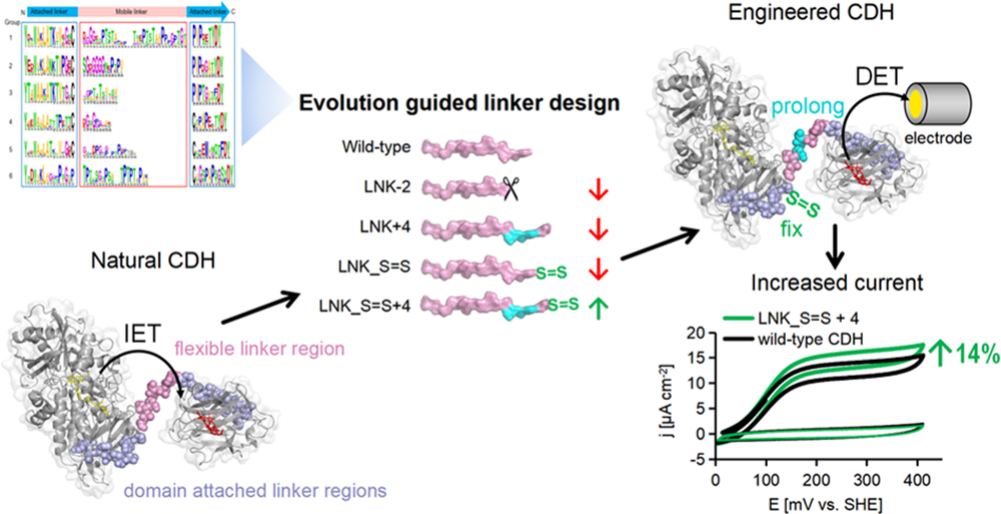

Direct bioelectrocatalysis applied in biosensors, biofuel cells, and bioelectrosynthesis is based on an efficient electron transfer between enzymes and electrodes in the absence of redox mediators. Some oxidoreductases are capable of direct electron transfer (DET), while others achieve the enzyme to electrode electron transfer (ET) by employing an electron-transferring domain. Cellobiose dehydrogenase (CDH) is the most-studied multidomain bioelectrocatalyst and features a catalytic flavodehydrogenase domain and a mobile, electron-transferring cytochrome domain connected by a flexible linker. The ET to the physiological redox partner lytic polysaccharide monooxygenase or, ex vivo, electrodes depends on the flexibility of the electron transferring domain and its connecting linker, but the regulatory mechanism is little understood. Studying the linker sequences of currently characterized CDH classes we observed that the inner, mobile linker sequence is flanked by two outer linker regions that are in close contact with the adjacent domain. A function-based definition of the linker region in CDH is proposed and has been verified by rationally designed variants of *Neurospora crassa* CDH. The effect of linker length and its domain attachment on electron transfer rates has been determined by biochemical and electrochemical methods, while distances between the domains of CDH variants were computed. This study elucidates the regulatory mechanism of the interdomain linker on electron transfer by determining the minimum linker length, observing the effects of elongated linkers, and testing the covalent stabilization of a linker part to the flavodehydrogenase domain. The evolutionary guided, rational design of the interdomain linker provides a strategy to optimize electron transfer rates in multidomain enzymes and maximize their bioelectrocatalytic performance.

## Introduction

Direct bioelectrocatalysis is an emerging field based on interdisciplinary research in electrochemistry, materials science, biochemistry, and molecular biology. Enzymes play a fundamental role in bioelectrocatalytic devices such as biosensors, biofuel cells, biosupercapacitors, and bioelectrosynthetic reactors, enabling efficient catalysis of substrate-specific, regiospecific, and stereospecific redox reactions on electrodes.^1,2^ The applications of this technology range from biomedical analytics to the generation of electricity for low-power electronics and the production of chemicals.^3^ Cellobiose dehydrogenase (CDH, EC 1.1.99.18) is a model enzyme widely used in bioelectrocatalytic devices. For instance, *Phanerochaete sordida* CDH was employed in the design of a direct electron transfer (DET)-based lactose biosensor in 2005, followed by a DET glucose biosensor based on *Corynascus thermophilus* CDH in 2011.^4,5^ Similarly, *Dichomera saubinetii* CDH was used in 2008 to fabricate a noncompartmentalized, mediator-free glucose–oxygen biofuel cell based on adsorbed enzymes exhibiting direct bioelectrocatalysis.^6^ Additionally, the first self-charging chemical supercapacitor based on pseudocapacitance used *C. thermophilus* CDH as an anodic bioelement.^7^ In these examples, the electron transfer rate of CDH was the limiting factor in the generated current, rather than the catalytic rate.

CDH possesses a noncatalytic electron-transferring domain that transfers electrons in vivo to lytic polysaccharide monooxygenase (LPMO).^8^ In bioelectrochemical applications, this cytochrome (CYT) domain is considered a built-in redox mediator that facilitates DET to the electrode.^9^ To optimize DET in this field focuses not only on the development of electrode materials,^10^ nanostructures,^11^ and protein immobilization strategies^12^ but also on protein engineering strategies. One approach involves fusing a CYT domain onto a single-domain enzyme to mimic the function of CDH.^13,14^ Establishing a CYT library expands the potential for utilizing CYT as an electron shuttle for a wider range of biocatalysts.^15,16^ In this and other enzyme engineering approaches, maintaining or improving DET while optimizing the interdomain electron transfer (IET) is crucial.^17,18^ The basis for these strategies lies in the structures of two ascomycete CDHs from *Crassicarpon hotsonii* (syn. *Myriococcum thermophilum*, 4QI6) and *Neurospora crassa* (4QI7), which feature a closed-state and an open-state conformation of the domains, respectively.^19^ The mobility of the CYT domain significantly affects the IET and DET. The switch between the open-state and closed-state conformation of the enzyme depends on the steric and electrostatic interface complementarity as well as the length of the protein linker.^20,21^ In the most productive side-on orientation of CDH relative to a flat surface, e.g., an electrode, the linker plays a key role in IET and DET.^22^ To enhance the electrochemical performance of CDH as a bioelectrocatalyst, linker optimization strategies show promising potential.

Limited research has been conducted on linker engineering in oxidoreductases, particularly with respect to optimizing electron transfer. One study on the interdomain linker region of cellulase 5A demonstrated that a certain degree of linker rigidity and interdomain spacing is critical for proper enzyme function.^23^ In fusion protein design, designed linkers typically consist of a combination of flexible (GGGS) and rigid (EAAAK) linker units.^24^ Early studies on FMN-dependent flavocytochrome *b*_2_ investigated the relationship between linker length and enzymatic function. The variants created with either a shorter linker (C-terminal deletion of 3, 6, or 9 amino acid residues) or an elongated linker (insertion of 3 or 6 amino acid residues near the C-terminal) showed a decreased IET, suggesting that the linker length in the natural enzyme is evolutionarily optimized.^25^ Although several characterized CDHs can achieve DET on various types of electrodes, the linker length and composition are quite different together with reported IET rates ranging from 0.003 to 66 s^–1^.^9^

In this study, we explore from an evolutionary perspective how the interdomain linker differs between CDHs. Although the length and amino acid composition of the linker vary, there are some general rules that allow for their classification. Based on the sequence and structural information of different CDH classes, we have defined linker regions with different functions. To assess their postulated functions and the effect on IET and DET, variants of *N. crassa* CDH IIA (*Nc*CDHIIA; PDB ID: 4QI7) were constructed, produced, and characterized using fast biochemical and electrochemical methods. Steered molecular dynamics (MD) simulations were performed to test the hypothesis and compare the results with experimental data.

## Results

### Linker Sequence Analysis

**Figure 1:**
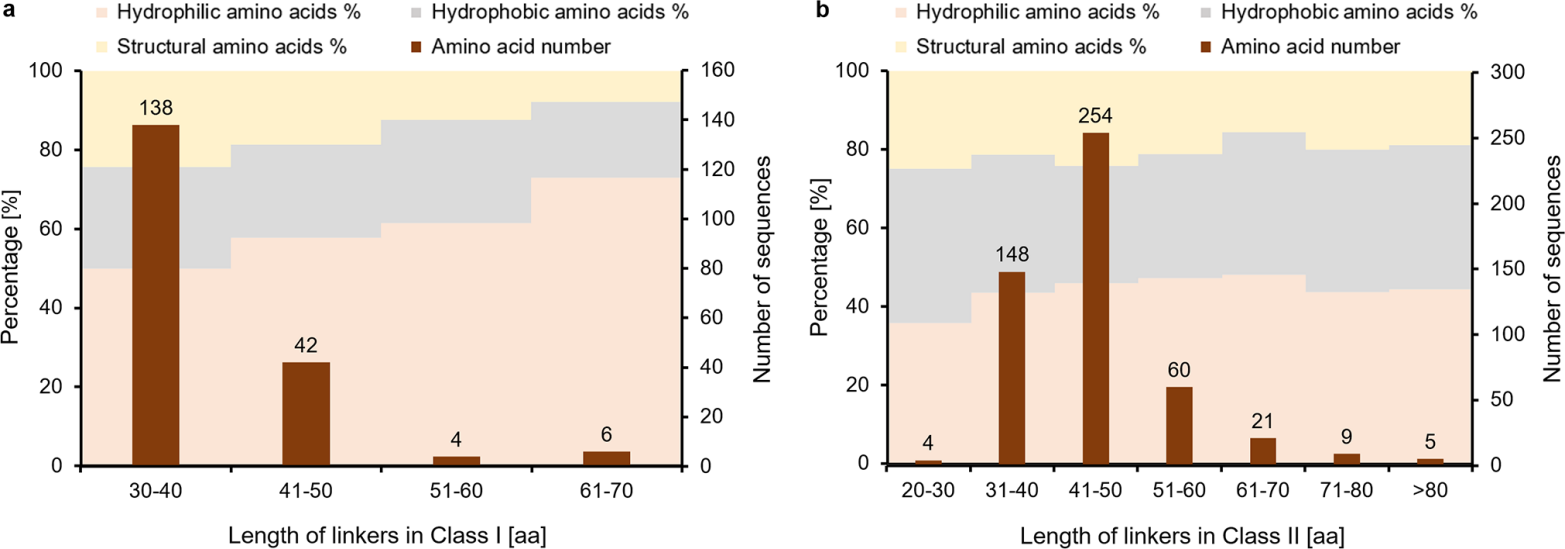
Length and component analysis of linkers. (a) Statistics of basidiomycete Class I CDHs. The histogram shows the distribution of linker length and the background shows the frequency of hydrophilic (Ser, Thr, Glu, Asp, Asn, Gln, His, Lys, Arg), hydrophobic (Ala, Val, Leu, Ile, Tyr, Phe, Trp, Met), and amino acids with a potential structural function (Gly, Pro, Cys). (b) Data for ascomycete Class II CDHs.

CDHs form a diverse enzyme family within the glucose–methanol–choline (GMC)-oxidoreductase superfamily. Based on the latest phylogenetic analysis, CDHs can be divided into four classes.^26^ For this study, we only considered the well-studied basidiomycete Class I CDH sequences and ascomycete Classes II CDH sequences. Together with previously reported crystal structures,^19^ we redefined the boundaries of the CDH linker. A highly conserved Tyr195 initiates the last, C-terminal secondary structure element of the CYT domain, a short α-helix, which in the available crystal structures is in contact with the domain. Since this Tyr195 is conserved in all sequences it is a well-suited position to define the N-terminal start of the linker, contrary to the end of the helix, which is not clearly defined in sequence alignments. The C-terminal end of the linker is defined by the highly conserved _229_Tyr/Phe-Asp-Tyr_231_ motif, which is, according to the crystal structures, also firmly connected with the DH domain. We include these residues in the linker for reasons that will be discussed later. From all available *cdh* sequences, 190 Class I CDH linkers and 501 Class II CDH linkers were extracted (Figure 1). Most of the analyzed Class I linkers have a length between 30 and 40 amino acids, whereas the majority of Class II linkers are longer with a length between 40 and 50 amino acids (Figures S1, S2 and Tables S1, S2). Very few linkers are shorter than 30, or longer than 60 amino acids. The linkers are formed by mostly hydrophilic amino acids, the percentage is higher in Class I linkers and the percentage is increasing with linker length. Hydrophobic amino acids occur more often in Class II linkers. The percentage of amino acids with a potential structural function such as Gly, Pro, Cys is relatively high in linkers of both CDH classes. Some frequently occurring amino acids like Ser, Thr, and Asp as well as Tyr, Trp, and Cys are strongly conserved.

**Figure 2:**
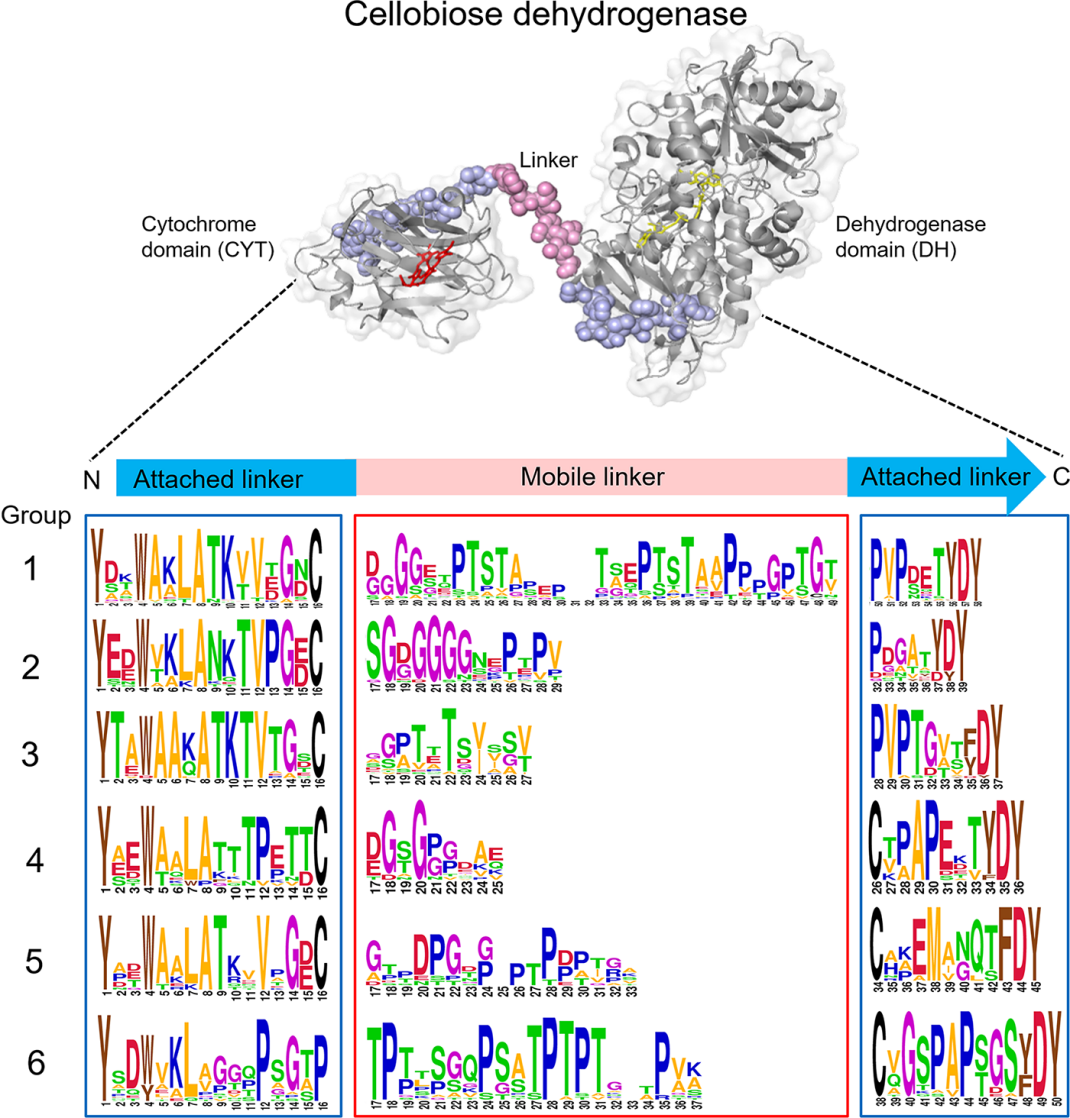
Grouping and definition of Class II CDH linkers by sequence alignment and frequency analysis. One hundred fifty CDHs out of 501 were assigned to six groups. The other sequences show strong similarities to these groups (Figure S8). The N-terminally attached linker (to CYT) is shown in the blue box followed by the mobile linker in the red box and finally the C-terminally attached linker (to DH) in a blue box.

A multiple sequence alignment showed that especially Class I linkers vary greatly in length and sequence composition. Structure similarity network (SSN) analysis was therefore used to discover interrelationships between linkers as a collection of pairwise alignments between sequences^27^ and used to group CDH linker sequences (Figure S3). At an alignment score of 1 × 10^–10^, 150 Class II linker sequences could be assigned into 6 independent groups. A subsequent phylogenetic analysis with those 150 full sequences resulted in similar clades and confirms an evolutionary relationship between the groups (Figure S4). However, the positions of a few CDHs of Group 1 differed between the phylogenetic tree and the SSN network, due to the explicit evolutionary model used for phylogenetic analysis.^28,29^ To visualize the sequence properties of the linker Groups 1–6, sequence logos were generated (Figures 2 and S5). Based on the sequence logos and crystal structures, three linker segments were defined. The middle segment, which showed the highest sequence variation is termed mobile linker and the flanking segments are termed attached linkers. Attached linker segments typically feature a conserved Cys, which can form a disulfide bond to the CYT domain, or a Pro-Val-Pro motif, which interacts with the dehydrogenase (DH) domain. In contrast, the mobile linker typically comprises many hydrophilic amino acids and Gly for increased flexibility. The mobile linker also contains a high percentage of Ser and Thr, which are potential *O*-glycosylation sites.^20^ In addition to the six groups shown, the remaining 351 CDHs of Class II were grouped further by SSN using a lower threshold. The resulting additional eight groups can be assigned to the six groups with minor differences (Figures S6–S8).

Linkers of Groups 1, 2, 3, 4, and partially 5 have a conserved Cys residue at the end of the N-terminal attached linker to bind it covalently to the CYT domain. Similarly, linkers of Groups 4, 5, and 6 (Figure 2) have a Cys residue in the C-terminal attached linker which attaches the C-terminal attached linker segment covalently to the DH domain. This Cys is not found in Groups 1, 2, and 3, but is replaced by a Pro-Val-Pro motif. Interstingly, all CDHs employed in bioelectrocatalytic research and applications are all from the Groups 1, 2, or 3. To explore the function of a covalently attached C-terminal attached linker and the different lengths of a linker, *Nc*CDHIIA (Group 3) variants were designed (Table 1).

**Table 1:**
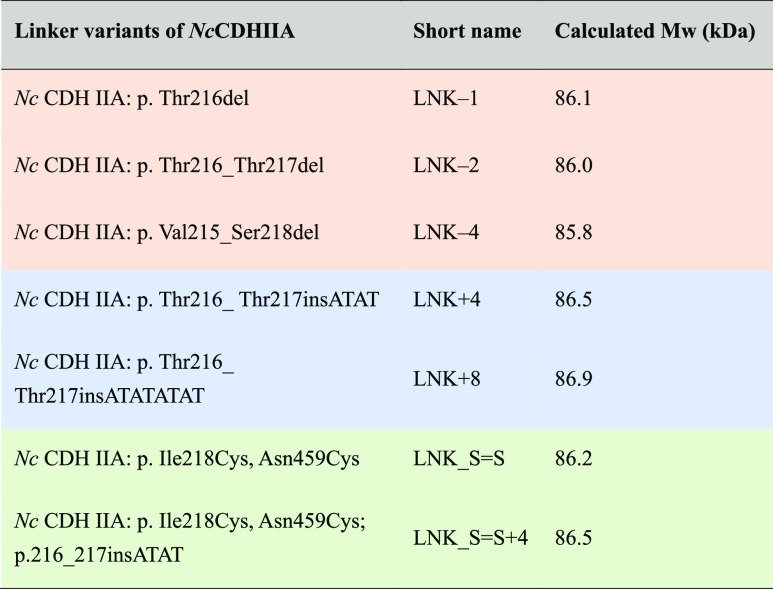
Overview of *Nc*CDHIIA Linker Variants

### Recombinant Protein Production

**Figure 3:**
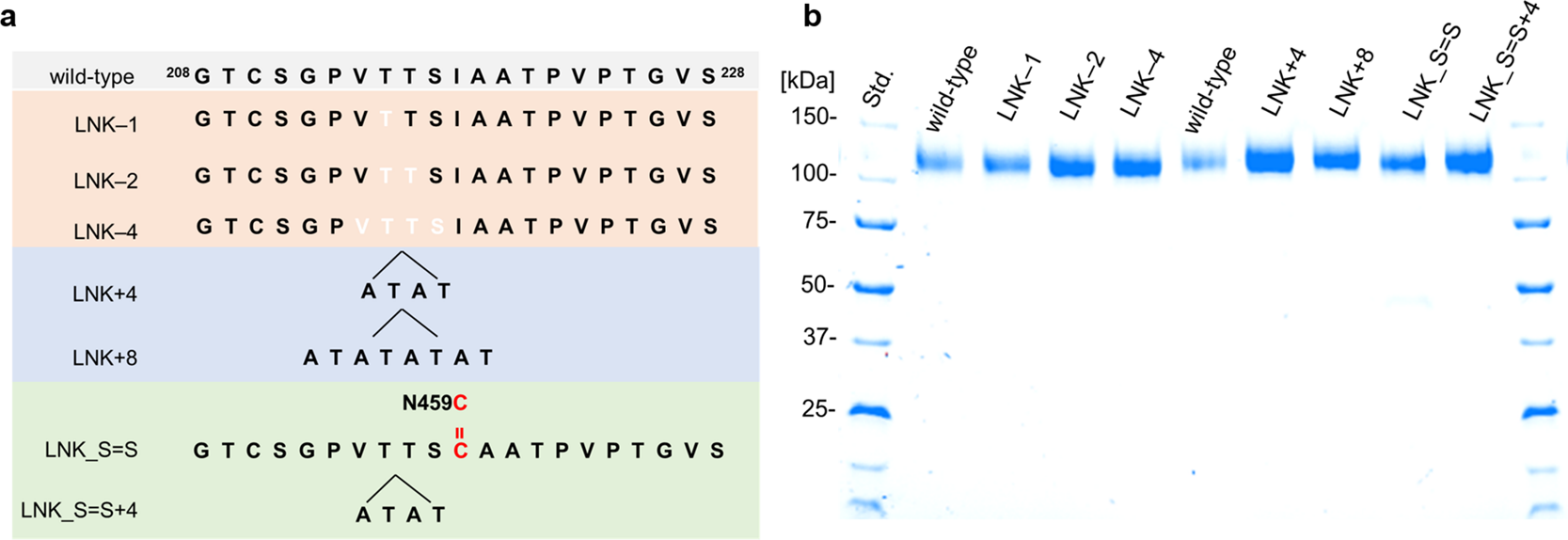
*Nc*CDHIIA variants. (a) Sketch map of variants with different linkers. (b) Purified CDH variants visualized by SDS-PAGE and Coomassie staining.

Seven different *Nc*CDHIIA linker variants were constructed to investigate the influence of the linker on IET and DET (Table 1). The deletion of one, two, and four amino acids shortened the mobile linker in the variants LNK–1, LNK–2, and LNK–4, respectively (Table 1 and Figure 3a). Two elongated linker variants, LNK+4, and LNK+8, were generated by inserting two or four Ala-Thr repeats into the mobile linker, respectively. LNK_S=S attaches the linker to the DH domain by introducing the mutations I218C and N459C in *Nc*CDHIIA. These two amino acids are close together in the crystal structure (PDB ID: 4QI7), which favors the formation of a disulfide bond. An additional insertion of four amino acids (ATAT) in LNK_S=S + 4 increases the length of the mobile linker segment (Table 1 and Figure 3a).

All constructed CDH variants were produced and purified. During the purification procedure, we noticed that variants with a shorter linker bound were weaker to the hydrophobic interaction resin or the ion exchange resin compared to the wild-type CDH and variants with an elongated linker. This complicated the purification and resulted in a lower yield of LNK–1, LNK–2, and LNK–4. The molecular masses of the linker variants were determined by SDS-PAGE (Figure 3b). All variants and the wild-type CDH showed bands around 120 kDa instead of the calculated values (Table 1), which indicates glycosylation (Figure 3b). The formation of the disulfide bond in LNK_S=S was verified by Ellman’s test.

### Steady-State Activity and Electron Transfer

**Figure 4:**
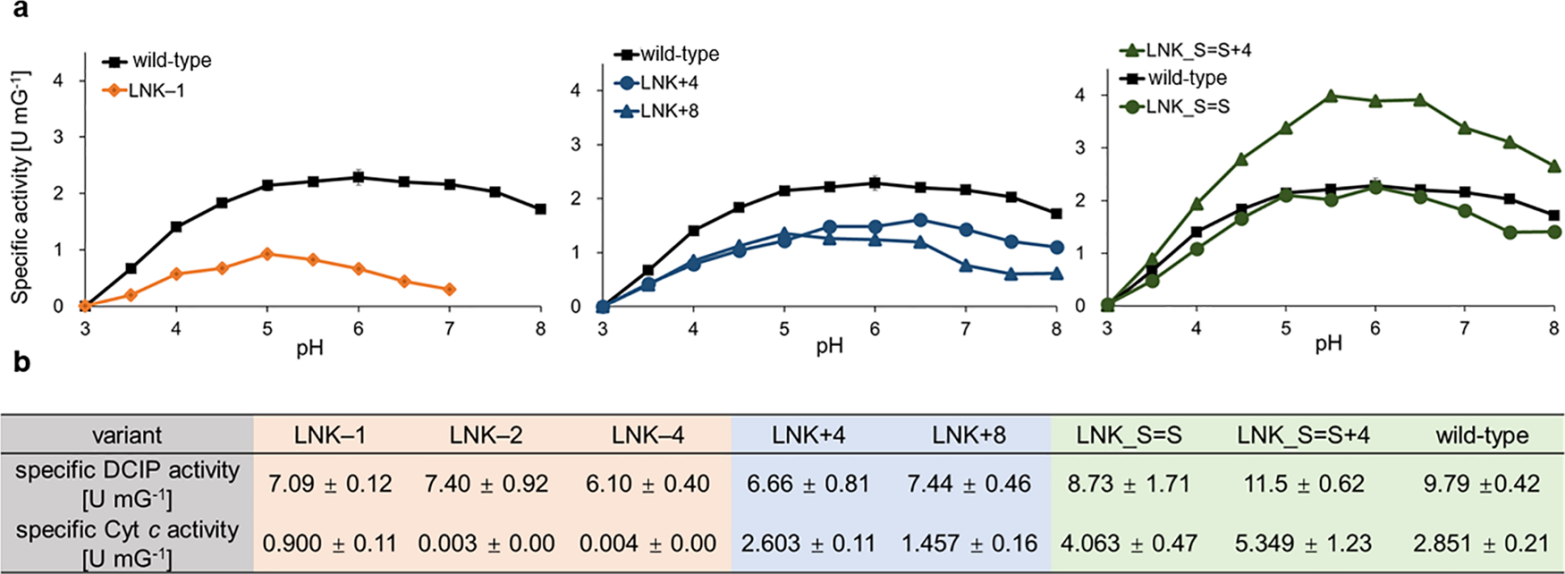
pH-dependent specific activity of *Nc*CDHIIA variants. (a) pH profiles of the wild-type CDH compared with a short linker variant LNK–1, longer linker variants LNK+4 and LNK+8, and disulfide-bridged linker variants LNK_S=S and LNK_S=S+4. The pH-dependent specific activity was measured with the cytochrome *c* assay using McIlvaine buffer 3.0–8.0. (b) Comparison of DCIP and cytochrome *c* assay specific activities in 100 mM potassium phosphate, pH 6.0.

The specific activity for wild-type *Nc*CDHIIA and the seven linker variants was measured using the disaccharide lactose as the substrate and the two-electron acceptor 2,6-dichlorindophenol (DCIP, Figure 4b). The DCIP activity measures the turnover rate of the substrate at the FAD cofactor in the DH domain. The linker variants typically showed an 11–37% decrease in the specific activity compared to wild-type CDH with the exception of LNK_S=S + 4 which showed a 19% increase, which is minor and not correlated to enzyme purity as later shown by spectral analysis. The slightly varying specific activity of the DH domain is very likely the result of a differing FAD cofactor occupation that originates from slightly varying fermentation conditions and timelines.^30^

In contrast to the DCIP activity, the specific activity with cytochrome *c* depends on the CYT domain as an electron shuttle between the DH domain and the terminal electron acceptor, in this case, cytochrome *c*. It therefore involves the IET as well as the direct electron transfer interaction between the cytochromes (Figure 4b). To investigate the influence of the linker length on the IET, a pH profile for wild-type *Nc*CDHIIA and all its linker variants was measured (Figure 4a). *Nc*CDHIIA shows a plateau of activity between pH 5.0 and 6.5 with a pH optimum of 6.0. The pH optima of the variants are between pH 5.0 and 6.5. All variants except LNK_S=S and LNK_S=S+4 with the covalently attached linker showed lower specific activity than the wild-type. In the case of LNK-1, the shortened linker already slightly restricts the mobility of the CYT domain to form a perfect closed state or increases the time to do so. The specific activity of LNK-1 with cytochrome *c* at pH 5.0 is only 32% of the wild-type, for LNK-2 and LNK-4 no activity is detectable and the ability to form the closed state and perform IET is lost. The variants LNK+4 and LNK+8 feature a longer linker, which gives the CYT domain more freedom to move, but also generates a bigger distance between both domains in the open state, which results in a lower IET and subsequently lower cytochrome *c* activity.

Besides a reduced cytochrome *c* activity, LNK–1 also showed an acidically shifted pH optimum of 5.0. The deletion of hydrophilic and uncharged Thr215 in the linker as well as the linker composition indicate that it is not the linker that changes the pH optimum, but its effect on the orientation of the CYT domain in the closed state. LNK-1 also shows a faster drop in cytochrome *c* activity above its pH optimum than the wild-type—a 68% reduction at pH 7.0 compared to the pH optimum. The same reduction of the IET is observed for LNK+8, which drops faster at neutral and alkaline conditions than LNK+4. Here, the combination of the longer linker with the electrostatic repulsion of both domains results in an on average larger distance between both domains and a less frequent formation of the IET-competent closed state.

The introduction of a disulfide bond between the DH domain and the linker in LNK_S=S did not alter the specific activity or the pH optimum, which indicates that a 10 amino-acid-long part of the linker between Cys218 and the N-terminus of the DH domain is originally attached to the DH domain. The prolongation of the linker before Cys218 by an ATAT motif (LNK_S=S + 4) gives CYT a higher mobility range which, interestingly, increases the cytochrome *c* activity almost 2-fold over the wild-type CDH.

### Presteady-State Rates of FAD and CYT Reduction

UV–Vis spectra of *Nc*CDHIIA and its linker variants show the same spectra in the oxidized state (Figure S9). Although the spectra were originally measured to show the purity of the prepared enzymes, the spectra of the reduced state that were measured 30 s after the addition of cellobiose also indicate the changes in the IET. For the wild-type CDH and most variants, the typical Soret band shift was observed from 420 to 430 nm, while the heme β- and α-peaks appeared at 533 and 563 nm, respectively. However, for LNK–2 and LNK–4, no CYT reduction was observed indicating the loss of IET, which was also observed in the measurements with the cytochrome *c* assay (Figure 4b). Based on the spectral observations, we measured the presteady-state reduction rates of the FAD cofactor in wild-type CDH and variants, which are relatively similar (Table 2), even more so than the steady-state rates obtained with DCIP. This supports the hypothesis of a slightly different FAD occupation in CDH and its variants. The observed FAD reduction rates show no effect of the linker on cellobiose oxidation in the DH domain. They are also at least twofold higher than the subsequent IET measured as the presteady-state reduction rate of the heme *b* cofactor. Here, we observe a big influence of the mutated linkers on the IET. Variants with shortened linkers show a drastically reduced heme reduction rate (LNK–1) or no heme reduction at all (LNK–2, LNK–4) compared to the wild-type CDH and variants with an elongated linker. LNK + 4, LNK_S=S and LNK_S=S + 4 showed a similar or slightly higher heme reduction rate than wild-type CDH. The about three times reduced heme reduction rate of LNK+8 demonstrates that a too-long linker allows a wide separation of the domains and slows down the IET. A moderate elongation by only four amino acids seems not to interfere with the frequent formation of the IET-competent closed state of CDH. This slight elongation of the linker might, however, add some flexibility to the CYT domain to interact with an external redox partner and increase the interprotein electron transfer to LPMO or the DET to an electrode.

**Table 2:**
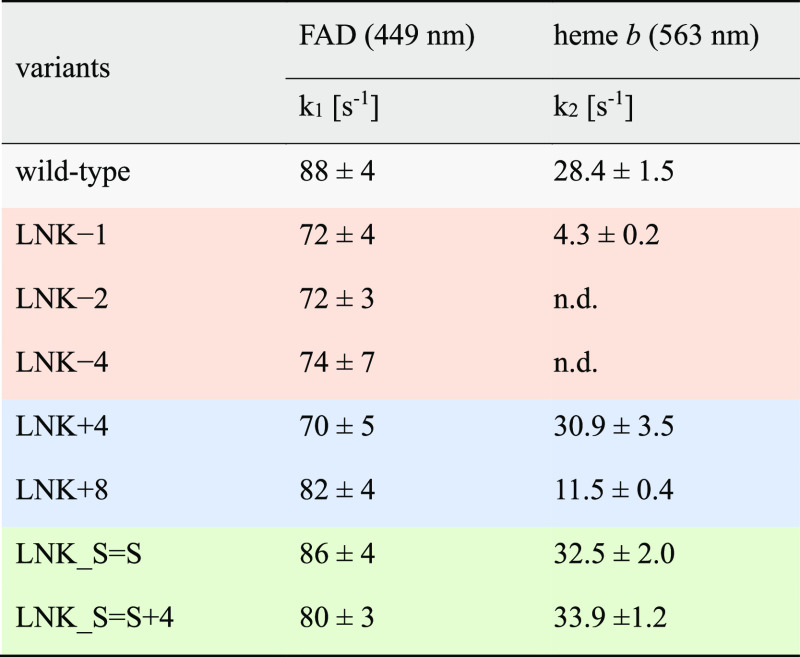
PreSteady-State Reduction Rates of FAD and Heme *b* at pH 5.5 Using 100 mM Cellobiose as Substrate^a^

a n.d., not detected.

### Computational Analysis of CDH Linker Variants

To investigate the molecular reasons for the effect of the linker length on the IET and the pH profile of the IET, we investigated the interface of various *cdh* sequences. The analysis of amino acids at the interface area showed that the interface residues are not conserved between different CDHs (Figure S10). The transient interaction between the two domains is dominated by weak hydrophobic interactions. The precise fit of the two domains mainly depends on spatial complementarity. The mostly weak interactions between the domains ensure the freedom of movement of the CYT domain between the closed and open states. Modeled structures of wild-type *Nc*CDHIIA, LNK–1, LNK–2, and LNK–4 using the closed-state crystal structure of CDH (PDB ID 4QI6) as a template showed that the linker region was more stretched with reduced linker length (Figure S11). For LNK–4, no modeled structure of the closed state could be obtained, indicating that the *Nc*CDHIIA linker is already at a minimum length and any reduction distorts the closed state which reduces the IET (and therefore the cytochrome *c* activity) by changing the interface region and involving new amino acids into the interface. This also alters the electrostatic interactions between the domains that are susceptible to pH.

Steered MD simulations were performed to explore the maximum distance that the linker of the different variants can stretch. In these simulations, the CYT domain was constantly pulled away from the DH domain. Despite the low pulling force, a distortion of the CYT domain could be observed after some time. This distortion was monitored via the distance between the CYT center of geometry (COG) and the Cα of Cys211. The MD simulations were cut off after the distortion measurement within the CYT domain reached 11 Å, a value that was considered appropriate after a visual inspection of the individual MD simulations (Table S3). The largest distances between the COG of the DH and CYT domains were observed for the two variants with an extended linker: LNK+4 and LNK+8. The increased movement radius of the CYT domain reduces the frequency of forming the electron transfer competent closed state. As expected, the COG distance between the DH and CYT domains gradually decreased for variants with a shortened linker. For reference, the COG distance between the DH and CYT domains was 35.6 Å when the two domains were in the closed-state conformation (modeled after PDB ID: 4QI6). The solvent-accessible surface area (SASA) of the linker corresponds with the linker elongation (Figure S12).

**Figure 5:**
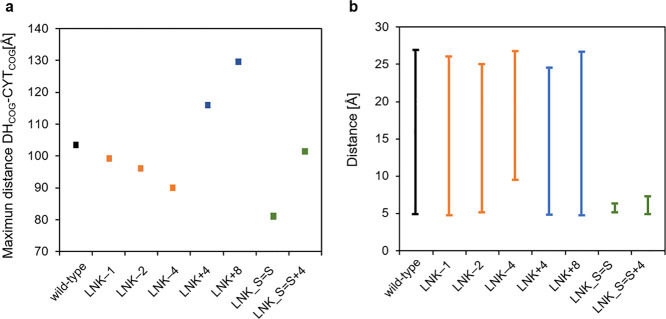
Maximum distance in *Nc*CDHIIA linker variants between the CYT and DH domains. (a) Maximum interdomain COG distance that can be reached by each variant before domain distortion. (b) Range of distances between the linker and the DH domain measured between I218 and N459 (wild-type numbering) during MD simulations.

The computed COG distance of LNK_S=S was over 20 Å smaller than for the wild-type model (Figure 5a). Since there are no differences in the IET rates of the wild-type CDH and LNK_S=S, this means that the attached linker is attached in vivo, but can be detached by the computational procedure. The strategy to stabilize the attached linker at the DH domain by a disulfide bond between I218C and N459C in LNK_S=S was investigated by tracking the distance between these residues was tracked during the MD simulations of all the variants and the wild-type model (Figure 5b). The distances between the Cα of the residues I218 and N459 (wild-type *Nc*CDHIIA numbering) remained relatively confined for the LNK_S=S (Δ 1.2 Å) and LNK_S=S + 4 (Δ 2.3 Å), whereas the wild-type CDH and the other variants shown no stabilization and a detachment of the attached linker from the DH domain (average Δ = 20.4 ± 1.8 Å). This highlights the stabilizing effect of the disulfide bond on the attached linker and domain interaction. Elongating the LNK_S=S linker by 4 amino acids could bring the COG distance of LNK_S=S + 4 back to that of the wild-type CDH, which should result in an increased CYT mobility that could be beneficial for the interaction with macromolecular electron acceptors such as cytochrome *c*, lytic polysaccharide monooxygenase, and electrodes.

### Electron Transfer to the Terminal Electron Acceptor

The last electron transfer step of CDH starts from the reduced CYT domain and delivers a single electron either to the nonnatural electron acceptor cytochrome *c*, or its physiological redox partner LPMO. The interprotein electron transfer (IPET) step from CYT to the type-2 copper center of LPMO was determined by measuring the reoxidation rate of heme *b* in the CYT domain (*k*_2_^563^) in the presence of *Nc*LPMO9C. We found little difference between the wild-type CDH (15.6 ± 0.7 s^–1^), LNK + 4 (13.6 ± 2.4 s^–1^), and LNK_S=S + 4 (18.4 ± 0.4 s^–1^), which indicates that a moderate elongation of the linker does not restrict the CYT domain movement between the open and closed state or improves the interaction with the terminal electron acceptor. However, in another study, a much longer linker reduced the IET in CDH greatly.^21^

However, the increased IPET rate (34.3 ± 2.4 s^–1^) of LNK–1 together with the decreased IET rate reveal a significant change in the transition between the open and closed states. LNK-1 is much more likely to maintain the open state with an exposing heme *b* cofactor that increases the interaction with *Nc*LPMO9C but reduces IET.

### Electron Transfer on Electrodes

Besides LPMO, another important terminal electron acceptor for CDH is an electrode. Here, the reduced CYT domain delivers a single electron directly to the electrode, which is described as direct electron transfer (DET). In this context, DET does not indicate a direct electron transfer from the FAD to the electrode. To investigate the DET of the CYT domain of wild-type CDH and the linker variants, cyclic voltammetry with a 1-thiogycerol-modified gold electrode was performed (Figures 5 and S13). The plot of peak current vs the square root of the scan speed showed a linear trend for all CDHs, which indicates that the system is in a freely diffusing mode (Figure S14).^31,32^ The anodic and cathodic peak currents increased gradually with an increased scan rate (2–500 mV s^–1^) (Figure S15). After determining the optimal scan speed, the midpoint redox potential of the heme *b* was measured at pH 5.5 with a scan rate of 25 mV s^–1^. No significant deviations between the midpoint redox potential of the wild-type enzyme (111 mV vs SHE) and the linker variants (106–116 mV vs SHE) were found.

**Figure 6:**
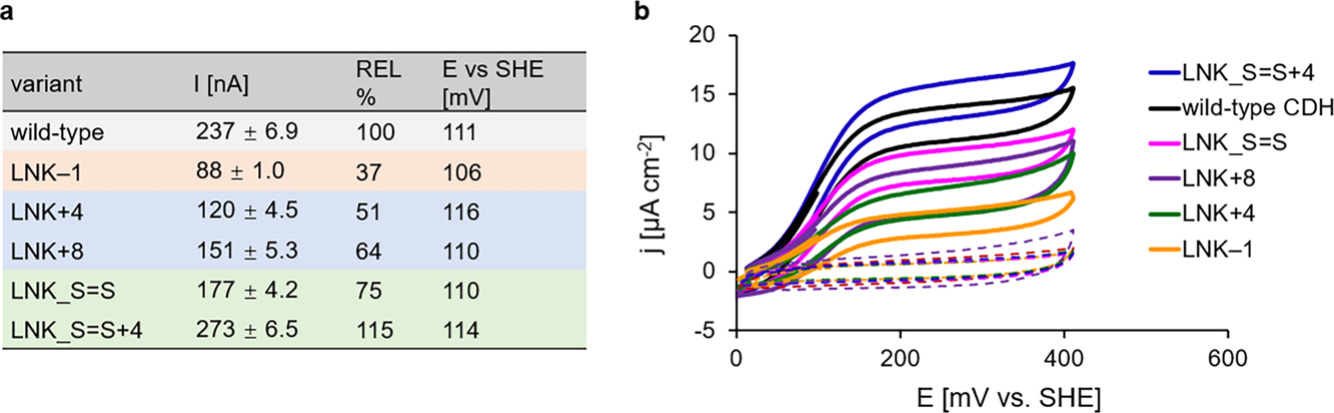
Catalytic currents of *Nc*CDHIIA variants on 1-thioglycerol-modified gold electrodes. (a) Catalytic currents and midpoint potentials of linker variants. (b) Cyclic voltammograms of linker variants at pH 5.5, scan rate = 25 mV s^–1^. The catalytic CVs are indicated by a full line, blanks without substrate are dotted lines in the same color.

The observed current densities (measured at 250 mV vs SHE) are a result of the combination of differing IET and DET rates (Figure 6b). The highest current density was observed for LNK_S=S + 4 (15.9 μA cm^–2^), which is 14% higher than the wild-type CDH (13.9 μA cm^–2^) and supports the data from steady-state and presteady-state experiments that indicate an increased CYT domain flexibility after stabilization of the attached linker. The reduced CYT domain mobility expected in LNK_S=S is also obvious from the lower current density of 10.2 μA cm^–2^. Elongated linkers do neither increase IET nor DET LNK + 8 (8.8 μA cm^–2^) and LNK + 4 (7.1 μA cm^–2^), which a reduced linker length introduces a greatly reduced IET which is demonstrated by LNK–1 (5.1 μA cm^–2^).

## Discussion

Multiple sequence analysis revealed notable features of CDH linkers, where were not apparent from prior studies. The definition of the flanking linker regions that are most often attached to the CYT or DH domain and a mobile central linker region allows a better understanding of the linker function based on its amino acid composition. The mobile linker region of Class I CDHs shows a consistent composition rich in Gly, Ser, and Thr. The high-sequence similarity was reflected in being below the minimum grouping threshold for SSN analysis.^40^ More variations appear in Class II CDH linker sequences that were grouped into six groups. Here, highly conserved sites were found. In almost all linker N-termini a Tyr defines the start of the linker, which forms an α-helix with other four or five amino acids. This structure is common and conserved in all analyzed CDH structures.^16^ The next highly conserved position is a Cys residue in the N-terminal attached linker, that forms a disulfide bridge with the CYT domain, which has been confirmed by previous LC–MS/MS analyses (Cys167-Cys211 in *Crassicarpon hotsonii* CDH).^20^ After this Cys, the mobile linker region starts. The length of the mobile part is between 15 and 30 amino acids in Class II linkers. The abundance of Gly and Ser residues gives the linker a high flexibility, which also leads to the difficulties of full-length enzyme crystallization.^19^ Other studies reported *O*-glycosylation on Ser and Thr residues in the mobile linker of *C. hotsonii* CDH.^19,20^*O*-glycans are also often found in the hinge regions of matrix metalloproteinases and lgA antibodies.^33^ The abundant Ser and Thr residues in the Class II linker groups 1, 3, and 6 provide many potential *O*-glycosylation sites.^20^ The highly conserved Pro-Val-Pro motif or a conserved Cys residue at the corresponding position is the start of the next attached linker segment. Its C-terminal endpoint is given by the highly conserved Tyr/Phe-Asp-Tyr motif. A look at other GMC-oxidoreductases, such as flavocytochrome *b*_2_ lactate dehydrogenase (PDB ID: 1FCB), flavohemoglobin (PDB ID: 4G1V), flavocytochrome *p*-cresol methylhydroxylase (PDB ID: 1DII), flavocytochrome *c* sulfide dehydrogenase (PDB ID: 1FCD), and flavocytochrome *c*_3_ (PDB ID: 1QJD), which shows no sign of such motifs and reflects the evolutionary uniqueness of the linker in CDH. Most characterized ascomycetous Class II CDHs from *Chaetomium atrobrunneum*, *Hypoxylon hemeatostroma*, *N. crassa*, and *Stachybotrys bisbyi* have linkers that belong to linker Group 1, 2, or 3. The linker of *C. thermophilus* CDH is a representative of Group 4, and the CDH linker from *D. saubinetii* is a typical Group 6 type linker. For those characterized CDHs, which show DET on electrodes, the length of mobile linker varies from 10 to 22 amino acids, and the pH-dependent activity varies from acidic to alkaline.^34^

The reduced cytochrome *c* activity and the pH shift as well as the reduced IET rates directly obtained from stopped-flow measurements result from the shortened linker variant LNK–1, which hinders the two domains to form the optimal closed-state. The pH shift originates from the sterically distorted interface due to the shorter linker and a mismatch of electrostatic interactions. The interaction of attractive and repulsive electrostatic charges at the interface is crucial for the formation of the transient CYT/DH complex, but also for the following fast dissociation of both domains. A shortening of the linker leads to the different positioning of the CYT domain at the DH interface and can shift the pH profile to a lower pH when more repulsive electrostatic interactions due to the pairing of negatively charged Asp side chains are formed. The interface conservation analysis showed that the interface area is not conserved in CDHs, which requires the evaluation of each individual CDH interface. The amino acid pairs of *Nc*CDHIIA at the interface was positioned and mostly weak interactions ensures the freedom of conformational switch. Modeled structures of wild-type *Nc*CDHIIA, LNK–1, LNK–2, and LNK–4 showed that the linker region was stretched obviously with reducing of linker length. Especially, for LNK–4, the modeled structure cannot be successfully built using a closed-state crystal structure, resulting in an open-state structure. These results revealed that the natural linker is already optimized for the shortest length.

Similar results in flavocytochrome *b*_2_ have been reported, the changes in the length of the interdomain hinge region result in severe disruption of heme reduction.^25^ A study on the linker length of GH5-CBM3 cellulases from *Paenibacillus genus* showed variant with a linker two times longer than that of the wide-type protein attained the highest catalytic performance, and other variants with excessively long or short linkers were equally detrimental to catalysis.^23^ All these studies show that the optimization of the spatial distance of multidomain enzymes is feasible and worthwhile.

Steered MD simulations suggest that the weaker nonbonded interactions between the C-terminal attached linker and the DH allow the attached linker segment to dissociate from the DH. This dissociation results in a two-step length change of the interdomain linker during the open/closed conformational switch, which could be important for the IPET to its natural redox partner LPMO. To test this hypothesis, the linker of *Nc*CDHIIA was changed from a one Cys linker type to a double Cys linker type, thereby attaching the linker covalently to a corresponding cysteine on the DH domain to prevent linker dissociation. However, presteady-state experiments with LPMO showed no significantly different IPET rates for the LNK_S=S + 4 with the attached linker or LNK_+4 with a slightly elongated attached linker. This shows that the CYT domain does not need to move a great distance to make efficient contact with a molecule like LPMO. In contrast, the slight shortening of the linker in LNK–1 had a positive effect and increased the IPET more than 2-fold. The modeled structures of shortened linker variants show that the linker region was stretched with reduced linker length; therefore, open conformation is prior.

The effect of the different linkers changes when applied to an electrode. Here, preventing the dissociation of the linker in the LNK_S=S variant, resulted in an enhanced IET but still reduced DET rate on the electrode. The variants of LNK_S=S+4, LNK+4, and LNK+8 have an insert Ala-Thr-Ala-Thr in the mobile liker (between Thr216_Thr217). This insert was designed to increase the length of the linker which could form a coil without a special function. Ala-Thr-Ala-Thr also exist in the mobile linker of *D. saubinetii* CDH, which has a quite good electron transfer efficiency.^6^ This insertion promoted DET of LNK_S=S+4, which may be because the longer mobile linker increases the possibility of open conformation compared with LNK_S=S. For LNK+4 and LNK+8, the longer mobile linker has a better IET but reduced DET, which confirmed that the mobility of linker and the frequency of the switch between open and closed state is the rate-limiting steps. These results are consistent with previous studies showing that IET is dependent on the mobility of the CYT domain, and the mobility of the CYT domain is affected by the orientation of the DH domain and the length of the interdomain linker.^21,22^ Another study investigated chimeric CDH variants and found that the occurrence of IET highly depends on a close distance between the FAD and heme *b* cofactors, respectively, the DH and CYT domains, the mobility of the linker, and the correct orientation of the CYT domain in respect to the DH domain.^24^ Steric mismatches between the domain surfaces or repulsive electrostatic interactions at the interface hinder a successful IET.

The CDH linker variants showed different trends in kinetic studies and electrochemical measurements. For example, compared with the wild-type enzyme, LNK_S=S had a higher IET but lower DET. This revealed the electron transfer between the enzyme and electrode was limited, most likely due to the reduced frequency of open-close conformation switch by the extra disulfide bond. In this study, proteins diffused freely on the electrode surface with an unordered orientation, so it is not optimal to reach the best DET orientation.^22^ A previous study has shown that the mobility of CYT domain determines the IET from FAD to heme *b* and the DET from heme *b* to the electrode surface.^21,22^ The bioelectrocatalytically best-performing variant LNK_S=S+4 showed a combination of optimized IET and DET. The rational design from an evolutionary point of view is a promising strategy for optimizing direct electron transfer rates of multidomain enzymes, as well as for other fusion proteins that might be of interest in bioelectrocatalytis applications.

## Methods

### Linker Sequences Analysis

All available *cdh* sequences (i.e., characterized, hypothetical, putative, and unnamed *cdh* sequences and enzyme sequences associated with the glucose–methanol–choline (GMC) oxidoreductase family) were collected from the NCBI and UniprotKB databases (accessed in 2021). The linker part is extracted according to the new definition, and sequence similarity networks (SSNs) were used to group all potential *cdh* linker sequences and only Class I and II enzyme sequences with characterized examples were selected at an alignment score of 10^–10^ in this study.^26^ The multiple sequence alignment was performed by MUSCLE for both classes. Sequence LOGOs were made by WebLogo (https://weblogo.berkeley.edu/logo.cgi). In order to analyze the linker diversity, repeated sequences were deleted. Different amino acid compositions and linker lengths were counted, hydrophilic amino acids including serine, threonine, glutamic acid, aspartic acid, asparagine, glutamine, histidine, lysine, and arginine. Leucine, isoleucine, valine, alanine, tyrosine, phenylalanine, and tryptophan were considered hydrophobic amino acids. Special cases were glycine, proline, and cysteine.

### Molecular Evolutionary Genetics Analysis

The full sequences of Class I and Class II were imported into MEGA (version 11) respectively, and aligned with MUSCLE. Evolutionary relationships were inferred with the maximum likelihood method (JTT model). The bootstrap consensus tree is based on 1000 replicates. Different clades were defined depending on linker sequence types.

### Domain Interface Analysis

The interacting amino acids in domain interfaces of *Nc*CDHIIA were determined one by one in PyMOL software according to different bond screening principles for hydrophobic force, hydrogen bonding, and electrostatic interaction. The Consurf software was used to analyze the overall sequence conservation (https://consurf.tau.ac.il/). The multiple sequence alignment of all CDH sequences from Class I and Class II CDHs was performed by MEGA 4.0, and binding sequence spectra were drawn using the sequence logo.

### Chemicals and Media Components

All chemicals and media components were purchased in the highest purity from Sigma Aldrich (Stein Heim, Germany), Fluka (Buchs, Switzerland), Roth (Graz, Austria) and VWR (Darmstadt, Germany) or Bio-Rad (Hercules, USA). Low-salt Luria Bertani (LS-LB) broth (10 g L^–1^ peptone from casein, 5 g L^–1^ yeast extract, 5 g L^–1^ NaCl, and in the LS-LB selection plates has additional 15 g L^–1^ agar and 25 mg L^–1^ zeocin) was prepared for *Escherichia coli*. The bacteria were cultivated at 37 °C. The KM71H (Mut^S^) yeast strains were cultivated at 30 °C in YPD medium (containing 20 g L^–1^ peptone from casein, 10 g L^–1^ yeast extract, 4 g L^–1^d-glucose and 100 mg L^–1^ zeocin, in the case of YPD plates additionally 15 g L^–1^ agar agar). Media components were dissolved in highly pure water and autoclaved for 20 min at 121 °C and 1 bar.

### Organisms

NEB DH5-alpha competent *E. coli* strain from New England Biolabs (Frankfurt, Germany) was used for linker variants cloning. The *Pichia pastoris* strain KM71H (Mut^S^), supplied by Invitrogen (Carlsbad, USA), was used for the expression of all the linker variants. The vector of wild-type *p*PIC*_Nc*CDHIIA was used as a template to design and clone all linker variants on the linker region. Primers were synthesized by Microsynth (Vienna, Austria), and all PCR primers used in the construction of plasmids for linker variants of *Nc*CDHIIA protein overexpression are listed in Table S4. The correct sequences of plasmids were confirmed via DNA sequencing by Microsynth (Vienna, Austria).

### Expression, Isolation, and Purification

The encoding cDNA for CDH linker variants was ligated with the expression vector pPICZαA and transformed into electrocompetent *P. pastoris* KM71H cells by electroporation. Then, several single colonies were selected on zeocin antibiotic (100 mg L^–1^)-containing YPD agar plates for 96-deep-well screening. The colony with the highest activity of variants was used for fermentation. The fermentation was performed in shaking flasks according to the manual of the *Pichia* fermentation process guidelines (Invitrogen, Manual part no. 25-0172). The buffered glycerol-complex medium (BMGY) and buffered methanol-complex medium (BMMY) were used for preculture and acculturation, respectively. The yeast cultures were harvested after 120 h of cultivation. *Nc*CDH linker variants were purified from the supernatant by a two-step chromatographic procedure The supernatant was stabilized with 30% (v/v) ammonium sulfate before the first purification step, which was performed using a Butyl Sepharose 4FF column (all chromatographic equipment and materials from GE Healthcare Biosciences). The column was equilibrated with a 50 mM sodium acetate buffer pH 5.5 containing 30% (satd.) ammonium sulfate. The variants were eluted by a linear gradient from 30 to 0% ammonium sulfate in 5 column volumes at a flow rate of 10 mL min^–1^ and collected in 5 mL fractions. The active fractions were determined with activity assays (described here below) and a buffer exchange to 10 mM HEPES pH 7.5 was performed with a Sephadex G-25 desalting column after concentrating the samples. The conductivity was below 1.5 mS cm^–1^. The enzyme solution was applied to a Source 30 Q resin (52 mL) equilibrated with 10 mM HEPES buffer, pH 7.5. the enzymes were eluted by a linear gradient in 10 column volumes from 0 to 1 M NaCl at a flow rate of 5 mL min^–1^. The proteins were collected in 3 mL fractions and pooled according to activity and SDS-PAGE. The pooled fractions were concentrated and a buffer exchange to 50 mM acetate acid pH 5.5 was performed with Amicon Ultra centrifugal filters cutoff of 10 kDa. The SDS-PAGE analysis of all preparations in this study is displayed in Figure 3. Theoretical molecular weight was calculated with an online tool Expasy-Protparam.

### Enzyme Activity Assays and Protein Quantitation

The activity of the CDH variants was determined by the concomitant reduction of 0.03 mM DCIP (2,6-dichlorindophenol, ε_520_ = 6.9 mM^–1^ cm^–1^) or 50 μM cytochrome *c* from equine heart (ε_550_ = 19.6 mM^–1^ cm^–1^). Assay reactions were performed in 100 mM potassium phosphate, pH 6.0. The activity was measured with 30 mM lactose as the saturating substrate. Assay reactions were monitored for 180 s at 30 °C at 520 nm in a LAMBDA 35 UV–vis spectrophotometer equipped with a temperature-controlled 8-cell changer (PerkinElmer). To cover the wide pH range from 3.0 to 8.0 for activity profiles, the McIlvaine buffer system was used. The protein concentration of wild-type and linker variants was determined with the absorbance at 280 nm. One unit of enzyme activity was defined as the amount of enzyme necessary to reduce 1 μmol lactose per min under the assay conditions. The presence of free thiols in proteins was quantified through the use of the chromogenic reagent 5,5′-dithiobis-(2-nitrobenzoic) acid (DTNB), 100 μM DTNB and pure proteins (more than 10 μM in the reaction cuvette) in 100 mM Tris buffer pH 8.0, the optical absorbance were measured at 412 nm after 5 min incubation.^35^

### Transient-State IET and Interprotein Electron Transfer (IPET) Rates

Fast kinetic of wild-type CDH and linker variants were determined with a stopped-flow spectrometer (SX20, Applied Photophysics Ltd., UK). Measurements were performed with 5 μM protein and 100 mM cellobiose as substrate. Absorbance changes were recorded from 0 to 30 s. The redox state of the FAD cofactor was monitored at 449 nm, while the heme *b* cofactor was monitored at 563 nm. The reduction of NcLPMO9C by CDHs was performed with the same stopped-flow spectrometer in the sequential mixing mode. First, 2 μM CDH was fully reduced by the cellobiose (2.2 μM) in the aging loop for 30 s until the second electron on the FAD used up according to the literature.^21^ When the heme b in the CYT domain was oxidized to about 70 to 80%, we combine it with *Nc*LPMO9C (20 μM) to measure the reoxidation of heme *b*. The trace of the absorbance at 563 nm was fit to a single exponential curve. All experiments were performed in 50 mM sodium acetate buffer pH 5.5 at 30 °C.

### Voltammetric Measurements

The cyclic voltammetry and chronoamperometry were performed with an Autolab PGSTA204 potentiostat (Metrohm) using thiol-glycerol modified gold electrodes (1.6 mm diameter, BASi, USA). A SAM was assembled by immersing the cleaned gold electrode into a 10 mM 1-thioglycerol solution for at least 1 h at room temperature. Voltametric measurements were performed in McIlvaine buffer at pH 5.5 containing 100 mM KCl as a supporting electrolyte. All solutions had been degassed sufficiently. To maintain an oxygen-free atmosphere throughout the procedure, nitrogen was circulated into the solutions. 20 μL of the enzyme was placed in a Teflon holder on top of the electrode as a container for the protein at a concentration of 80 μM. A filter membrane with a cutoff of 3.5 kDa keeps the fluid confined. It is possible to restrict the enzyme in the membrane, enabling low molecular weight substrates and products to pass through.

### In Silico Experiments

Homology models for the LNK+4, LNK+8, LNK–1 LNK–2, and LNK–4 variants were generated using the SWISS-MODEL online tool and the SWISS-MODEL web interface (ProMod3 3.2.1) with the close-state CDH crystal structure (PDB ID: 4QI6) or the *Nc*CDHIIA crystal structure (PDB ID: 4QI7) as templates.^36^ The residues I218, N459 in the wild-type crystal structure, and I223, N464 in the LNK+4 homology model were substituted with cysteines using the “Mutagenesis” tool in open-source PyMOL (version 2.5.0a0, Schrödinger, LLC) to generate the LNK_S=S and LNK_S=S + 4 variants, respectively. The cysteine side chain rotamers were chosen such that the sulfur atoms were as close as possible.

MD simulations settings. MD simulations were performed with the GROMACS 2021.4 biomolecular simulation package.^37^ First, the models were prepared in open-source PyMOL (version 2.5.0a0, Schrödinger, LLC). The preparation included the removal of all solvent molecules, glycans, and cofactors. Also, the models were aligned such that the center of mass of the DH domain is at the (*x*, *y*, *z*) coordinates (0, 0, 0), the Cα atom of residue V806 (wild-type numbering) is at coordinates (0, *y*, 0), and the CYT center of mass is at coordinates (*x*, *y*, 0). Subsequently, GROMACS topology files were created with pdb2gmx for the cleaned models using the simple point charge (SPC) water model and the OPLS-AA force field. Definitions for restraining the positions of the DH domain atoms with a force constant of 104 kJ mol^–1^ were manually added to the topology files. Then, the models were centered at (*x*, *y*, *z*) coordinates (6.894, 6.894, 6.894 nm) in a box with (*x*, *y*, *z*) dimensions (26.000, 13.787, 13.787 nm). Once the models were solvated in SPC water and 0.1 M NaCl was added on top of neutralizing counterions with genion, a steepest descent energy minimization was performed with a convergence criterion of 103 kJ mol^–1^ nm^–1^. Thereafter, an *NPT* equilibration of 100 ps (5 × 10^4^ with a step size of 2 × 10^–3^) was performed with initial velocities generated from a Maxwell–Boltzmann distribution. To maintain a constant temperature of 310 K and a constant pressure of 1 bar, a weak coupling scheme with coupling times τ_T_ = 0.1 and τ_P_ = 2 ps, and isothermal compressibility of 4.5 × 10^–5^ bar^–1^ was used. The LINCS algorithm was used to constrain all bond lengths. A Verlet cutoff scheme was used and a new neighbor list was generated every 5 steps with a short range, electrostatic, and van der Waals cutoff of 1.4 nm. The Particle-Mesh Ewald (PME) method was used with PME order = 4 and Fourier spacing = 0.16 nm to treat long-range electrostatics. Subsequently, a steered MD simulation was performed with some modifications. Here, a new neighbor list was generated every 20 steps. Also, the Nose–Hoover method was used for the temperature coupling with τ_T_ = 1 ps, and the Parrinello–Rahman method was used for the pressure coupling with τ_P_ = 2 ps, and isothermal compressibility of 4.5 × 10^–5^ bar^–1^. During the steered MD simulations, position restraints 104 kJ mol^–1^ nm^–2^ were imposed on the DH domain and the CYT was constantly pulled away from the DH domain with a force constant of 500 kJ mol^–1^ nm^–2^ along the *x*-axis. Finally, the trajectories were analyzed with the tools provided in the GROMACS 2021.4 biomolecular simulation package. The data was then further processed using the python packages matplotlib, numpy, and scipy. A Savitzky–Golay filter, as implemented in scipy, was applied to the distance data with a filter window length of 101 and a polynomial of the second order.

## Supporting Information Available

Primer sequences, protein sequence information, additional sequence similarity network analysis, phylogenetic tree analysis, sequence conservation analysis, modeling, spectral and electrochemical properties are given as supporting information (PDF)
