# Supplementary material for: Interdomain Linker of the Bioelecrocatalyst Cellobiose Dehydrogenase Governs the Electron Transfer
Source: ACS Catal. 2023 Jun 5;13(12):8195–205. doi: 10.1021/acscatal.3c02116 (PMC10278072; doi:10.1021/acscatal.3c02116)
Supplement: Supplementary file 1 — cs3c02116_si_001.pdf [file cs3c02116_si_001.pdf]

Supplementary Information

The interdomain linker of the bioelecrocatalyst cellobise dehydrogenase governs the electron transfer

Lan Zhang[1], Christophe V. F. P. Laurent[1, 2], Lorenz Schwaiger[1], Lushan Wang[3], Su Ma[1, 3*], Roland Ludwig[1]

[1]*Department of Food Science and Technology, Biocatalysis and Biosensing Laboratory, University of Natural Resources and Life Sciences (BOKU), Vienna, Muthgasse 18, 1190, Vienna Austria*

[2]*Institute of Molecular Modeling and Simulation, Department of Material Sciences and Process Engineering, University of Natural Resources and Life Sciences (BOKU), Vienna, Muthgasse 18, 1190, Vienna, Austria*

[3]*State Key Laboratory of Microbial Technology, Shandong University, Binhai Road 72/N2, 266237 Qingdao, China*

[*] *Su Ma, masu@sdu.edu.cn*

Contents:

Supplementary figures S1–15
Supplementary tables S1–4

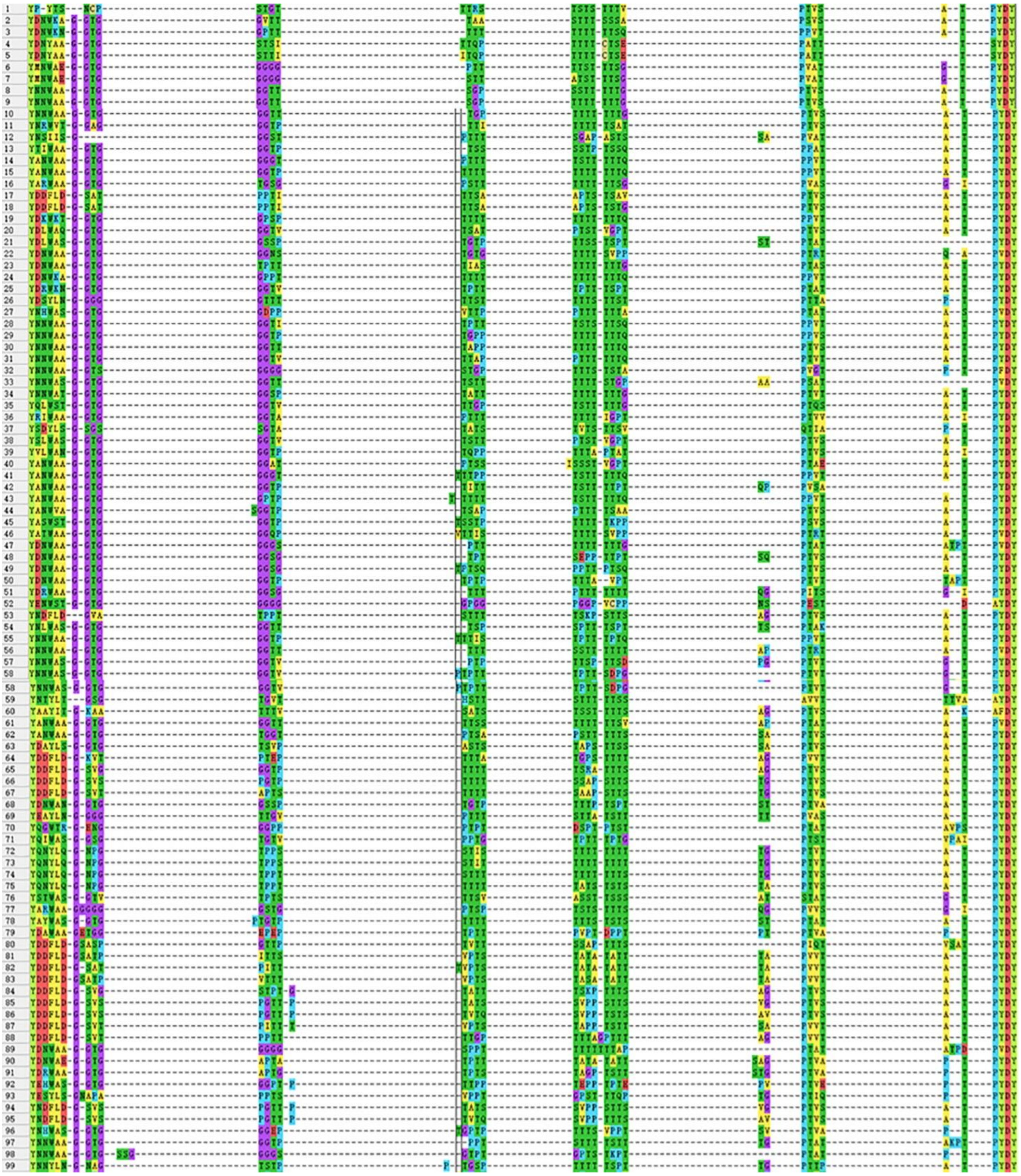

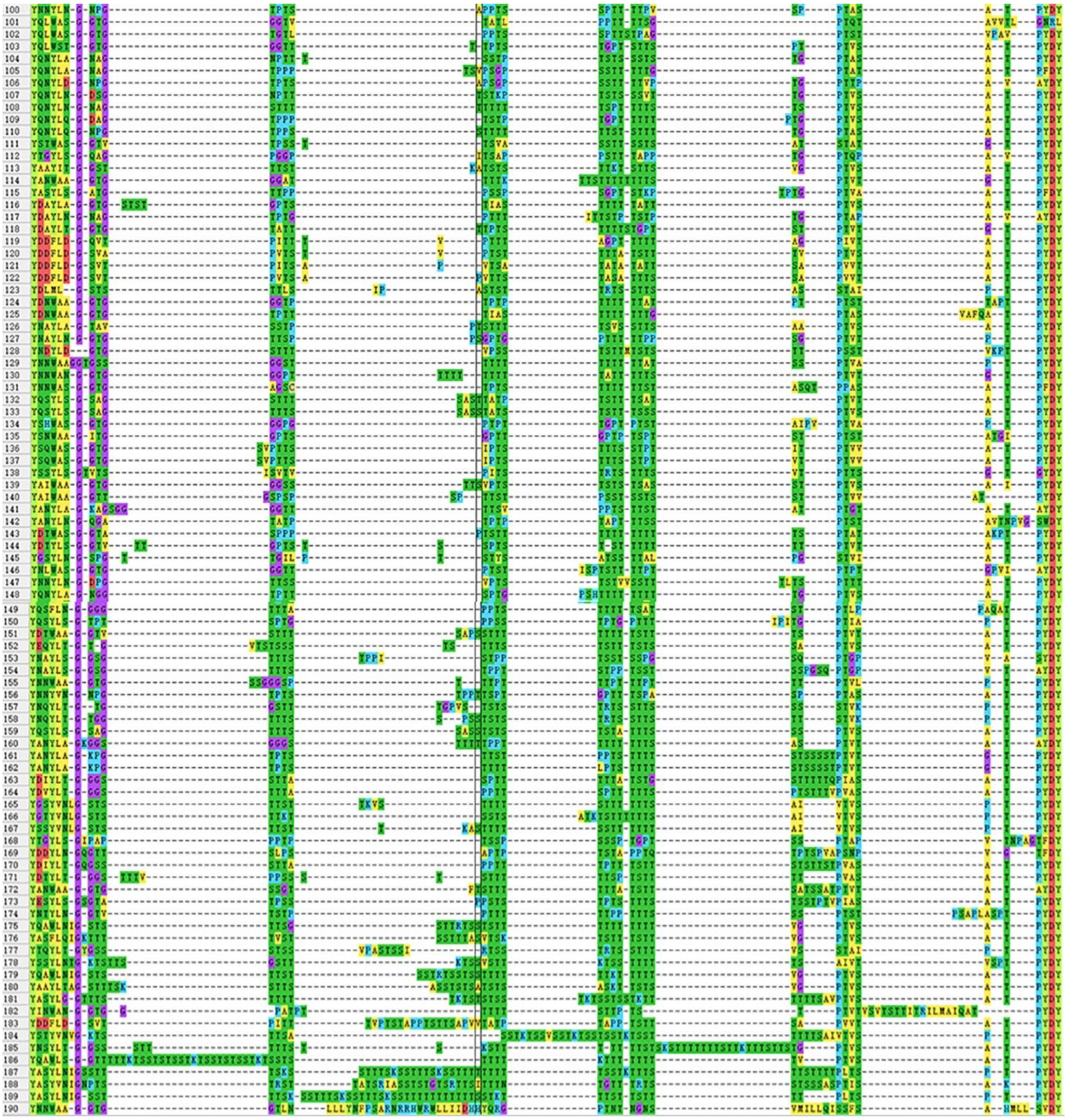

Figure S1. Multiple sequence alignment of 190 Class I CDH linkers sorted by increasing linker length. The sequence identifiers and additional information to the numbered sequences are given in Table S1. The borders of the linker were defined after the alignment and non-linker positions removed. The sequence alignment was performed with MEGA 11 using the MUSCLE algorithm. Hydrophilic amino acids are green, hydrophobic are yellow, acidic amino acids are red, lysines and prolines are blue, glycines are purple, and cysteines are brown.

Table S1. Sequence number, sequence identifiers and source information of 190 Class I CDH sequences used in the MSA shown in Figure S1. All sequences possess a cytochrome domain to and are verified CDHs contrary to what is indicated by some of the assigned names (Seq. 9, 10, 57 pyranose dehydrogenase, Seq. 15, 19, 42, 48, 127, 130, 172 substrate-specific activator of APC-dependent proteolysis).

<table>
<tr><td>1</td><td>>KAG8925730.1 hypothetical protein FRC02_009455 Tulasnella sp. 418</td></tr>
<tr><td>2</td><td>>KAG5635006.1 substrate-specific activator of APC-dependent proteolysis Sphagnurus paluster</td></tr>
<tr><td>3</td><td>>KAF8066812.1 cellobiose dehydrogenase Lyophyllum atratum</td></tr>
<tr><td>4</td><td>>KAH8827571.1 cellobiose dehydrogenase Flagelloscypha sp. PMI_526</td></tr>
<tr><td>5</td><td>>KAH8806689.1 hypothetical protein DL96DRAFT_1698942 Flagelloscypha sp. PMI_526</td></tr>
<tr><td>6</td><td>>tr|A0A0W0F2T1|A0A0W0F2T1_9AGAR Uncharacterized protein OS=Moniliophthora roreri OX=221103 GN=WG66_16822 PE=3 SV=1</td></tr>
<tr><td>7</td><td>>tr|V2YCQ0|V2YCQ0_MONRO Cellobiose dehydrogenase OS=Moniliophthora roreri (strain MCA 2997) OX=1381753 GN=Moror_16128 PE=3 SV=1</td></tr>
<tr><td>8</td><td>>KAF5354111.1 hypothetical protein D9756_007315 Leucoagaricus leucothites</td></tr>
<tr><td>9</td><td>>tr|A0A137QN87|A0A137QN87_9AGAR Pyranose dehydrogenase (acceptor) OS=Leucoagaricus sp. SymC.cos OX=1714833 GN=AN958_06218 PE=3 SV=1</td></tr>
<tr><td>10</td><td>>tr|A0A137QN22|A0A137QN22_9AGAR Pyranose dehydrogenase (acceptor) OS=Leucoagaricus sp. SymC.cos OX=1714833 GN=AN958_06217 PE=3 SV=1</td></tr>
<tr><td>11</td><td>>KAF5354113.1 hypothetical protein D9756_007317 Leucoagaricus leucothites</td></tr>
<tr><td>12</td><td>>tr|A0A4Y7Q0E4|A0A4Y7Q0E4_9AGAM Cellobiose dehydrogenase OS=Rickenella mellea OX=50990 GN=BD410DRAFT_316022 PE=3 SV=1</td></tr>
<tr><td>13</td><td>>tr|A0A369K4Y4|A0A369K4Y4_HYPMA Cellobiose dehydrogenase OS=Hypsizygus marmoreus OX=39966 GN=CDH-1_0 PE=3 SV=1</td></tr>
<tr><td>14</td><td>>KAF5381789.1 hypothetical protein D9615_005498 Tricholomella constricta</td></tr>
<tr><td>15</td><td>>KAG6918085.1 substrate-specific activator of APC-dependent proteolysis Tephrocybe rancida</td></tr>
<tr><td>16</td><td>>tr|V2X181|V2X181_MONRO Cellobiose dehydrogenase OS=Moniliophthora roreri (strain MCA 2997) OX=1381753 GN=Moror_1980 PE=3 SV=1</td></tr>
<tr><td>17</td><td>>KAF8596985.1 cellobiose dehydrogenase Ceratobasidium sp. AG-I</td></tr>
<tr><td>18</td><td>>KAF8596986.1 hypothetical protein BDV93DRAFT_527730 Ceratobasidium sp. AG-I</td></tr>
<tr><td>19</td><td>>KAG6918092.1 substrate-specific activator of APC-dependent proteolysis Tephrocybe rancida</td></tr>
<tr><td>20</td><td>>tr|A0A409YAD4|A0A409YAD4_9AGAR Uncharacterized protein OS=Panaeolus cyanescens OX=181874 GN=CVT24_009541 PE=3 SV=1</td></tr>
<tr><td>21</td><td>>KAF8908090.1 hypothetical protein CPB84DRAFT_1674456 Gymnopilus junonius</td></tr>
<tr><td>22</td><td>>KAF7313791.1 Cellobiose dehydrogenase Mycena chlorophos</td></tr>
<tr><td>23</td><td>>KAF8905671.1 hypothetical protein CPB85DRAFT_1437210 Mucidula mucida</td></tr>
<tr><td>24</td><td>>KAF5381773.1 hypothetical protein D9615_005492 Tricholomella constricta</td></tr>
<tr><td>25</td><td>>tr|A0A2A9NM86|A0A2A9NM86_9AGAR GMC_OxRdtase_N domain-containing protein OS=Amanita thiersii Skay4041 OX=703135 GN=AMATHDRAFT_58825 PE=4 SV=1</td></tr>
<tr><td>26</td><td>>KAH7102802.1 cellobiose dehydrogenase partial Auriculariales sp. MPI-PUGE-AT-0066</td></tr>
<tr><td>27</td><td>>tr|A0A291NNK4|A0A291NNK4_9AGAR Cellobiose dehydrogenase (Fragment) OS=Volvariella volvacea OX=36659 PE=3 SV=1</td></tr>
<tr><td>28</td><td>>KAF9465413.1 cellobiose dehydrogenase Lepista nuda</td></tr>
<tr><td>29</td><td>>KAF9465397.1 cellobiose dehydrogenase Lepista nuda</td></tr>
<tr><td>30</td><td>>KAF9447347.1 hypothetical protein P691DRAFT_782546 Macrolepiota fuliginosa MF-IS2</td></tr>
<tr><td>31</td><td>>KAF9447345.1 hypothetical protein P691DRAFT_760869 Macrolepiota fuliginosa MF-IS2</td></tr>
<tr><td>32</td><td>>tr|A0A5C3LY38|A0A5C3LY38_9AGAR Cellobiose dehydrogenase OS=Crucibulum laeve OX=68775 GN=BDQ12DRAFT_608330 PE=3 SV=1</td></tr>
<tr><td>33</td><td>>KAF7377697.1 Cellobiose dehydrogenase Mycena sanguinolenta</td></tr>
<tr><td>34</td><td>>tr|A0A137QN29|A0A137QN29_9AGAR Pyranose dehydrogenase (acceptor) OS=Leucoagaricus sp. SymC.cos OX=1714833 GN=AN958_06216 PE=3 SV=1</td></tr>
<tr><td>35</td><td>>KAF9479511.1 hypothetical protein BDN70DRAFT_858359 Pholiota conissans</td></tr>
<tr><td>36</td><td>>KAF8154680.1 cellobiose dehydrogenase Crassisporium funariophilum</td></tr>
<tr><td>37</td><td>>KAG8962795.1 hypothetical protein FRC03_003784 Tulasnella sp. 419</td></tr>
<tr><td>38</td><td>>KAF9042914.1 cellobiose dehydrogenase Panaeolus papilionaceus</td></tr>
<tr><td>39</td><td>>CAA7262555.1 unnamed protein product Agrocybe aegerita</td></tr>
<tr><td>40</td><td>>KAF9008074.1 hypothetical protein BDQ17DRAFT_1455887 Cyathus striatus</td></tr>
<tr><td>41</td><td>>KAF8066822.1 cellobiose dehydrogenase Lyophyllum atratum</td></tr>
<tr><td>42</td><td>>tr|A0A369JZK7|A0A369JZK7_HYPMA Cellobiose dehydrogenase OS=Hypsizygus marmoreus OX=39966 GN=CDH-1_2 PE=4 SV=1</td></tr>
<tr><td>43</td><td>>KAG6833025.1 substrate-specific activator of APC-dependent proteolysis Tephrocybe sp. NHM501043</td></tr>
<tr><td>44</td><td>>tr|A0A2H3BZE2|A0A2H3BZE2_9AGAR Cellobiose dehydrogenase OS=Armillaria solidipes OX=1076256 GN=ARMSODRAFT_994828 PE=3 SV=1</td></tr>
<tr><td>45</td><td>>KAF5310212.1 hypothetical protein D9619_010476 Psilocybe cf. subviscida</td></tr>
<tr><td>46</td><td>>KAF7324781.1 Cellobiose dehydrogenase Mycena kentingensis (nom. inval.)</td></tr>
<tr><td>47</td><td>>tr|A0A550C2M7|A0A550C2M7_9AGAR Cellobiose dehydrogenase OS=Auriculariopsis ampla OX=97359 GN=BD626DRAFT_559802 PE=3 SV=1</td></tr>
<tr><td>48</td><td>>KAF4607348.1 substrate-specific activator of APC-dependent proteolysis Pleurotus pulmonarius</td></tr>
<tr><td>49</td><td>>KAF9491048.1 cellobiose dehydrogenase Pleurotus eryngii</td></tr>
<tr><td>50</td><td>>tr|A0A4Q2DDM1|A0A4Q2DDM1_9AGAR Uncharacterized protein OS=Candolleomyces aberdarensis OX=2316362 GN=EST38_g8702 PE=3 SV=1</td></tr>
<tr><td>51</td><td>>XP_043033925.1 cellobiose dehydrogenase Guyanagaster necrorhizus MCA 3950</td></tr>
<tr><td>52</td><td>>tr|A0A5C3Q807|A0A5C3Q807_9AGAR Cellobiose dehydrogenase OS=Pterula gracilis OX=1884261 GN=BDV98DRAFT_255766 PE=3 SV=1</td></tr>
<tr><td>53</td><td>>KAG8687354.1 hypothetical protein FRC09_013548 Ceratobasidium sp. 395</td></tr>
<tr><td>54</td><td>>tr|A0A067SH97|A0A067SH97_GALM3 GMC_OxRdtase_N domain-containing protein OS=Galerina marginata (strain CBS 339.88) OX=685588 GN=GALMADRAFT_894992 PE=3 SV=1</td></tr>
<tr><td>55</td><td>>KAF9447344.1 hypothetical protein P691DRAFT_821966 Macrolepiota fuliginosa MF-IS2</td></tr>
<tr><td>56</td><td>>KAF7369026.1 Cellobiose dehydrogenase Mycena venus</td></tr>
<tr><td>57</td><td>>tr|K5XS03|K5XS03_AGABU Pyranose dehydrogenase (acceptor) OS=Agaricus bisporus var. burnettii (strain JB137-S8 / ATCC MYA-4627 / FGSC 10392) OX=597362 GN=AGABI1DRAFT_107890 PE=4 SV=1</td></tr>
<tr><td>58</td><td>>KAF7760214.1 CAZyme family AA8 Agaricus bisporus var. burnettii</td></tr>
<tr><td>59</td><td>>KAF8307890.1 cellobiose dehydrogenase Clavulina sp. PMI_390</td></tr>
<tr><td>60</td><td>>KAG8994110.1 hypothetical protein FRB94_010106 Tulasnella sp. JGI-2019a</td></tr>
<tr><td>61</td><td>>KAF8201091.1 cellobiose dehydrogenase Mycena galopus ATCC 62051</td></tr>
<tr><td>62</td><td>>tr|A0A2H3DNC5|A0A2H3DNC5_ARMGA Cellobiose dehydrogenase OS=Armillaria gallica OX=47427 GN=ARMGADRAFT_1084055 PE=3 SV=1</td></tr>
<tr><td>63</td><td>>tr|W4JPN3|W4JPN3_HETIT Cellobiose dehydrogenase 1 OS=Heterobasidion irregulare (strain TC 32-1) OX=747525 GN=cdh1 PE=3 SV=1</td></tr>
<tr><td>64</td><td>>tr|M5BPA3|M5BPA3_THACB Cellobiose dehydrogenase Short=CDH OS=Thanatephorus cucumeris (strain AG1-IB / isolate 7/3/14) OX=1108050 GN=BN14_02153 PE=4 SV=1</td></tr>
<tr><td>65</td><td>>KAG9127847.1 hypothetical protein FRC07_008490 Ceratobasidium sp. 392</td></tr>
<tr><td>66</td><td>>KAG8697384.1 hypothetical protein FRC08_006563 Ceratobasidium sp. 394</td></tr>
<tr><td>67</td><td>>KAF8605228.1 cellobiose dehydrogenase Ceratobasidium sp. AG-I</td></tr>
<tr><td>68</td><td>>tr|A0A409VN63|A0A409VN63_9AGAR GMC_OxRdtase_N domain-containing protein OS=Gymnopilus dilepis OX=231916 GN=CVT26_007421 PE=3 SV=1</td></tr>
<tr><td>69</td><td>>EJD48894.1 cellobiose dehydrogenase Auricularia subglabra TFB-10046 SS5</td></tr>
<tr><td>70</td><td>>TFK65977.1 cellobiose dehydrogenase Pluteus cervinus</td></tr>
<tr><td>71</td><td>>KAF9524951.1 cellobiose dehydrogenase Crepidotus variabilis</td></tr>
<tr><td>72</td><td>>tr|A0A371CMX0|A0A371CMX0_9APHY Cellobiose dehydrogenase OS=Polyporus brumalis OX=139420 GN=OH76DRAFT_1364243 PE=3 SV=1</td></tr>
<tr><td>73</td><td>>tr|A0A5C3P545|A0A5C3P545_9APHY Uncharacterized protein OS=Polyporus arcularius HHB13444 OX=1314778 GN=K466DRAFT_664950 PE=3 SV=1</td></tr>
<tr><td>74</td><td>>tr|A0A5C2TVM7|A0A5C2TVM7_9APHY Uncharacterized protein OS=Lentinus tigrinus ALCF2SS1-7 OX=1328758 GN=L226DRAFT_550747 PE=3 SV=1</td></tr>
<tr><td>75</td><td>>tr|A0A2G8S0Y0|A0A2G8S0Y0_9APHY GMC_OxRdtase_N domain-containing protein OS=Ganoderma sinense ZZ0214-1 OX=1077348 GN=GSI_10574 PE=4 SV=1</td></tr>
<tr><td>76</td><td>>tr|A0A166QY38|A0A166QY38_9AGAM Uncharacterized protein OS=Fibularhizoctonia sp. CBS 109695 OX=436010 GN=FIBSPDRAFT_818085 PE=3 SV=1</td></tr>
<tr><td>77</td><td>>KAF9269255.1 cellobiose dehydrogenase Marasmius fiardii PR-910</td></tr>
<tr><td>78</td><td>>KAG5723688.1 Cellobiose dehydrogenase Termitomyces sp. T112</td></tr>
<tr><td>79</td><td>>tr|A0A4Y7ST68|A0A4Y7ST68_9AGAR Cellobiose dehydrogenase OS=Coprinellus micaceus OX=71717 GN=FA13DRAFT_1756701 PE=3 SV=1</td></tr>
<tr><td>80</td><td>>KAG8745775.1 hypothetical protein FRC10_007036 Ceratobasidium sp. 414</td></tr>
<tr><td>81</td><td>>CAE6491931.1 unnamed protein product Rhizoctonia solani</td></tr>
<tr><td>82</td><td>>KAG8688196.1 hypothetical protein FRC11_005887 Ceratobasidium sp. 423</td></tr>
<tr><td>83</td><td>>CAE6465288.1 unnamed protein product Rhizoctonia solani</td></tr>
<tr><td>84</td><td>>KAG8748222.1 hypothetical protein FRC12_013866 Ceratobasidium sp. 428</td></tr>
<tr><td>85</td><td>>QRV87512.1 GMC oxidoreductase Ceratobasidium sp. AG-Ba</td></tr>
<tr><td>86</td><td>>QRV98090.1 GMC oxidoreductase Ceratobasidium sp. AG-Ba</td></tr>
<tr><td>87</td><td>>KAH7343509.1 cellobiose dehydrogenase Rhizoctonia solani</td></tr>
<tr><td>88</td><td>>KAF8742837.1 Cellobiose dehydrogenase partial Rhizoctonia solani</td></tr>
</table>

89	>tr|D8QLR8|D8QLR8_SCHCM Cellobiose dehydrogenase (Fragment) OS=Schizophyllum commune (strain H4-8 / FGSC 9210) OX=578458 GN=SCHCODRAFT_114791 PE=3 SV=1
90	>tr|A0A0D7AWI7|A0A0D7AWI7_9AGAR Uncharacterized protein OS=Cylindrobasidium torrendii FP15055 ss-10 OX=1314674 GN=CYLTODRAFT_185011 PE=3 SV=1
91	>tr|A0A0D7BGV1|A0A0D7BGV1_9AGAR Uncharacterized protein OS=Cylindrobasidium torrendii FP15055 ss-10 OX=1314674 GN=CYLTODRAFT_420700 PE=3 SV=1
92	>tr|A8NN13|A8NN13_COPC7 Cellobiose dehydrogenase OS=Coprinopsis cinerea (strain Okayama-7 / 130 / ATCC MYA-4618 / FGSC 9003) OX=240176 GN=CC1G_09923 PE=3 SV=2
93	>tr|S5R9V1|S5R9V1_CERUI Cellobiose dehydrogenase OS=Cerrena unicolor OX=90312 PE=2 SV=1
94	>QRV72643.1 GMC oxidoreductase Ceratobasidium sp. AG-Ba
95	>QRW12680.1 GMC oxidoreductase Ceratobasidium sp. AG-Ba
96	>KAH6904290.1 cellobiose dehydrogenase Coprinopsis sp. MPI-PUGE-AT-0042
97	>tr|A0A4S8M8T0|A0A4S8M8T0_DENBC Cellobiose dehydrogenase OS=Dendrothele bispora (strain CBS 962.96) OX=1314807 GN=K435DRAFT_838037 PE=3 SV=1
98	>KAF5390938.1 hypothetical protein D9757_003923 Gymnopus confluens
99	>tr|Q8J2T4|Q8J2T4_GRIFR Cellobiose dehydrogenase OS=Grifola frondosa OX=5627 GN=cdh PE=2 SV=1
100	>OCH89474.1 cellobiose dehydrogenase Obba rivulosa
101	>KAF8958430.1 hypothetical protein BDZ97DRAFT_1906522 Flammula alnicola
102	>KAF9525707.1 cellobiose dehydrogenase Crepidotus variabilis
103	>KAF8191334.1 cellobiose dehydrogenase Pholiota molesta
104	>tr|K5UTR2|K5UTR2_PHACS Uncharacterized protein OS=Phanerochaete carnosa (strain HHB-10118-sp) OX=650164 GN=PHACADRAFT_259608 PE=3 SV=1
105	>XP_008041466.1 cellobiose dehydrogenase Trametes versicolor FP-101664 SS1
106	>tr|Q6AW20|Q6AW20_IRPLA Cellobiose dehydrogenase OS=Irpex lacteus OX=5319 GN=cdh PE=2 SV=1
107	>sp|Q01738|CDH_PHACH Cellobiose dehydrogenase OS=Phanerodontia chrysosporium OX=2822231 GN=CDH-1 PE=1 SV=1
108	>tr|A0A0C3PUG7|A0A0C3PUG7_PHLGI Cellobiose dehydrogenase OS=Phlebiopsis gigantea 11061_1 CR5-6 OX=745531 GN=PHLGIDRAFT_99876 PE=3 SV=1
109	>tr|S5RVR8|S5RVR8_9APHY Cellobiose dehydrogenase OS=Trametes sanguinea OX=158606 PE=2 SV=1
110	>XP_007363678.1 cellobiose dehydrogenase Dichomitus squalens LYAD-421 SS1
111	>tr|A0A166R9D3|A0A166R9D3_9AGAM Uncharacterized protein OS=Fibularhizoctonia sp. CBS 109695 OX=436010 GN=FIBSPDRAFT_1039750 PE=3 SV=1
112	>tr|M1H9C3|M1H9C3_9APHY Cellobiose dehydrogenase OS=Phlebia lindtneri OX=98769 GN=cdh PE=3 SV=1
113	>KAG8878925.1 hypothetical protein FRB98_005908 Tulasnella sp. 332
114	>KAG6820015.1 substrate-specific activator of APC-dependent proteolysis Arthromyces matolae
115	>KAH8103281.1 cellobiose dehydrogenase Cristinia sonorae
116	>tr|A0A4S4LGC3|A0A4S4LGC3_9AGAM Uncharacterized protein OS=Bondarzewia mesenterica OX=1095465 GN=EW146_g8858 PE=3 SV=1
117	>tr|A0A2R6P0X5|A0A2R6P0X5_9APHY Uncharacterized protein OS=Phlebia centrifuga OX=98765 GN=PHLCEN_2v6153 PE=3 SV=1
118	>XP_007306783.1 hypothetical protein STEHIDRAFT_62168 Stereum hirsutum FP-91666 SS1
119	>CAE6396487.1 unnamed protein product Rhizoctonia solani
120	>KAB5595318.1 Cellobiose dehydrogenase Ceratobasidium theobromae
121	>tr|A0A0K6FT39|A0A0K6FT39_9AGAM Cellobiose dehydrogenase OS=Rhizoctonia solani OX=456999 GN=RSOLAG22IIIB_03935 PE=4 SV=1
122	>tr|X8JU14|X8JU14_9AGAM Cellobiose dehydrogenase OS=Rhizoctonia solani AG-3 Rhs1AP OX=1086054 GN=RSOL_476920 PE=4 SV=1
123	>tr|A0A0C2X722|A0A0C2X722_9AGAM Cellobiose dehydrogenase protein OS=Serendipita vermifera MAFF 305830 OX=933852 GN=M408DRAFT_87040 PE=3 SV=1
124	>KAF6760392.1 cellobiose dehydrogenase Tulosesus angulatus
125	>KAF9038951.1 hypothetical protein BDZ89DRAFT_945553 Hymenopellis radicata
126	>tr|A0A067PDP4|A0A067PDP4_9AGAM Carbohydrate-binding module 1 protein OS=Jaapia argillacea MUCL 33604 OX=933084 GN=JAAARDRAFT_210324 PE=3 SV=1
127	>tr|A0A4R0RBC4|A0A4R0RBC4_9APHY Substrate-specific activator of APC-dependent proteolysis OS=Steccherinum ochraceum OX=92696 GN=CDH1_1 PE=3 SV=1
128	>PVG01966.1 putative cellobiose dehydrogenase Serendipita vermifera subsp. bescii
129	>KAH7869806.1 cellobiose dehydrogenase Lentinula edodes
130	>KAG6828939.1 substrate-specific activator of APC-dependent proteolysis Tricholoma furcatifolium
131	>KAF9004636.1 hypothetical protein BDQ17DRAFT_1390151 Cyathus striatus
132	>tr|A0A0C9V5G4|A0A0C9V5G4_9AGAM Unplaced genomic scaffold scaffold_35 whole genome shotgun sequence OS=Hydnomerulius pinastri MD-312 OX=994086 GN=HYDPIDRAFT_31984 PE=4 SV=1
133	>KAH7920130.1 hypothetical protein BV22DRAFT_823461 Leucogyrophana mollusca
134	>tr|A0A5C3KV64|A0A5C3KV64_9AGAR Cellobiose dehydrogenase OS=Coprinopsis marcescibilis OX=230819 GN=FA15DRAFT_704862 PE=3 SV=1
135	>KAF8874745.1 hypothetical protein BD779DRAFT_1678607 Infundibulicybe gibba
136	>KAF4610181.1 hypothetical protein D9613_010591 Agrocybe pediades
137	>KAF9561718.1 cellobiose dehydrogenase Agrocybe pediades
138	>tr|A0A0C2XXZ6|A0A0C2XXZ6_9AGAM Cellobiose dehydrogenase protein OS=Serendipita vermifera MAFF 305830 OX=933852 GN=M408DRAFT_59550 PE=4 SV=1
139	>KAG5167187.1 hypothetical protein JR316_007527 Psilocybe cubensis
140	>tr|A0A409X1T7|A0A409X1T7_PSICY Uncharacterized protein OS=Psilocybe cyanescens OX=93625 GN=CVT25_014208 PE=3 SV=1
141	>tr|A0A166J651|A0A166J651_9AGAM Uncharacterized protein OS=Peniophora sp. CONT OX=1314672 GN=PENSPDRAFT_710401 PE=3 SV=1
142	>tr|A0A0C9TMQ2|A0A0C9TMQ2_SPHS4 Unplaced genomic scaffold SPHSTscaffold_170 whole genome shotgun sequence OS=Sphaerobolus stellatus (strain SS14) OX=990650 GN=M422DRAFT_186233 PE=4 S
143	>KAF5366213.1 hypothetical protein D9758_005728 Tetrapyrgos nigripes
144	>XP_007387326.1 cellobiose dehydrogenase Punctularia strigosozonata HHB-11173 SS5
145	>tr|F8PAL3|F8PAL3_SERL9 Putative cellobiose dehydrogenase OS=Serpula lacrymans var. lacrymans (strain S7.9) OX=578457 GN=SERLADRAFT_491377 PE=3 SV=1
146	>tr|A0A0D2KUE6|A0A0D2KUE6_HYPSF GMC_OxRdtase_N domain-containing protein OS=Hypholoma sublateritium (strain FD-334 SS-4) OX=945553 GN=HYPSUDRAFT_70019 PE=4 SV=1
147	>tr|F8PAL4|F8PAL4_SERL9 Cellobiose dehydrogenase OS=Serpula lacrymans var. lacrymans (strain S7.9) OX=578457 GN=CDH2 PE=3 SV=1
148	>GJE98251.1 cellobiose dehydrogenase Phanerochaete sordida
149	>tr|A0A166ACA5|A0A166ACA5_EXIGL Cellobiose dehydrogenase OS=Exidia glandulosa HHB12029 OX=1314781 GN=EXIGLDRAFT_720262 PE=4 SV=1
150	>tr|A0A4S4MRV6|A0A4S4MRV6_9APHY Uncharacterized protein OS=Antrodiella citrinella OX=2447956 GN=EUX98_g5306 PE=3 SV=1
151	>KII85600.1 hypothetical protein PLICRDRAFT_322412 Plicaturopsis crispa FD-325 SS-3
152	>tr|Q7Z975|Q7Z975_9AGAM Cellobiose dehydrogenase OS=Athelia rolfsii OX=39291 GN=CDH PE=2 SV=1
153	>tr|A0A5B1R9L2|A0A5B1R9L2_9AGAM GMC_OxRdtase_N domain-containing protein OS=Dentipellis sp. KUC8613 OX=1883078 GN=DENSPDRAFT_867739 PE=3 SV=1
154	>tr|A0A4Y9YU91|A0A4Y9YU91_9AGAM GMC_OxRdtase_N domain-containing protein OS=Dentipellis fragilis OX=205917 GN=EVG20_g5113 PE=3 SV=1
155	>tr|A0A0D0BIH1|A0A0D0BIH1_9AGAR Unplaced genomic scaffold GYMLUscaffold_14 whole genome shotgun sequence OS=Gymnopus luxurians FD-317 M1 OX=944289 GN=GYMLUDRAFT_241124 PE=3 S
156	>tr|M2QHU4|M2QHU4_CERS8 Cellobiose dehydrogenase OS=Ceriporiopsis subvermispora (strain B) OX=914234 GN=CERSUDRAFT_84792 PE=3 SV=1
157	>CAG7847998.1 Cellobiose dehydrogenase Short=CDH AltName: Full=Cellobiose-quinone oxidoreductase Flags: Precursor Serendipita indica DSM 11827
158	>CAG7848000.1 Cellobiose dehydrogenase Short=CDH AltName: Full=Cellobiose-quinone oxidoreductase Flags: Precursor Serendipita indica DSM 11827
159	>KAH7911765.1 putative cellobiose dehydrogenase Hygrophoropsis aurantiaca
160	>tr|A0A5E3WUP0|A0A5E3WUP0_9AGAM Uncharacterized protein OS=Peniophora sp. CBMAI 1063 OX=718367 GN=PNH_LOCUS7172 PE=3 SV=1
161	>tr|A0A5M3M9A5|A0A5M3M9A5_CONPW Cellobiose dehydrogenase OS=Coniophora puteana (strain RWD-64-598) OX=741705 GN=CONPUDRAFT_131886 PE=3 SV=1
162	>tr|Q6BDD5|Q6BDD5_9AGAM Cellobiose dehydrogenase OS=Coniophora puteana OX=80637 GN=cdh PE=2 SV=1
163	>OCB87941.1 cellobiose dehydrogenase Sanghuangporus baumii
164	>tr|A0A286U7K7|A0A286U7K7_9AGAM Cellobiose dehydrogenase OS=Pyrrhoderma noxium OX=2282107 GN=PNOK_0841600 PE=3 SV=1
165	>tr|A0A0C3M143|A0A0C3M143_9AGAM Cellobiose dehydrogenase protein OS=Tulasnella calospora MUT 4182 OX=1051891 GN=M407DRAFT_23412 PE=4 SV=1
166	>KAG9042620.1 hypothetical protein FS837_010622 Tulasnella sp. UAMH 9824
167	>KAG8927418.1 hypothetical protein FRC01_007517 Tulasnella sp. 417
168	>KAF8584272.1 hypothetical protein K439DRAFT_1150670 Ramaria rubella
169	>GJJ08611.1 hypothetical protein Clacol_002830 Clathrus columnatus
170	>XP_007264488.1 cellobiose dehydrogenase Fomitiporia mediterranea MF3/22
171	>KAH8115148.1 cellobiose dehydrogenase Phellopilus nigrolimitatus
172	>XP_043010643.1 substrate-specific activator of APC-dependent proteolysis Marasmius oreades
173	>tr|A0A4S4LAX7|A0A4S4LAX7_9AGAM Uncharacterized protein OS=Phellinidium pouzarii OX=167371 GN=EW145_g2494 PE=3 SV=1
174	>tr|A0A0H2RQB0|A0A0H2RQB0_9AGAM Cellobiose dehydrogenase OS=Schizopora paradoxa OX=27342 GN=SCHPADRAFT_941881 PE=3 SV=1
175	>KAG9050367.1 hypothetical protein FS837_005988 Tulasnella sp. UAMH 9824
176	>KAG9000556.1 hypothetical protein FRB94_005345 Tulasnella sp. JGI-2019a
177	>KAG8761529.1 hypothetical protein FRC14_002716 Serendipita sp. 396
178	>KAG8947803.1 hypothetical protein FRC04_010289 Tulasnella sp. 424
179	>KAG8951577.1 hypothetical protein FRC04_005864 Tulasnella sp. 424
180	>KAG8875210.1 hypothetical protein FRB97_005323 Tulasnella sp. 331
181	>KAG9019815.1 hypothetical protein FRB90_005963 Tulasnella sp. 427
182	>KAG6873702.1 hypothetical protein C0995_012164 Termitomyces sp. Mi166008
183	>CAE6512615.1 unnamed protein product Rhizoctonia solani
184	>KAG9018158.1 hypothetical protein FRB90_012066 Tulasnella sp. 427
185	>tr|A0A164NFF7|A0A164NFF7_9AGAM Cellobiose dehydrogenase OS=Sistotremastrum niveocremeum HHB9708 OX=1314777 GN=SISNIDRAFT_553093 PE=3 SV=1
186	>KAG8998099.1 hypothetical protein FRB90_012380 Tulasnella sp. 427
187	>tr|A0A164NFE5|A0A164NFE5_9AGAM Cellobiose dehydrogenase OS=Sistotremastrum niveocremeum HHB9708 OX=1314777 GN=SISNIDRAFT_553092 PE=3 SV=1
188	>KAG8909001.1 hypothetical protein FRB99_000104 Tulasnella sp. 403
189	>tr|A0A165YUS4|A0A165YUS4_9AGAM Cellobiose dehydrogenase OS=Sistotremastrum suecicum HHB10207 ss-3 OX=1314776 GN=SISSUDRAFT_1132443 PE=3 SV=1
190	>XP_037220751.1 Cellobiose dehydrogenase Mycena indigotica

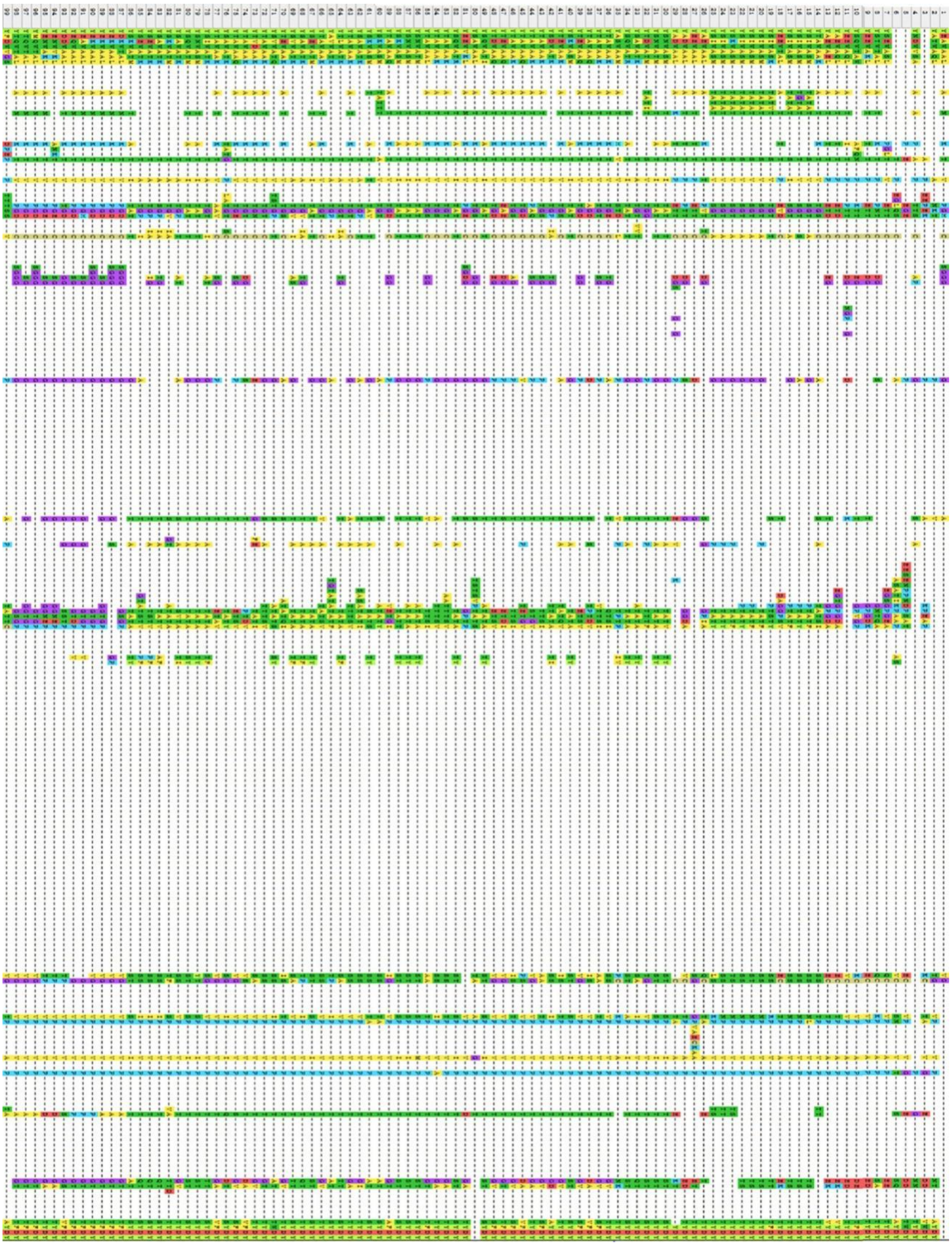

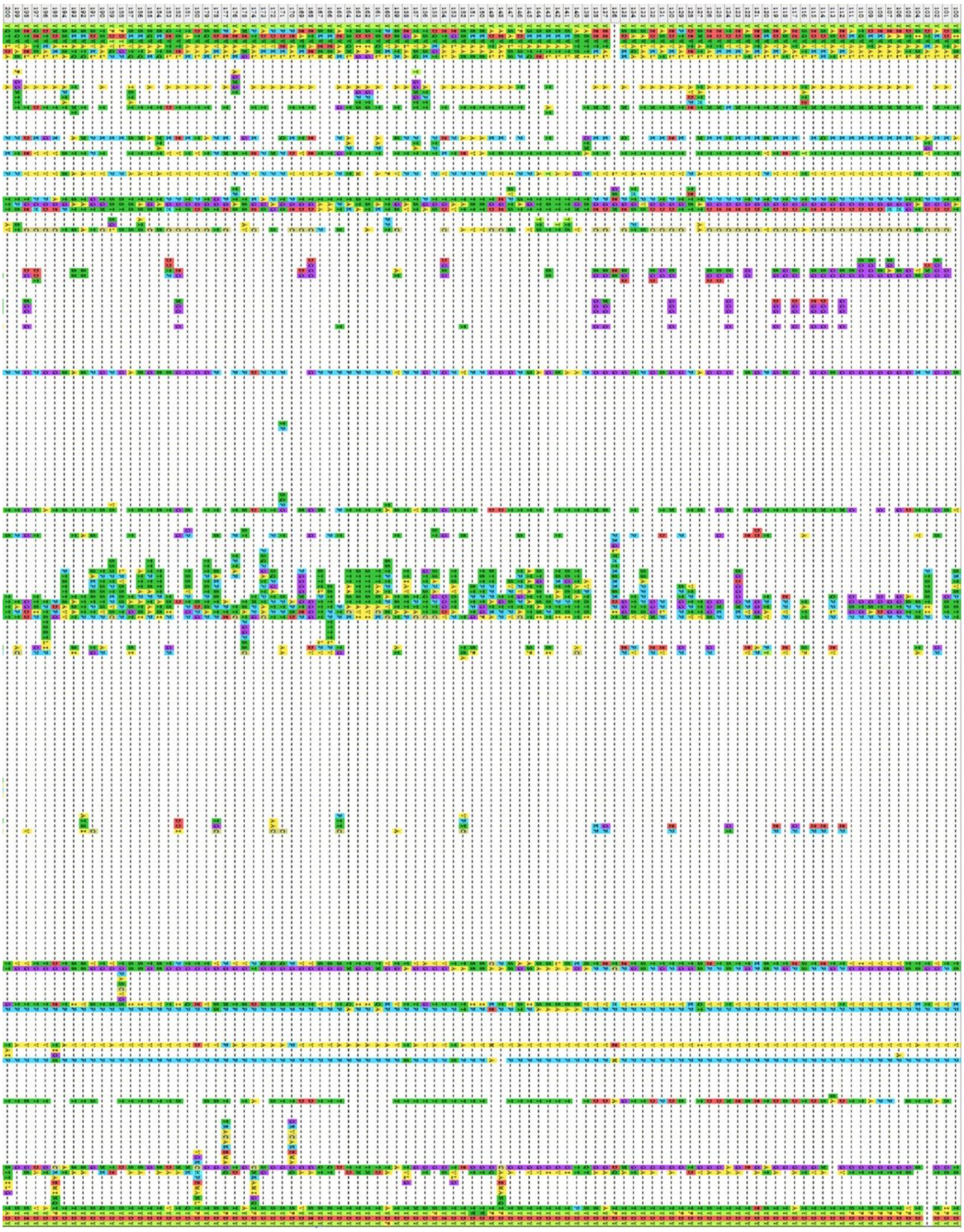

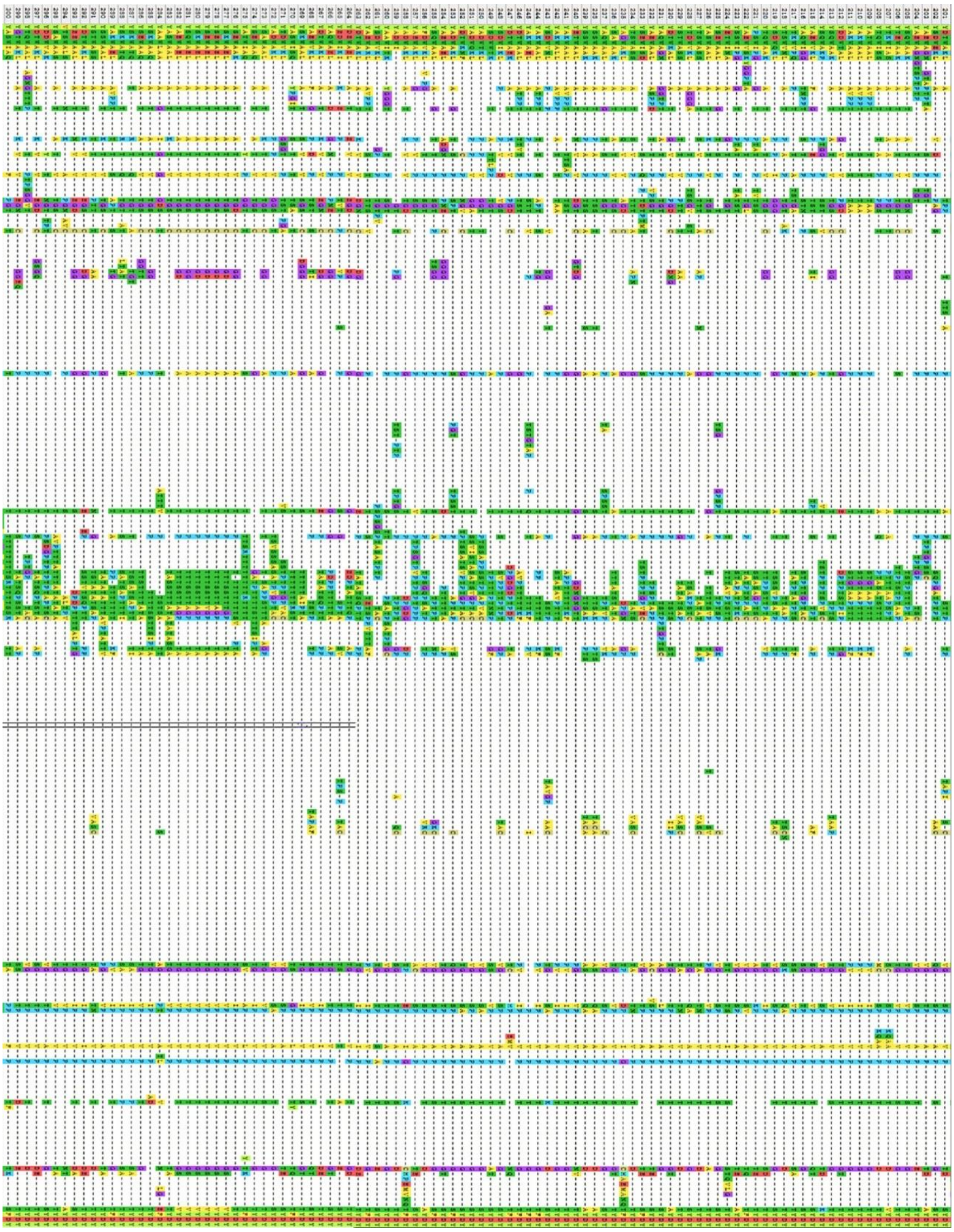

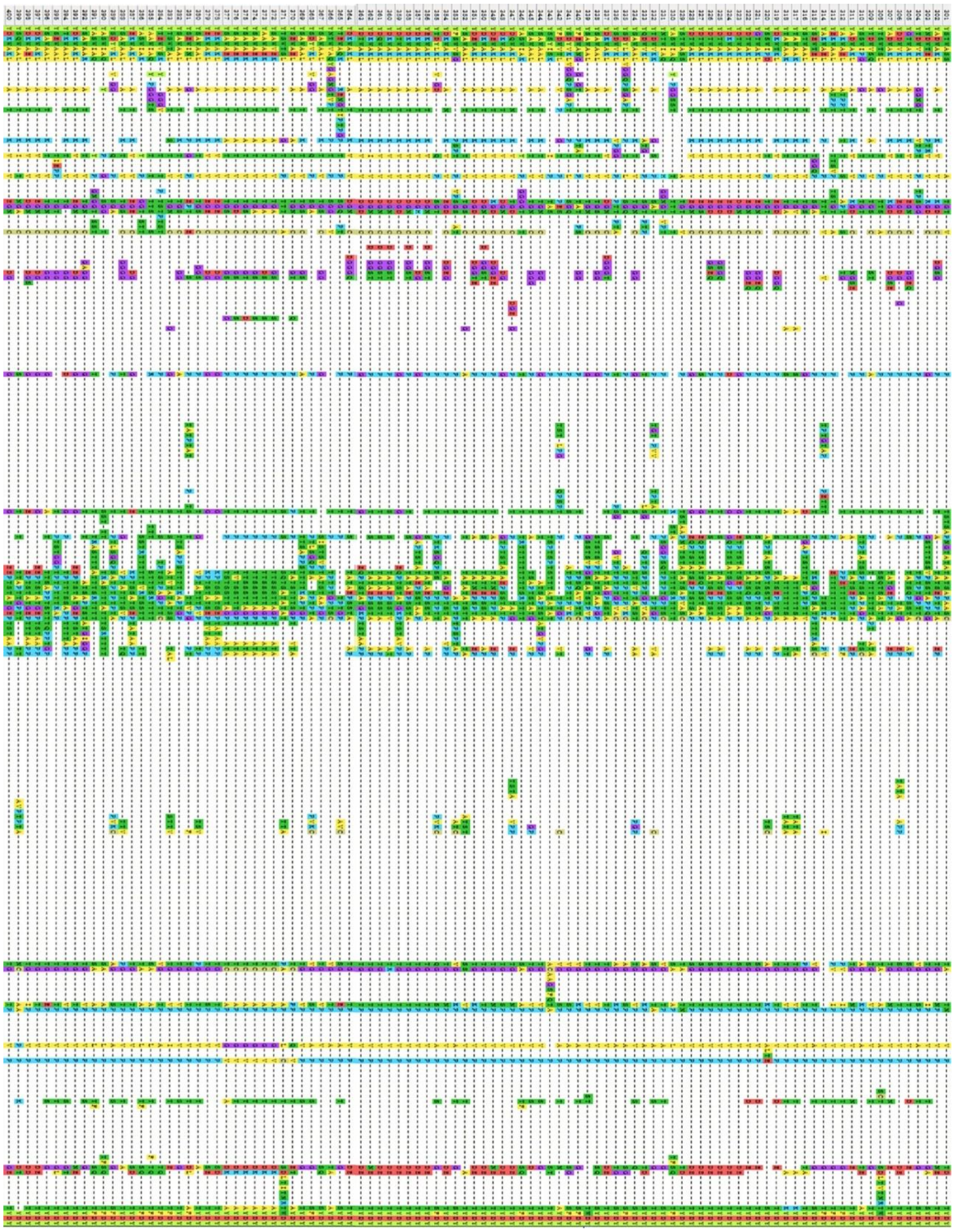

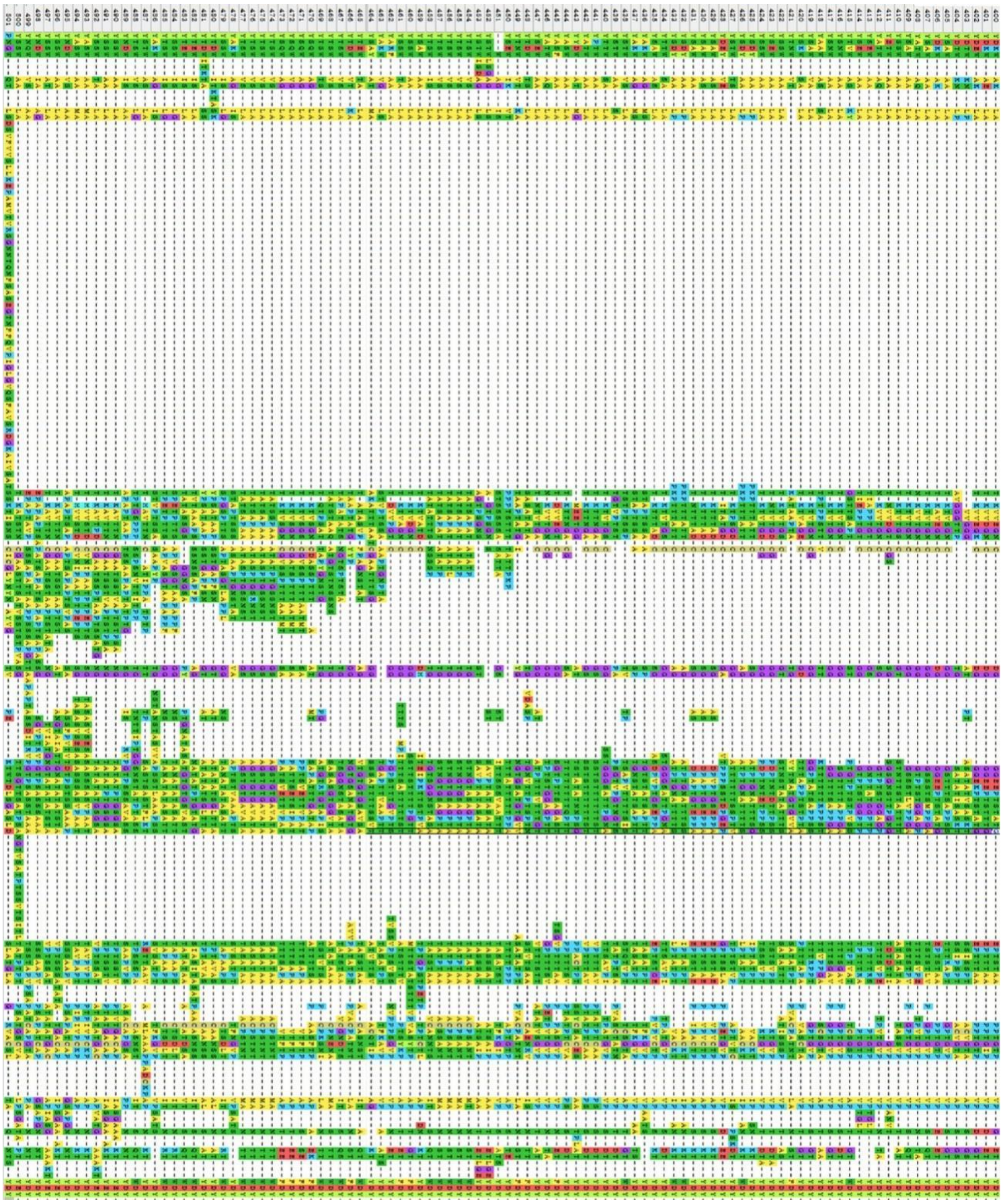

Figure S2. Multiple sequence alignment of 501 Class I CDH linkers sorted by increasing linker length. The sequence identifiers and additional information to the numbered sequences are given in Table S2. The borders of the linker were defined after the alignment and non-linker positions removed. The sequence alignment was performed with MEGA 11 using the MUSCLE algorithm. Hydrophilic amino acids are green, hydrophobic are yellow, acidic amino acids are red, lysines and prolines are blue, glycines are purple, and cysteines are brown.

Table S2. Sequence number, sequence identifiers and source information of 501 Class II CDH sequences used in the MSA shown in Figure S2. All sequences possess a cytochrome domain and are verified CDHs contrary to what is indicated by some of the assigned names (Seq. 2, 12, 34, 46, 86, 424, 431, 498).

<table>
<tr><td>1</td><td>>KAH2848663.1 hypothetical protein KXW36 006830 Aspergillus fumigatus</td></tr>
<tr><td>2</td><td>>KAF3351943.1 Zinc finger protein C25B8.19c Verticillium dahliae VDG1</td></tr>
<tr><td>3</td><td>>tr|A0A2N3N4U9|A0A2N3N4U9 9PEZI Uncharacterized protein OS=Lomentospora prolificans OX=41688 GN=jhhlp 006063 PE=3 SV=1</td></tr>
<tr><td>4</td><td>>KAF2220696.1 hypothetical protein BDZ85DRAFT 203325 Elsinoe ampelina</td></tr>
<tr><td>5</td><td>>tr|A0A084FZX3|A0A084FZX3 PSEDA GMC OxRdtase N domain-containing protein OS=Pseudallescheria apiosperma OX=563466 GN=SAPIO CDS8557 PE=3 SV=1</td></tr>
<tr><td>6</td><td>>KAF2966595.1 hypothetical protein GQX73 g6974 Xylaria multiplex</td></tr>
<tr><td>7</td><td>>tr|Q9P8H5|Q9P8H5 HUMIN Cellobiose dehydrogenase OS=Humicola insolens OX=34413 PE=2 SV=1</td></tr>
<tr><td>8</td><td>>tr|G2QV06|G2QV06 THETT Cellobiose dehydrogenase OS=Thermothielavioides terrestris (strain ATCC 38088 / NRRL 8126) OX=578455 GN=THITE 123924 PE=3 SV=1</td></tr>
<tr><td>9</td><td>>tr|G2QFY4|G2QFY4 MYCTT Cellobiose dehydrogenase OS=Myceliophthora thermophila (strain ATCC 42464 / BCRC 31852 / DSM 1799) OX=573729 GN=MYCTH 81925 PE=3 SV=1</td></tr>
<tr><td>10</td><td>>tr|G0S3B0|G0S3B0 CHATD Cellobiose dehydrogenase-like protein OS=Chaetomium thermophilum (strain DSM 1495 / CBS 144.50 / IMI 039719) OX=759272 GN=CTHT 0020370 PE=3 SV=1</td></tr>
<tr><td>11</td><td>>tr|E7D6B9|E7D6B9 9PEZI Cellobiose dehydrogenase OS=Crassicarpon thermophilum OX=225584 GN=cdhIIB PE=2 SV=1</td></tr>
<tr><td>12</td><td>>tr|B2ADY6|B2ADY6 PODAN Podospora anserina S mat+ genomic DNA chromosome 4 supercontig 2 OS=Podospora anserina (strain S / ATCC MYA-4624 / DSM 980 / FGSC 10383) OX=515849 GN=PODANS 0</td></tr>
<tr><td>13</td><td>>tr|A0A447C8C2|A0A447C8C2 PODCO Cellobiose dehydrogenase OS=Podospora comata OX=48703 GN=PODCO 000280 PE=3 SV=1</td></tr>
<tr><td>14</td><td>>tr|A0A420YBW0|A0A420YBW0 9PEZI CBM1 domain-containing protein OS=Coniochaeta pulveracea OX=177199 GN=DL546 008237 PE=3 SV=1</td></tr>
<tr><td>15</td><td>>tr|A0A1B8GV91|A0A1B8GV91 9PEZI Uncharacterized protein OS=Pseudogymnoascus verrucosus OX=342668 GN=VE01 02026 PE=3 SV=1</td></tr>
<tr><td>16</td><td>>tr|A0A1B8DQI5|A0A1B8DQI5 9PEZI GMC OxRdtase N domain-containing protein OS=Pseudogymnoascus sp. 23342-1-I1 OX=1524831 GN=VE03 09246 PE=3 SV=1</td></tr>
<tr><td>17</td><td>>tr|A0A1B8CBR7|A0A1B8CBR7 9PEZI Uncharacterized protein OS=Pseudogymnoascus sp. WSF 3629 OX=1622147 GN=VE00 05478 PE=3 SV=1</td></tr>
<tr><td>18</td><td>>tr|A0A175WAT0|A0A175WAT0 9PEZI Cellobiose dehydrogenase OS=Madurella mycetomatis OX=100816 GN=MMYC01 204661 PE=3 SV=1</td></tr>
<tr><td>19</td><td>>tr|A0A0C3D776|A0A0C3D776 9PEZI Cellobiose dehydrogenase protein OS=Oidiodendron maius Zn OX=913774 GN=OIDMADRAFT 105259 PE=3 SV=1</td></tr>
<tr><td>20</td><td>>tr|A0A094JIY5|A0A094JIY5 9PEZI Uncharacterized protein OS=Pseudogymnoascus sp. VKM F-4520 (FW-2644) OX=1420915 GN=V502 08998 PE=3 SV=1</td></tr>
<tr><td>21</td><td>>tr|A0A094HRR1|A0A094HRR1 9PEZI Uncharacterized protein OS=Pseudogymnoascus sp. VKM F-4519 (FW-2642) OX=1420914 GN=V501 04977 PE=3 SV=1</td></tr>
<tr><td>22</td><td>>tr|A0A094GTF7|A0A094GTF7 9PEZI GMC OxRdtase N domain-containing protein OS=Pseudogymnoascus sp. VKM F-4517 (FW-2822) OX=1420911 GN=V498 00806 PE=3 SV=1</td></tr>
<tr><td>23</td><td>>tr|A0A094AZS3|A0A094AZS3 9PEZI GMC OxRdtase N domain-containing protein OS=Pseudogymnoascus sp. VKM F-4513 (FW-928) OX=1420907 GN=V494 06391 PE=3 SV=1</td></tr>
<tr><td>24</td><td>>tr|A0A093ZEQ8|A0A093ZEQ8 9PEZI GMC OxRdtase N domain-containing protein OS=Pseudogymnoascus sp. VKM F-3775 OX=1420901 GN=V491 08113 PE=3 SV=1</td></tr>
<tr><td>25</td><td>>tr|A0A093XV99|A0A093XV99 9PEZI GMC OxRdtase N domain-containing protein OS=Pseudogymnoascus sp. VKM F-3557 OX=1437433 GN=V490 04347 PE=3 SV=1</td></tr>
<tr><td>26</td><td>>KAH6842501.1 cellobiose dehydrogenase Chaetomium sp. MPI-CAGE-AT-0009</td></tr>
<tr><td>27</td><td>>KAH6617338.1 cellobiose dehydrogenase Chaetomium globosum</td></tr>
<tr><td>28</td><td>>KAH6613732.1 cellobiose dehydrogenase Chaetomium sp. MPI-SDFR-AT-0129</td></tr>
<tr><td>29</td><td>>KAG7286306.1 hypothetical protein NEMBOFW57 008614 Staphylotrichum longicolle</td></tr>
<tr><td>30</td><td>>XP 038730079.1 uncharacterized protein EAE97 008482 Botrytis byssoidea</td></tr>
<tr><td>31</td><td>>XP 037189695.1 putative cellobiose dehydrogenase protein Botrytis fragariae</td></tr>
<tr><td>32</td><td>>XP 031871243.1 FAD protein Venustampulla echinocandica</td></tr>
<tr><td>33</td><td>>TVY81093.1 Cellobiose dehydrogenase Lachnellula suecica</td></tr>
<tr><td>34</td><td>>tr|W9CI51|W9CI51 SCLBF Carbohydrate-Binding Module family 1 OS=Sclerotinia borealis (strain F-4128) OX=1432307 GN=SBOR 5272 PE=3 SV=1</td></tr>
<tr><td>35</td><td>>tr|U7Q3V9|U7Q3V9 SPOS1 GMC OxRdtase N domain-containing protein OS=Sporothrix schenckii (strain ATCC 58251 / de Perez 2211183) OX=1391915 GN=HMPREF1624 00009 PE=3 SV=1</td></tr>
<tr><td>36</td><td>>tr|R8BXS0|R8BXS0 TOGMI Putative cellobiose dehydrogenase protein OS=Togninia minima (strain UCR-PA7) OX=1286976 GN=UCRPA7 378 PE=3 SV=1</td></tr>
<tr><td>37</td><td>>tr|Q7RXM0|Q7RXM0 NEUCR Cellobiose dehydrogenase OS=Neurospora crassa (strain ATCC 24698 / 74-OR23-1A / CBS 708.71 / DSM 1257 / FGSC 987) OX=367110 GN=cdh-1 PE=1 SV=1</td></tr>
<tr><td>38</td><td>>tr|Q2H8S4|Q2H8S4 CHAGB CBM1 domain-containing protein OS=Chaetomium globosum (strain ATCC 6205 / CBS 148.51 / DSM 1962 / NBRC 6347 / NRRL 1970) OX=306901 GN=CHGG 03380 PE=3 SV=1</td></tr>
<tr><td>39</td><td>>tr|O74240|O74240 MYCTT Carbohydrate-binding module family 1 protein OS=Myceliophthora thermophila (strain ATCC 42464 / BCRC 31852 / DSM 1799) OX=573729 GN=cdh PE=2 SV=1</td></tr>
<tr><td>40</td><td>>tr|M7UYV4|M7UYV4 BOTF1 Putative cellobiose dehydrogenase protein OS=Botryotinia fuckeliana (strain BcDW1) OX=1290391 GN=BcDW1 2363 PE=3 SV=1</td></tr>
<tr><td>41</td><td>>tr|J3P7F1|J3P7F1 GAET3 CBM1 domain-containing protein OS=Gaeumannomyces tritici (strain R3-111a-1) OX=644352 GN=20349900 PE=3 SV=1</td></tr>
<tr><td>42</td><td>>tr|H0ECJ1|H0ECJ1 GLAL7 Putative Cellobiose dehydrogenase OS=Glarea lozoyensis (strain ATCC 74030 / MF5533) OX=1104152 GN=M7I 0131 PE=3 SV=1</td></tr>
<tr><td>43</td><td>>tr|G4UQF4|G4UQF4 NEUT9 CBM1 domain-containing protein OS=Neurospora tetrasperma (strain FGSC 2509 / P0656) OX=510952 GN=NEUTE2DRAFT 150403 PE=3 SV=1</td></tr>
<tr><td>44</td><td>>tr|G2RB43|G2RB43 THETT Carbohydrate-binding module family 1 protein OS=Thermothielavioides terrestris (strain ATCC 38088 / NRRL 8126) OX=578455 GN=THITE 59724 PE=3 SV=1</td></tr>
<tr><td>45</td><td>>tr|G0S434|G0S434 CHATD Cellobiose dehydrogenase-like protein OS=Chaetomium thermophilum (strain DSM 1495 / CBS 144.50 / IMI 039719) OX=759272 GN=CTHT 0022390 PE=3 SV=1</td></tr>
<tr><td>46</td><td>>tr|F7W2Z9|F7W2Z9 SORMK WGS project CABT00000000 data contig 2.23 OS=Sordaria macrospora (strain ATCC MYA-333 / DSM 997 / K(L3346) / K-hell) OX=771870 GN=SMAC 02223 PE=3 SV=1</td></tr>
<tr><td>47</td><td>>tr|E7D6C0|E7D6C0 9PEZI Cellobiose dehydrogenase OS=Amesia atrobrunnea OX=1934350 GN=cdhIIA PE=2 SV=1</td></tr>
<tr><td>48</td><td>>tr|A9XK88|A9XK88 9PEZI Cellobiose dehydrogenase OS=Crassicarpon hotsonii OX=455373 GN=CDH PE=1 SV=1</td></tr>
<tr><td>49</td><td>>tr|A0A8A3PCS0|A0A8A3PCS0 9HELO Uncharacterized protein OS=Monilinia vaccinii-corymbosi OX=61207 GN=DSL72 002481 PE=4 SV=1</td></tr>
<tr><td>50</td><td>>tr|A0A7J6JAZ4|A0A7J6JAZ4 COLFN Cellobiose dehydrogenase OS=Colletotrichum fructicola (strain Nara gc5) OX=1213859 GN=CDH-1-5 PE=3 SV=1</td></tr>
<tr><td>51</td><td>>tr|A0A5N6U7V6|A0A5N6U7V6 9EURO Uncharacterized protein OS=Aspergillus avenaceus OX=36643 GN=BDV25DRAFT 147246 PE=3 SV=1</td></tr>
<tr><td>52</td><td>>tr|A0A5N6JUK3|A0A5N6JUK3 9HELO CBM1 domain-containing protein OS=Monilinia laxa OX=61186 GN=EYC80 007861 PE=3 SV=1</td></tr>
<tr><td>53</td><td>>tr|A0A5N5JWI6|A0A5N5JWI6 9PEZI Carbohydrate-binding module family 1 protein OS=Coniochaeta sp. 2T2.1 OX=1571157 GN=GE09DRAFT 487357 PE=3 SV=1</td></tr>
<tr><td>54</td><td>>tr|A0A5N5JP91|A0A5N5JP91 9PEZI Carbohydrate-binding module family 1 protein OS=Coniochaeta sp. 2T2.1 OX=1571157 GN=GE09DRAFT 573962 PE=3 SV=1</td></tr>
<tr><td>55</td><td>>tr|A0A507AMV8|A0A507AMV8 9PEZI CBM1 domain-containing protein OS=Phialemoniopsis curvata OX=1093900 GN=E0L32 007180 PE=3 SV=1</td></tr>
<tr><td>56</td><td>>tr|A0A4Z1KAQ1|A0A4Z1KAQ1 9HELO CBM1 domain-containing protein OS=Botrytis porri OX=87229 GN=BPOR 0688g00040 PE=3 SV=1</td></tr>
<tr><td>57</td><td>>tr|A0A4Z1IDK4|A0A4Z1IDK4 9HELO CBM1 domain-containing protein OS=Botryotinia narcissicola OX=278944 GN=BOTNAR 0158g00030 PE=3 SV=1</td></tr>
<tr><td>58</td><td>>tr|A0A4Y8DC68|A0A4Y8DC68 9HELO CBM1 domain-containing protein OS=Botryotinia calthae OX=38488 GN=BOTCAL 0062g00170 PE=3 SV=1</td></tr>
<tr><td>59</td><td>>tr|A0A447CK33|A0A447CK33 PODCO Cellobiose dehydrogenase OS=Podospora comata OX=48703 GN=PODCO 702650 PE=3 SV=1</td></tr>
<tr><td>60</td><td>>tr|A0A2S7QDX6|A0A2S7QDX6 9HELO Cellobiose dehydrogenase protein OS=Rutstroemia sp. NJR-2017a WRK4 OX=2070412 GN=CJF32 00007006 PE=3 SV=1</td></tr>
<tr><td>61</td><td>>tr|A0A2S7QC00|A0A2S7QC00 9HELO Cellobiose dehydrogenase protein OS=Rutstroemia sp. NJR-2017a BVV2 OX=2070413 GN=CJF31 00002280 PE=3 SV=1</td></tr>
<tr><td>62</td><td>>tr|A0A2J6S845|A0A2J6S845 9HELO Cellobiose dehydrogenase protein OS=Hyaloscypha variabilis F OX=1149755 GN=L207DRAFT 418430 PE=3 SV=1</td></tr>
<tr><td>63</td><td>>tr|A0A2I7VT52|A0A2I7VT52 9PEZI Cellobiose dehydrogenase OS=Coniochaeta hoffmannii OX=91930 PE=3 SV=1</td></tr>
<tr><td>64</td><td>>tr|A0A1L7XLG2|A0A1L7XLG2 9HELO Probable cellobiose dehydrogenase OS=Phialocephala subalpina OX=576137 GN=PAC 15677 PE=3 SV=1</td></tr>
<tr><td>65</td><td>>tr|A0A1L7X1V4|A0A1L7X1V4 9HELO Probable cellobiose dehydrogenase OS=Phialocephala subalpina OX=576137 GN=PAC 08841 PE=3 SV=1</td></tr>
<tr><td>66</td><td>>tr|A0A1J7IZ49|A0A1J7IZ49 9PEZI Cellobiose dehydrogenase OS=Coniochaeta ligniaria NRRL 30616 OX=1408157 GN=CONLIGDRAFT 161351 PE=3 SV=1</td></tr>
<tr><td>67</td><td>>tr|A0A1D9QEZ0|A0A1D9QEZ0 SCLS1 CBM1 domain-containing protein OS=Sclerotinia sclerotiorum (strain ATCC 18683 / 1980 / Ss-1) OX=665079 GN=sscle 11g082770 PE=3 SV=1</td></tr>
<tr><td>68</td><td>>tr|A0A194XK54|A0A194XK54 9HELO Putative cellobiose dehydrogenase OS=Mollisia scopiformis OX=149040 GN=LY89DRAFT 780009 PE=3 SV=1</td></tr>
<tr><td>69</td><td>>tr|A0A194WUG2|A0A194WUG2 9HELO FAD/NAD(P)-binding domain-containing protein OS=Mollisia scopiformis OX=149040 GN=LY89DRAFT 739447 PE=3 SV=1</td></tr>
<tr><td>70</td><td>>tr|A0A0C4DTQ5|A0A0C4DTQ5 MAGP6 CBM1 domain-containing protein OS=Magnaporthiopsis poae (strain ATCC 64411 / 73-15) OX=644358 GN=MAPG 03327 PE=3 SV=1</td></tr>
<tr><td>71</td><td>>TAQ89554.1 cellobiose dehydrogenase Chlorociboria aeruginascens</td></tr>
<tr><td>72</td><td>>KAH8902580.1 cellobiose dehydrogenase Coniochaeta sp. PMI 546</td></tr>
<tr><td>73</td><td>>KAH8879114.1 carbohydrate-binding module family 1 protein Thozetella sp. PMI 491</td></tr>
<tr><td>74</td><td>>KAH6849701.1 hypothetical protein B0I37DRAFT 94521 Chaetomium sp. MPI-CAGE-AT-0009</td></tr>
<tr><td>75</td><td>>KAH6624220.1 cellobiose dehydrogenase Chaetomium sp. MPI-SDFR-AT-0129</td></tr>
<tr><td>76</td><td>>KAG9229698.1 putative cellobiose dehydrogenase Amylocarpus encephaloides</td></tr>
<tr><td>77</td><td>>KAG7291000.1 hypothetical protein NEMBOFW57 001008 Staphylotrichum longicolle</td></tr>
<tr><td>78</td><td>>KAF8856767.1 carbohydrate-binding module family 1 Acephala macrosclerotiorum</td></tr>
<tr><td>79</td><td>>KAF7890600.1 hypothetical protein EAF00 008915 Botryotinia globosa</td></tr>
<tr><td>80</td><td>>KAF7854740.1 hypothetical protein EAF04 010308 Sclerotium cepivorum</td></tr>
<tr><td>81</td><td>>KAE9362713.1 hypothetical protein N431DRAFT 424556 Chalara longipes BDJ</td></tr>
<tr><td>82</td><td>>KAE8447572.1 hypothetical protein EG329 010702 Helotiales sp. DMI Dod QoI</td></tr>
<tr><td>83</td><td>>KAE8445026.1 hypothetical protein EG329 014032 Helotiales sp. DMI Dod QoI</td></tr>
<tr><td>84</td><td>>CAG8980480.1 hypothetical protein HYALB 00013116 Hymenoscyphus albidus</td></tr>
<tr><td>85</td><td>>CAG8961887.1 hypothetical protein HYFRA 00013687 partial Hymenoscyphus fraxineus</td></tr>
<tr><td>86</td><td>>CAD6451742.1 50cf6ae4-cc67-4403-9e85-a490e002ba7e Sclerotinia trifoliorum</td></tr>
<tr><td>87</td><td>>XP 043160197.1 uncharacterized protein Asppvi 008392 Aspergillus pseudoviridinutans</td></tr>
<tr><td>88</td><td>>XP 043121133.1 uncharacterized protein Aspvir 000053 Aspergillus viridinutans</td></tr>
<tr><td>89</td><td>>XP 033418655.1 cellobiose dehydrogenase Aspergillus lentulus</td></tr>
<tr><td>90</td><td>>XP 024688248.1 cellobiose dehydrogenase Aspergillus novofumigatus IBT 16806</td></tr>
<tr><td>91</td><td>>tr|Q4WZA6|Q4WZA6 ASPFU Cellobiose dehydrogenase OS=Neosartorya fumigata (strain ATCC MYA-4609 / Af293 / CBS 101355 / FGSC A1100) OX=330879 GN=AFUA 2G17620 PE=3 SV=1</td></tr>
<tr><td>92</td><td>>tr|A1DIY3|A1DIY3 NEOFI Cellobiose dehydrogenase OS=Neosartorya fischeri (strain ATCC 1020 / DSM 3700 / CBS 544.65 / FGSC A1164 / JCM 1740 / NRRL 181 / WB 181) OX=331117 GN=NFIA 093020 PE=</td></tr>
<tr><td>93</td><td>>tr|A0A7H8RC44|A0A7H8RC44 9EURO CBM1 domain-containing protein OS=Talaromyces rugulosus OX=121627 GN=TRUGW13939 09239 PE=3 SV=1</td></tr>
</table>

94	>tr|A0A5N7CNR2|A0A5N7CNR2 PETAA GMC OxRdtase N domain-containing protein OS=Petromyces alliaceus OX=209559 GN=BDV23DRAFT 168574 PE=3 SV=1
95	>tr|A0A5N6GAW0|A0A5N6GAW0 PETAA GMC OxRdtase N domain-containing protein OS=Petromyces alliaceus OX=209559 GN=BDW43DRAFT 305991 PE=3 SV=1
96	>tr|A0A3R7J6G8|A0A3R7J6G8 9EURO Uncharacterized protein OS=Aspergillus turcosus OX=1245748 GN=CFD26 105824 PE=3 SV=1
97	>tr|A0A397ICW3|A0A397ICW3 9EURO Uncharacterized protein OS=Aspergillus turcosus OX=1245748 GN=CDV55 108154 PE=3 SV=1
98	>tr|A0A397HPG6|A0A397HPG6 9EURO Uncharacterized protein OS=Aspergillus thermomutatus OX=41047 GN=CDV56 105354 PE=3 SV=1
99	>tr|A0A2T2P9Q4|A0A2T2P9Q4 CORCC Cellobiose dehydrogenase-like protein OS=Corynespora cassiicola Philippines OX=1448308 GN=BS50DRAFT 567230 PE=3 SV=1
100	>tr|A0A1B8E8L3|A0A1B8E8L3 9PEZI CBM20 domain-containing protein OS=Pseudogymnoascus sp. 23342-1-I1 OX=1524831 GN=VE03 03484 PE=3 SV=1
101	>tr|A0A0U1LME6|A0A0U1LME6 TALIS Cellobiose dehydrogenase OS=Talaromyces islandicus OX=28573 GN=PISL3812 00950 PE=3 SV=1
102	>tr|A0A0K8LHF9|A0A0K8LHF9 9EURO Cellobiose dehydrogenase OS=Aspergillus udagawae OX=91492 GN=AUD 6602 PE=3 SV=1
103	>tr|A0A0B7KNB7|A0A0B7KNB7 BIOOC CBM1 domain-containing protein OS=Bionectria ochroleuca OX=29856 GN=BN869 000012438 1 PE=3 SV=1
104	>tr|A0A094A320|A0A094A320 9PEZI CBM20 domain-containing protein OS=Pseudogymnoascus sp. VKM F-3775 OX=1420901 GN=V491 04962 PE=3 SV=1
105	>KAH8705730.1 cellobiose dehydrogenase Talaromyces proteolyticus
106	>KAH3192251.1 hypothetical protein KXW62 006330 Aspergillus fumigatus
107	>KAH2982227.1 hypothetical protein KXW58 001320 Aspergillus fumigatus
108	>KAG2003843.1 hypothetical protein GB937 009335 Aspergillus fischeri
109	>KAF7165034.1 hypothetical protein CNMCM5623 009386 Aspergillus felis
110	>KAF7117491.1 hypothetical protein CNMCM5793 006473 Aspergillus hiratsukae
111	>GFF44357.1 cellobiose dehydrogenase Aspergillus udagawae
112	>XP 031937681.1 uncharacterized protein BDV37DRAFT 286730 Aspergillus pseudonomiae
113	>XP 029754270.1 hypothetical protein PpBr36 03722 Pyricularia pennisetigena
114	>XP 024706630.1 cellobiose dehydrogenase Aspergillus steynii IBT 23096
115	>XP 022387007.1 cellobiose dehydrogenase Aspergillus bombycis
116	>TVY84745.1 Cellobiose dehydrogenase Lachnellula suecica
117	>tr|Q0C8J1|Q0C8J1 ASPTN GMC OxRdtase N domain-containing protein OS=Aspergillus terreus (strain NIH 2624 / FGSC A1156) OX=341663 GN=ATEG 09993 PE=3 SV=1
118	>tr|L7IQ31|L7IQ31 MAGOY Cellobiose dehydrogenase OS=Magnaporthe oryzae (strain Y34) OX=1143189 GN=OOU Y34scaffold00033g56 PE=3 SV=1
119	>tr|I8IAU4|I8IAU4 ASPO3 Cellobiose dehydrogenase OS=Aspergillus oryzae (strain 3.042) OX=1160506 GN=Ao3042 09464 PE=3 SV=1
120	>tr|H0ERH5|H0ERH5 GLAL7 Putative Cellobiose dehydrogenase OS=Glarea lozoyensis (strain ATCC 74030 / MF5533) OX=1104152 GN=M7I 5294 PE=3 SV=1
121	>tr|A1C890|A1C890 ASPCL Cellobiose dehydrogenase OS=Aspergillus clavatus (strain ATCC 1007 / CBS 513.65 / DSM 816 / NCTC 3887 / NRRL 1 / QM 1276 / 107) OX=344612 GN=ACLA 076510 PE=3 SV=1
122	>tr|A0A6P8ARA2|A0A6P8ARA2 MAGGR uncharacterized protein OS=Magnaporthe grisea OX=148305 GN=PgNI 09374 PE=3 SV=1
123	>tr|A0A5N7B622|A0A5N7B622 9EURO Uncharacterized protein OS=Aspergillus bertholletiae OX=1226010 GN=BDV26DRAFT 293039 PE=3 SV=1
124	>tr|A0A5N6ZCU3|A0A5N6ZCU3 9EURO Uncharacterized protein OS=Aspergillus coremiiformis OX=138285 GN=BDV28DRAFT 146138 PE=3 SV=1
125	>tr|A0A5N5X0I7|A0A5N5X0I7 9EURO GMC OxRdtase N domain-containing protein OS=Aspergillus leporis OX=41062 GN=BDV29DRAFT 173896 PE=3 SV=1
126	>tr|A0A5M3ZDT3|A0A5M3ZDT3 ASPTE GMC OxRdtase N domain-containing protein OS=Aspergillus terreus OX=33178 GN=ATETN484 0016003100 PE=3 SV=1
127	>tr|A0A3D8RIX2|A0A3D8RIX2 9HELO Cellobiose dehydrogenase protein OS=Coleophoma crateriformis OX=565419 GN=BP5796 07362 PE=3 SV=1
128	>tr|A0A2B7YXP2|A0A2B7YXP2 9EURO Uncharacterized protein OS=Polytolypa hystricis UAMH7299 OX=1447883 GN=AJ80 01924 PE=3 SV=1
129	>tr|A0A1Q8RZF0|A0A1Q8RZF0 9PEZI Cellobiose dehydrogenase 6 OS=Colletotrichum chlorophyti OX=708187 GN=CCHL11 07523 PE=3 SV=1
130	>tr|A0A0L1JHS4|A0A0L1JHS4 ASPNO Cellobiose dehydrogenase OS=Aspergillus nomiae NRRL 13137 OX=1509407 GN=ANOM 000372 PE=3 SV=1
131	>TLD31514.1 hypothetical protein PspLS 01974 Pyricularia sp. CBS 133598
132	>RAQ52747.1 cellobiose dehydrogenase Aspergillus flavus
133	>KAH8815586.1 hypothetical protein F5884DRAFT 183280 Xylogone sp. PMI 703
134	>KAH8586285.1 hypothetical protein B0O99DRAFT 74929 Bisporella sp. PMI 857
135	>KAH8429338.1 hypothetical protein LDX57 007005 Aspergillus melleus
136	>KAH8158440.1 hypothetical protein CIB48 g9809 Xylaria polymorpha
137	>KAG2421927.1 hypothetical protein HFD88 005903 Aspergillus terreus
138	>KAF7586668.1 hypothetical protein BBP40 008517 Aspergillus hancockii
139	>KAF4624197.1 hypothetical protein G7Y89 g13978 Cudoniella acicularis
140	>KAF2090894.1 hypothetical protein K490DRAFT 71198 Saccharata proteae CBS 121410
141	>XP 031003404.1 Cellobiose dehydrogenase Lachnellula hyalina
142	>TVY48802.1 Cellobiose dehydrogenase Lachnellula occidentalis
143	>TVY40768.1 Cellobiose dehydrogenase partial Lachnellula subtilissima
144	>TVY18546.1 Cellobiose dehydrogenase Lachnellula arida
145	>tr|K1WT04|K1WT04 MARBU Fungal cellulose binding domain-containing protein OS=Marssonina brunnea f. sp. multigermtubi (strain MB m1) OX=1072389 GN=MBM 01462 PE=3 SV=1
146	>tr|A0A6A5VZH0|A0A6A5VZH0 9PLEO Uncharacterized protein OS=Amniculicola lignicola CBS 123094 OX=1392246 GN=P154DRAFT 475260 PE=3 SV=1
147	>tr|A0A3E2HIL5|A0A3E2HIL5 SCYLI CBM1 domain-containing protein (Fragment) OS=Scytalidium lignicola OX=5539 GN=B7463 g3098 PE=3 SV=1
148	>tr|A0A1Y2WHE3|A0A1Y2WHE3 9PEZI Carbohydrate-binding module family 1 protein OS=Hypoxylon sp. CI-4A OX=1001833 GN=M426DRAFT 7107 PE=3 SV=1
149	>tr|A0A1Y2VGD9|A0A1Y2VGD9 9PEZI Cellobiose dehydrogenase protein OS=Hypoxylon sp. CO27-5 OX=1001938 GN=M434DRAFT 118226 PE=3 SV=1
150	>KAH8674540.1 putative cellobiose dehydrogenase Hymenoscyphus varicosporioides
151	>KAH6670475.1 hypothetical protein B0J14DRAFT 97535 Halenospora varia
152	>KAF2841371.1 carbohydrate-binding module family 1 protein Patellaria atrata CBS 101060
153	>XP 044698261.1 uncharacterized protein H6S33 009446 Morchella sextelata
154	>XP 040665172.1 uncharacterized protein ASPVEDRAFT 882784 Aspergillus versicolor CBS 583.65
155	>XP 033654009.1 cellobiose dehydrogenase-like protein Westerdykella ornata
156	>XP 033396073.1 carbohydrate-binding module family 1 protein Aplosporella prunicola CBS 121167
157	>tr|A0A6A6EID0|A0A6A6EID0 9PEZI GMC OxRdtase N domain-containing protein OS=Zopfia rhizophila CBS 207.26 OX=1314779 GN=K469DRAFT 718852 PE=3 SV=1
158	>tr|A0A3N4KIA5|A0A3N4KIA5 9PEZI CBM1 domain-containing protein OS=Morchella conica CCBAS932 OX=1392247 GN=P167DRAFT 559771 PE=3 SV=1
159	>KAH8680545.1 cellobiose dehydrogenase Xylariales sp. PMI 506
160	>KAH7349010.1 cellobiose dehydrogenase-like protein Pyrenochaeta sp. MPI-SDFR-AT-0127
161	>KAG8629314.1 hypothetical protein KVT40 003179 Elsinoe batatas
162	>KAF8470891.1 cellobiose dehydrogenase Kalaharituber pfeilii
163	>KAF8453744.1 cellobiose dehydrogenase Kalaharituber pfeilii
164	>KAF2225283.1 cellobiose dehydrogenase Elsinoe ampelina
165	>KAF1962546.1 cellobiose dehydrogenase-like protein Byssothecium circinans
166	>CAG8976289.1 hypothetical protein HYALB 00011774 Hymenoscyphus albidus
167	>CAG8953832.1 hypothetical protein HYFRA 00006724 Hymenoscyphus fraxineus
168	>XP 041551944.1 uncharacterized protein APUU 20182S Aspergillus puulaauensis
169	>XP 040748598.1 uncharacterized protein P175DRAFT 0519267 Aspergillus ochraceoroseus IBT 24754
170	>XP 033434409.1 Cellobiose dehydrogenase Daldinia childiae
171	>tr|W7EMQ5|W7EMQ5 COCVI Carbohydrate-binding module family 1 protein OS=Bipolaris victoriae FI3 OX=930091 GN=COCVIDRAFT 24813 PE=3 SV=1
172	>tr|W6ZHW6|W6ZHW6 COCMI Carbohydrate-binding module family 1 protein OS=Bipolaris oryzae ATCC 44560 OX=930090 GN=COCMIDRAFT 91230 PE=3 SV=1
173	>tr|N4X498|N4X498 COCH4 Carbohydrate-binding module family 1 protein OS=Cochliobolus heterostrophus (strain C4 / ATCC 48331 / race T) OX=665024 GN=COCC4DRAFT 188966 PE=3 SV=1
174	>tr|E7D6C3|E7D6C3 9PEZI Cellobiose dehydrogenase OS=Hypoxylon haematostroma OX=152305 GN=cdhIIB PE=2 SV=1
175	>tr|A0A6A6V5A8|A0A6A6V5A8 9PLEO Uncharacterized protein OS=Sporormia fimetaria CBS 119925 OX=1340428 GN=M011DRAFT 428071 PE=3 SV=1
176	>tr|A0A6A6SG76|A0A6A6SG76 9PLEO Uncharacterized protein OS=Massarina eburnea CBS 473.64 OX=1395130 GN=P280DRAFT 465050 PE=3 SV=1
177	>tr|A0A1Y2X538|A0A1Y2X538 9PEZI Cellobiose dehydrogenase protein OS=Daldinia sp. EC12 OX=1001832 GN=K445DRAFT 58811 PE=3 SV=1
178	>tr|A0A1Y2LSU9|A0A1Y2LSU9 EPING Uncharacterized protein OS=Epicoccum nigrum OX=105696 GN=B5807 08920 PE=3 SV=1
179	>tr|A0A1W2TAW4|A0A1W2TAW4 ROSNE Putative cellobiose dehydrogenase OS=Rosellinia necatrix OX=77044 GN=SAMD00023353 0800710 PE=3 SV=1
180	>tr|A0A1S7UNL2|A0A1S7UNL2 ROSNE Putative cellobiose dehydrogenase OS=Rosellinia necatrix OX=77044 GN=SAMD00023353 1101800 PE=3 SV=1
181	>tr|A0A136IN00|A0A136IN00 9PEZI CBM1 domain-containing protein OS=Microdochium bolleyi OX=196109 GN=Micbo1qcDRAFT 209370 PE=3 SV=1
182	>tr|A0A0U5CF15|A0A0U5CF15 ASPCI Putative Cellobiose dehydrogenase (AFU orthologue AFUA 2G17620) OS=Aspergillus calidoustus OX=454130 GN=ASPCAL11875 PE=3 SV=1
183	>tr|A0A0B7K5V7|A0A0B7K5V7 BIOOC GMC OxRdtase N domain-containing protein OS=Bionectria ochroleuca OX=29856 GN=BN869 000006995 1 PE=3 SV=1
184	>KAH9214538.1 putative cellobiose dehydrogenase Leptodontidium sp. 2 PMI 412
185	>KAH8167374.1 cellobiose dehydrogenase Xylaria polymorpha
186	>KAH7398017.1 hypothetical protein BKA64DRAFT 55941 Leotiomycetes sp. MPI-SDFR-AT-0126
187	>KAH7354699.1 hypothetical protein BKA65DRAFT 227085 Rhexocercosporidium sp. MPI-PUGE-AT-0058
188	>KAH7079446.1 hypothetical protein FB567DRAFT 532592 Paraphoma chrysanthemicola
189	>KAH7071222.1 hypothetical protein BKA63DRAFT 474784 Paraphoma chrysanthemicola
190	>KAH7031437.1 cellobiose dehydrogenase Microdochium trichocladiopsis
191	>KAG9199242.1 hypothetical protein G6514 008685 Epicoccum nigrum
192	>KAG4435454.1 hypothetical protein IFR05 009056 Cadophora sp. M221
193	>KAG4414375.1 hypothetical protein IFR04 012512 Cadophora malorum
194	>KAF8425211.1 cellobiose dehydrogenase Tirmania nivea
195	>KAF2966942.1 cellobiose dehydrogenase Xylaria multiplex

196	>CAH0056250.1 unnamed protein product Clonostachys solani
197	>CAH0047161.1 unnamed protein product Clonostachys solani
198	>XP 040709064.1 uncharacterized protein ASPSYDRAFT 378541 Aspergillus sydowii CBS 593.65
199	>XP 033542074.1 cellobiose dehydrogenase-like protein Lindgomyces ingoldianus
200	>tr|U4LD08|U4LD08 PYROM Similar to Cellobiose dehydrogenase acc. no. Q01738 OS=Pyronema omphalodes (strain CBS 100304) OX=1076935 GN=PCON 04062 PE=3 SV=1
201	>tr|Q0UAG6|Q0UAG6 PHANO CBM1 domain-containing protein OS=Phaeosphaeria nodorum (strain SN15 / ATCC MYA-4574 / FGSC 10173) OX=321614 GN=JI435 112480 PE=3 SV=2
202	>tr|M2TCT3|M2TCT3 COCSN Carbohydrate-binding module family 1 protein OS=Cochliobolus sativus (strain ND90Pr / ATCC 201652) OX=665912 GN=COCSADRAFT 196954 PE=3 SV=1
203	>tr|A0A6G1JTC1|A0A6G1JTC1 9PLEO GMC OxRdtase N domain-containing protein OS=Pleomassaria siparia CBS 279.74 OX=1314801 GN=K504DRAFT 418418 PE=3 SV=1
204	>tr|A0A6G1J5D1|A0A6G1J5D1 9PLEO Uncharacterized protein OS=Lentithecium fluviatile CBS 122367 OX=1168545 GN=K458DRAFT 337564 PE=3 SV=1
205	>tr|A0A553IDU3|A0A553IDU3 9PEZI CBM1 domain-containing protein OS=Xylaria flabelliformis OX=2512241 GN=FHL15 001009 PE=3 SV=1
206	>tr|A0A439DGR5|A0A439DGR5 9PEZI GMC OxRdtase N domain-containing protein OS=Xylaria grammica OX=363999 GN=EKO27 g1481 PE=3 SV=1
207	>tr|A0A2P7Z6E5|A0A2P7Z6E5 9PEZI Cellobiose dehydrogenase OS=Elsinoe australis OX=40998 GN=B9Z65 7283 PE=3 SV=1
208	>tr|A0A2K1QSJ6|A0A2K1QSJ6 9PEZI Cellobiose dehydrogenase OS=Sphaceloma murrayae OX=2082308 GN=CAC42 2 PE=3 SV=1
209	>tr|A0A1B8GJG1|A0A1B8GJG1 9PEZI GMC OxRdtase N domain-containing protein OS=Pseudogymnoascus verrucosus OX=342668 GN=VE01 06712 PE=3 SV=1
210	>tr|A0A1B8CYH6|A0A1B8CYH6 9PEZI CBM1 domain-containing protein OS=Pseudogymnoascus sp. 24MN13 OX=1622150 GN=VE04 07999 PE=3 SV=1
211	>tr|A0A094DZ68|A0A094DZ68 9PEZI GMC OxRdtase N domain-containing protein OS=Pseudogymnoascus sp. VKM F-103 OX=1420912 GN=V499 08183 PE=3 SV=1
212	>tr|A0A086T046|A0A086T046 ACRC1 Cellobiose dehydrogenase-like protein OS=Acremonium chrysogenum (strain ATCC 11550 / CBS 779.69 / DSM 880 / IAM 14645 / JCM 23072 / IMI 49137) OX=857340 GN=
213	>RYC65950.1 cellobiose dehydrogenase Xylaria longipes
214	>KAH8731998.1 hypothetical protein GQ44DRAFT 642000 Phaeosphaeriaceae sp. PMI808
215	>KAH8672005.1 putative cellobiose dehydrogenase Hymenoscyphus varicosporioides
216	>KAH7135211.1 hypothetical protein B0J11DRAFT 155028 Dendryphion nanum
217	>KAF4631847.1 cellobiose dehydrogenase Cudoniella acicularis
218	>KAF4549314.1 Dehydrogenase-like protein Elsinoe fawcettii
219	>KAF3009550.1 hypothetical protein E8E13 005488 Curvularia kusanoi
220	>KAF2971080.1 cellobiose dehydrogenase Xylaria multiplex
221	>KAF2448593.1 hypothetical protein P171DRAFT 518089 Karstenula rhodostoma CBS 690.94
222	>KAF2202979.1 cellobiose dehydrogenase-like protein Delitschia confertaspora ATCC 74209
223	>KAF1967448.1 cellobiose dehydrogenase-like protein Bimuria novae-zelandiae CBS 107.79
224	>KAA8893690.1 cellobiose dehydrogenase-like protein Sphaerosporella brunnea
225	>CAA9962915.1 Cellobiose dehydrogenase Pyrenophora teres f. maculata
226	>XP 043028729.1 uncharacterized protein INS49 006818 Diaporthe citri
227	>XP 035372866.1 Cellobiose dehydrogenase Lasiodiplodia theobromae
228	>XP 033519830.1 uncharacterized protein P153DRAFT 324742 Dothidotthia symphoricarpi CBS 119687
229	>XP 020129687.1 cellobiose dehydrogenase Diplodia corticola
230	>VUC25041.1 unnamed protein product Clonostachys rosea
231	>tr|A0A6A6XV31|A0A6A6XV31 9PLEO Uncharacterized protein OS=Melanomma pulvis-pyrius CBS 109.77 OX=1314802 GN=K505DRAFT 404062 PE=3 SV=1
232	>tr|A0A6A5QUY0|A0A6A5QUY0 AMPQU CBM1 domain-containing protein OS=Ampelomyces quisqualis OX=50730 GN=BDU57DRAFT 467912 PE=3 SV=1
233	>tr|A0A6A5KHL1|A0A6A5KHL1 9PLEO CBM1 domain-containing protein OS=Decorospora gaudefroyi OX=184978 GN=BDW02DRAFT 630778 PE=3 SV=1
234	>tr|A0A5N5DLB1|A0A5N5DLB1 9PEZI Cellobiose dehydrogenase OS=Lasiodiplodia theobromae OX=45133 GN=CDH-1 1 PE=3 SV=1
235	>tr|A0A4Z0ZAP4|A0A4Z0ZAP4 9PEZI Cellobiose dehydrogenase OS=Xylaria hypoxylon OX=37992 GN=E0Z10 g41 PE=3 SV=1
236	>tr|A0A4Z0YUR9|A0A4Z0YUR9 9PEZI CBM1 domain-containing protein OS=Xylaria hypoxylon OX=37992 GN=E0Z10 g5078 PE=3 SV=1
237	>tr|A0A3M7M1F9|A0A3M7M1F9 9PLEO Fungal cellulose binding domain-containing OS=Pyrenophora seminiperda CCB06 OX=1302712 GN=GMOD 00004457 PE=3 SV=1
238	>tr|A0A1V8UY55|A0A1V8UY55 9PEZI GMC OxRdtase N domain-containing protein OS=Rachicladosporium sp. CCFEE 5018 OX=1974281 GN=B0A51 04066 PE=3 SV=1
239	>tr|A0A1V8SL87|A0A1V8SL87 9PEZI GMC OxRdtase N domain-containing protein OS=Rachicladosporium antarcticum OX=1507870 GN=B0A48 14619 PE=3 SV=1
240	>tr|A0A1U8QW17|A0A1U8QW17 EMENI Uncharacterized protein OS=Emericella nidulans (strain FGSC A4 / ATCC 38163 / CBS 112.46 / NRRL 194 / M139) OX=227321 GN=AN7230.2 PE=3 SV=1
241	>tr|A0A1B8EMC4|A0A1B8EMC4 9PEZI GMC OxRdtase N domain-containing protein (Fragment) OS=Pseudogymnoascus sp. 05NY08 OX=1622149 GN=VF21 08540 PE=3 SV=1
242	>tr|A0A1B8CF93|A0A1B8CF93 9PEZI CBM1 domain-containing protein OS=Pseudogymnoascus sp. WSF 3629 OX=1622147 GN=VE00 05041 PE=3 SV=1
243	>tr|A0A162YD71|A0A162YD71 DIDRA Cellulose binding OS=Didymella rabiei OX=5454 GN=ST47 g8894 PE=3 SV=1
244	>tr|A0A094HQG1|A0A094HQG1 9PEZI GMC OxRdtase N domain-containing protein OS=Pseudogymnoascus sp. VKM F-4520 (FW-2644) OX=1420915 GN=V502 08083 PE=3 SV=1
245	>tr|A0A094CUU6|A0A094CUU6 9PEZI GMC OxRdtase N domain-containing protein OS=Pseudogymnoascus sp. VKM F-4516 (FW-969) OX=1420910 GN=V497 05278 PE=3 SV=1
246	>tr|A0A093ZG14|A0A093ZG14 9PEZI GMC OxRdtase N domain-containing protein (Fragment) OS=Pseudogymnoascus sp. VKM F-3775 OX=1420901 GN=V491 07867 PE=3 SV=1
247	>RYC59679.1 cellobiose dehydrogenase Xylaria longipes
248	>KAH6638985.1 hypothetical protein C7974DRAFT 353168 Boeremia exigua
249	>KAG9231179.1 hypothetical protein BJ875DRAFT 128396 Amylocarpus encephaloides
250	>KAF9691227.1 hypothetical protein EKO04 010655 Ascochyta lentis
251	>XP 033560892.1 uncharacterized protein BU25DRAFT 440658 Macroventuria anomochaeta
252	>XP 033448601.1 uncharacterized protein M421DRAFT 420893 Didymella exigua CBS 183.55
253	>tr|R0IGK7|R0IGK7 SETT2 Carbohydrate-binding module family 1 protein OS=Setosphaeria turcica (strain 28A) OX=671987 GN=SETTUDRAFT 164545 PE=3 SV=1
254	>tr|E7D6C5|E7D6C5 STABI Cellobiose dehydrogenase OS=Stachybotrys bisbyi OX=80385 GN=cdhIIA2 PE=2 SV=1
255	>tr|B2WHI7|B2WHI7 PYRTR Cellobiose dehydrogenase OS=Pyrenophora tritici-repentis (strain Pt-1C-BFP) OX=426418 GN=PTRG 09446 PE=3 SV=1
256	>tr|A0A6A7AS14|A0A6A7AS14 9PLEO GMC OxRdtase N domain-containing protein OS=Plenodomus tracheiphilus IPT5 OX=1408161 GN=T440DRAFT 435637 PE=3 SV=1
257	>tr|A0A6A7A0D4|A0A6A7A0D4 9PLEO CBM1 domain-containing protein OS=Ophiobolus disseminans OX=1469910 GN=CC86DRAFT 349860 PE=3 SV=1
258	>tr|A0A439D9J1|A0A439D9J1 9PEZI GMC OxRdtase N domain-containing protein OS=Xylaria grammica OX=363999 GN=EKO27 g4039 PE=3 SV=1
259	>tr|A0A2V1E2F9|A0A2V1E2F9 9PLEO Cellobiose dehydrogenase OS=Periconia macrospinosa OX=97972 GN=DM02DRAFT 669144 PE=3 SV=1
260	>tr|A0A1Y1YHK1|A0A1Y1YHK1 9PLEO CBM1 domain-containing protein OS=Clohesyomyces aquaticus OX=1231657 GN=BCR34DRAFT 577937 PE=3 SV=1
261	>tr|A0A178B2I0|A0A178B2I0 9PLEO FAD/NAD(P)-binding domain-containing protein OS=Stagonospora sp. SRC1lsM3a OX=765868 GN=IQ06DRAFT 214351 PE=3 SV=1
262	>tr|A0A094ARR0|A0A094ARR0 9PEZI GMC OxRdtase N domain-containing protein OS=Pseudogymnoascus sp. VKM F-4281 (FW-2241) OX=1420906 GN=V493 00537 PE=3 SV=1
263	>KIL84989.1 cellobiose dehydrogenase Fusarium avenaceum
264	>KAH8172372.1 cytochrome domain of cellobiose dehydrogenase domain-containing protein Sarocladium implicatum
265	>KAH7413846.1 hypothetical protein DE146DRAFT 638734 Phaeosphaeria sp. MPI-PUGE-AT-0046c
266	>KAH7323036.1 cellobiose dehydrogenase Stachybotrys elegans
267	>KAH6959441.1 hypothetical protein DER45DRAFT 505083 partial Fusarium tricinctum
268	>KAH6668785.1 putative cellobiose dehydrogenase Halenospora varia
269	>KAF7551666.1 cellobiose dehydrogenase Stylonectria norvegica
270	>KAF3903981.1 hypothetical protein AA313 de0201282 Arthrobotrys entomopaga
271	>KAF2022852.1 cellobiose dehydrogenase-like protein Setomelanomma holmii
272	>KAF1363574.1 cellobiose dehydrogenase-like protein Lizonia empirigonia
273	>XP 035338892.1 fungal cellulose binding domain-containing protein Colletotrichum scovillei
274	>XP 033437443.1 Cellobiose dehydrogenase Daldinia childiae
275	>tr|S8BYK0|S8BYK0 DACHA CBM1 domain-containing protein OS=Dactylellina haptotyla (strain CBS 200.50) OX=1284197 GN=H072 5775 PE=3 SV=1
276	>tr|E7D6C2|E7D6C2 9PEZI Cellobiose dehydrogenase OS=Hypoxylon haematostroma OX=152305 GN=cdhIIA PE=2 SV=1
277	>tr|A0A4V1XD30|A0A4V1XD30 9PEZI CBM1 domain-containing protein OS=Monosporascus sp. GIB2 OX=2211647 GN=DL765 007585 PE=3 SV=1
278	>tr|A0A4Q4ZYC0|A0A4Q4ZYC0 9PEZI Uncharacterized protein OS=Monosporascus sp. CRB-9-2 OX=2211643 GN=DL770 000584 PE=3 SV=1
279	>tr|A0A4Q4ZJM2|A0A4Q4ZJM2 9PEZI CBM1 domain-containing protein OS=Monosporascus sp. CRB-8-3 OX=2211644 GN=DL769 000270 PE=3 SV=1
280	>tr|A0A4Q4Y8F7|A0A4Q4Y8F7 9PEZI CBM1 domain-containing protein OS=Monosporascus sp. 5C6A OX=2211642 GN=DL771 005473 PE=3 SV=1
281	>tr|A0A4Q4W0L0|A0A4Q4W0L0 9PEZI CBM1 domain-containing protein OS=Monosporascus sp. MG133 OX=2211645 GN=DL767 002131 PE=3 SV=1
282	>tr|A0A4Q4T7N1|A0A4Q4T7N1 9PEZI CBM1 domain-containing protein OS=Monosporascus ibericus OX=155417 GN=DL764 006654 PE=3 SV=1
283	>tr|A0A423WN32|A0A423WN32 9PEZI CBM1 domain-containing protein OS=Cytospora leucostoma OX=1230097 GN=VPNG 07433 PE=3 SV=1
284	>tr|A0A3N4JSG4|A0A3N4JSG4 9PEZI FAD/NAD(P)-binding domain-containing protein OS=Choiromyces venosus 120613-1 OX=1336337 GN=L873DRAFT 670238 PE=3 SV=1
285	>tr|A0A1Y2WL44|A0A1Y2WL44 9PEZI Carbohydrate-binding module family 1 protein OS=Daldinia sp. EC12 OX=1001832 GN=K445DRAFT 270957 PE=3 SV=1
286	>tr|A0A1Y2UA75|A0A1Y2UA75 9PEZI Carbohydrate-binding module family 1 protein OS=Hypoxylon sp. CO27-5 OX=1001938 GN=M434DRAFT 38446 PE=3 SV=1
287	>tr|A0A1E1L9J5|A0A1E1L9J5 9HELO Related to cellobiose dehydrogenase OS=Rhynchosporium agropyri OX=914238 GN=RAG0 12763 PE=3 SV=1
288	>tr|A0A1E1JTH6|A0A1E1JTH6 9HELO Probable cellobiose dehydrogenase OS=Rhynchosporium commune OX=914237 GN=RCO7 04718 PE=3 SV=1
289	>tr|A0A1B8E0J3|A0A1B8E0J3 9PEZI GMC OxRdtase N domain-containing protein OS=Pseudogymnoascus sp. 23342-1-I1 OX=1524831 GN=VE03 05847 PE=3 SV=1
290	>tr|A0A0G2I821|A0A0G2I821 9PEZI Putative cellobiose dehydrogenase OS=Diaporthe ampelina OX=1214573 GN=UCDDA912 g04068 PE=3 SV=1
291	>tr|A0A0G2EHA2|A0A0G2EHA2 9PEZI Putative cellobiose dehydrogenase OS=Diplodia seriata OX=420778 GN=UCDDS831 g03791 PE=3 SV=1
292	>KAH7239448.1 hypothetical protein BKA59DRAFT 403306 Fusarium tricinctum
293	>KAG9255545.1 cellobiose dehydrogenase-like protein Emericellopsis atlantica
294	>KAG7113121.1 Cellobiose dehydrogenase like protein Verticillium longisporum
295	>KAF7529403.1 cellobiose dehydrogenase Neopestalotiopsis clavispora
296	>KAF2269322.1 cellobiose dehydrogenase-like protein Didymosphaeria enalia
297	>XP 036533985.1 cellobiose dehydrogenase Fusarium subglutinans

298	>XP 033680843.1 uncharacterized protein BU26DRAFT 461196 Trematosphaeria pertusa
299	>XP 031089964.1 probable cellobiose dehydrogenase Fusarium proliferatum ET1
300	>tr|W7HZX9|W7HZX9 9PEZI CBM1 domain-containing protein OS=Drechslerella stenobrocha 248 OX=1043628 GN=DRE 05119 PE=3 SV=1
301	>tr|R1H3M7|R1H3M7 BOTPV Putative cellobiose dehydrogenase protein OS=Botryosphaeria parva (strain UCR-NP2) OX=1287680 GN=UCRNP2 206 PE=3 SV=1
302	>tr|K3VZJ1|K3VZJ1 FUSPC CBM1 domain-containing protein OS=Fusarium pseudograminearum (strain CS3096) OX=1028729 GN=FPSE 07371 PE=3 SV=1
303	>tr|E7D6C4|E7D6C4 STABI Cellobiose dehydrogenase OS=Stachybotrys bisbyi OX=80385 GN=cdhIIA1 PE=2 SV=1
304	>tr|A0A6A6SVN6|A0A6A6SVN6 9PLEO GMC OxRdtase N domain-containing protein OS=Lophiostoma macrostomum CBS 122681 OX=1314788 GN=K491DRAFT 720716 PE=3 SV=1
305	>tr|A0A420T913|A0A420T913 GIBIN Cellobiose dehydrogenase OS=Gibberella intermedia OX=948311 GN=BFJ72 g7477 PE=3 SV=1
306	>tr|A0A395T986|A0A395T986 9HYPO Cellobiose dehydrogenase OS=Fusarium longipes OX=694270 GN=FLONG3 458 PE=3 SV=1
307	>tr|A0A2T4GFE2|A0A2T4GFE2 FUSCU Cellobiose dehydrogenase OS=Fusarium culmorum OX=5516 GN=FCULG 00011935 PE=3 SV=1
308	>tr|A0A2I0RZK0|A0A2I0RZK0 9PEZI Cellobiose dehydrogenase OS=Cercospora zeina OX=348901 GN=BST61 czeina21g001010 PE=3 SV=1
309	>tr|A0A1Y2VRU6|A0A1Y2VRU6 9PEZI Cellobiose dehydrogenase protein OS=Hypoxylon sp. CI-4A OX=1001833 GN=M426DRAFT 267728 PE=3 SV=1
310	>tr|A0A177CAW9|A0A177CAW9 9PLEO Cellobiose dehydrogenase-like protein OS=Paraphaeosphaeria sporulosa OX=1460663 GN=CC84DRAFT 1121628 PE=3 SV=1
311	>tr|A0A096PCY6|A0A096PCY6 FUSPS WGS project CBMD000000000 data contig CS3427 c001274 OS=Fusarium pseudograminearum CS3427 OX=1318457 GN=BN847 0121770 PE=3 SV=1
312	>tr|A0A094G1L0|A0A094G1L0 9PEZI GMC OxRdtase N domain-containing protein OS=Pseudogymnoascus sp. VKM F-4517 (FW-2822) OX=1420911 GN=V498 02385 PE=3 SV=1
313	>tr|A0A094BXH2|A0A094BXH2 9PEZI Uncharacterized protein OS=Pseudogymnoascus sp. VKM F-4513 (FW-928) OX=1420907 GN=V494 00482 PE=3 SV=1
314	>tr|A0A094ACL1|A0A094ACL1 9PEZI GMC OxRdtase N domain-containing protein OS=Pseudogymnoascus sp. VKM F-4281 (FW-2241) OX=1420906 GN=V493 03549 PE=3 SV=1
315	>KNG51801.1 carbohydrate-binding module family 1 protein Stemphylium lycopersici
316	>KAH8752504.1 cellobiose dehydrogenase Diaporthaceae sp. PMI 573
317	>KAH8194055.1 hypothetical protein TruAng 011785 Truncatella angustata
318	>KAH6659192.1 fungal cellulose binding domain-containing protein Truncatella angustata
319	>KAG5759280.1 hypothetical protein H9Q72 012593 Fusarium xylarioides
320	>KAF9737269.1 fungal cellulose binding domain-containing protein Paraphaeosphaeria minitans
321	>KAF5689942.1 cellobiose dehydrogenase Fusarium globosum
322	>KAF5618450.1 cellobiose dehydrogenase Fusarium sp. NRRL 25303
323	>KAF5615421.1 cellobiose dehydrogenase Fusarium sp. NRRL 52700
324	>KAF5568912.1 cellobiose dehydrogenase Fusarium phyllophilum
325	>KAF5539142.1 cellobiose dehydrogenase Fusarium mexicanum
326	>KAF5243256.1 hypothetical protein FANTH 8265 Fusarium anthophilum
327	>KAF4946967.1 hypothetical protein FGADI 10783 Fusarium gaditjirri
328	>KAF4478491.1 cellobiose dehydrogenase Fusarium agapanthi
329	>KAF4313866.1 cellobiose dehydrogenase Botryosphaeria dothidea
330	>KAF3924203.1 hypothetical protein ABW21 db0201788 Drechslerella brochopaga
331	>KAF2734650.1 hypothetical protein EJ04DRAFT 512353 Polyplosphaeria fusca
332	>KAF2203798.1 hypothetical protein GQ43DRAFT 389286 Delitschia confertaspora ATCC 74209
333	>KAF1937015.1 cellobiose dehydrogenase Clathrospora elynae
334	>XP 045268095.1 Cellobiose dehydrogenase Colletotrichum gloeosporioides
335	>XP 043168982.1 uncharacterized protein ALTATR162 LOCUS5428 Alternaria atra
336	>XP 040790305.1 uncharacterized protein K460DRAFT 307263 Cucurbitaria berberidis CBS 394.84
337	>XP 037202422.1 cellobiose dehydrogenase Fusarium tjaetaba
338	>XP 037173487.1 Cellobiose dehydrogenase Colletotrichum aenigma
339	>XP 036589598.1 fungal cellulose binding domain-containing protein Colletotrichum truncatum
340	>XP 033389650.1 uncharacterized protein BU24DRAFT 19012 Aaosphaeria arxii CBS 175.79
341	>XP 028502145.1 Cellobiose dehydrogenase Alternaria arborescens
342	>tr|E5A953|E5A953 LEPMJ Similar to cellobiose dehydrogenase OS=Leptosphaeria maculans (strain JN3 / isolate v23.1.3 / race Av1-4-5-6-7-8) OX=985895 GN=LEMA P013240.1 PE=3 SV=1
343	>tr|A0A6A6HCI1|A0A6A6HCI1 9PEZI GMC OxRdtase N domain-containing protein OS=Viridothelium virens OX=1048519 GN=EV356DRAFT 98225 PE=3 SV=1
344	>tr|A0A5Q4BDH2|A0A5Q4BDH2 9PEZI Cellobiose dehydrogenase (Fragment) OS=Colletotrichum shisoi OX=2078593 GN=CDH-1-1 PE=3 SV=1
345	>tr|A0A4T0VJG7|A0A4T0VJG7 9PEZI Cellobiose dehydrogenase OS=Colletotrichum higginsianum OX=80884 GN=CH35J 010241 PE=3 SV=1
346	>tr|A0A437A6R7|A0A437A6R7 9PEZI CBM1 domain-containing protein OS=Arthrobotrys flagrans OX=97331 GN=DFL 004979 PE=3 SV=1
347	>tr|A0A395RLA5|A0A395RLA5 FUSSP Cellobiose dehydrogenase OS=Fusarium sporotrichioides OX=5514 GN=FSPOR 10387 PE=3 SV=1
348	>tr|A0A366PUA9|A0A366PUA9 GIBMO CBM1 domain-containing protein OS=Gibberella moniliformis OX=117187 GN=FVER53263 13310 PE=3 SV=1
349	>tr|A0A2L2TWX7|A0A2L2TWX7 9HYPO CBM1 domain-containing protein OS=Fusarium venenatum OX=56646 PE=3 SV=1
350	>tr|A0A2K0UUB2|A0A2K0UUB2 GIBNY CBM1 domain-containing protein OS=Gibberella nygamai OX=42673 GN=FNYG 13846 PE=3 SV=1
351	>tr|A0A1B8AQB6|A0A1B8AQB6 FUSPO CBM1 domain-containing protein OS=Fusarium poae OX=36050 GN=FPOA 09064 PE=3 SV=1
352	>tr|A0A194VM16|A0A194VM16 9PEZI Cellobiose dehydrogenase OS=Valsa mali OX=105487 GN=VM1G 00310 PE=3 SV=1
353	>tr|A0A178DGE0|A0A178DGE0 9PLEO CBM1 domain-containing protein OS=Pyrenochaeta sp. DS3sAY3a OX=765867 GN=IQ07DRAFT 593277 PE=3 SV=1
354	>tr|A0A0M9EMT2|A0A0M9EMT2 FUSLA Cellobiose dehydrogenase OS=Fusarium langsethiae OX=179993 GN=FLAG1 10968 PE=3 SV=1
355	>KAH6881684.1 cellobiose dehydrogenase Alternaria rosae
356	>KAF5968037.1 cellobiose dehydrogenase Fusarium coicis
357	>KAF5708158.1 cellobiose dehydrogenase Fusarium mundagurra
358	>KAF5684191.1 cellobiose dehydrogenase Fusarium denticulatum
359	>KAF5670108.1 cellobiose dehydrogenase Fusarium heterosporum
360	>KAF5581619.1 cellobiose dehydrogenase Fusarium pseudocircinatum
361	>KAF5572589.1 cellobiose dehydrogenase Fusarium pseudoanthophilum
362	>KAF5562356.1 cellobiose dehydrogenase Fusarium napiforme
363	>KAF4996651.1 hypothetical protein FGRMN 4397 Fusarium graminum
364	>KAF4439000.1 cellobiose dehydrogenase Fusarium acutatum
365	>KAF2871105.1 cellobiose dehydrogenase-like protein Massariosphaeria phaeospora
366	>KAF2113597.1 cellobiose dehydrogenase-like protein Lophiotrema nucula
367	>KAF0316720.1 cellobiose dehydrogenase Colletotrichum asianum
368	>KAB2109362.1 Cellobiose dehydrogenase Alternaria gaisen
369	>XP 022475040.1 fungal cellulose binding domain-containing protein Colletotrichum orchidophilum
370	>tr|M7SZY2|M7SZY2 EUTLA Putative cellobiose dehydrogenase protein OS=Eutypa lata (strain UCR-EL1) OX=1287681 GN=UCREL1 764 PE=3 SV=1
371	>tr|A0A6A6FF87|A0A6A6FF87 9PEZI CBM1 domain-containing protein OS=Cercospora zeae-maydis SCOH1-5 OX=717836 GN=CERZMDRAFT 85020 PE=3 SV=1
372	>tr|A0A4Q4ZSA8|A0A4Q4ZSA8 9PEZI GMC OxRdtase N domain-containing protein OS=Monosporascus sp. CRB-9-2 OX=2211643 GN=DL770 002810 PE=3 SV=1
373	>tr|A0A4Q4YUP0|A0A4Q4YUP0 9PEZI MFS domain-containing protein OS=Monosporascus sp. 5C6A OX=2211642 GN=DL771 000550 PE=3 SV=1
374	>tr|A0A4Q4WLB5|A0A4Q4WLB5 9PEZI GMC OxRdtase N domain-containing protein OS=Monosporascus sp. mg162 OX=1081914 GN=DL768 005866 PE=3 SV=1
375	>tr|A0A4Q4VAL7|A0A4Q4VAL7 9PEZI GMC OxRdtase N domain-containing protein OS=Monosporascus sp. MG133 OX=2211645 GN=DL767 006136 PE=3 SV=1
376	>tr|A0A4Q4TIK6|A0A4Q4TIK6 9PEZI GMC OxRdtase N domain-containing protein OS=Monosporascus sp. GIB2 OX=2211647 GN=DL765 009854 PE=3 SV=1
377	>tr|A0A4Q4SS17|A0A4Q4SS17 9PEZI GMC OxRdtase N domain-containing protein OS=Monosporascus ibericus OX=155417 GN=DL764 010817 PE=3 SV=1
378	>tr|A0A395MYH2|A0A395MYH2 9HYPO Cellobiose dehydrogenase OS=Fusarium flagelliforme OX=2675880 GN=FIE12Z 2735 PE=3 SV=1
379	>tr|A0A366SA76|A0A366SA76 9HYPO CBM1 domain-containing protein OS=Fusarium coffeatum OX=231269 GN=FIESC28 01007 PE=3 SV=1
380	>tr|A0A2P5HX67|A0A2P5HX67 9PEZI Fungal cellulose binding domain-containing protein OS=Diaporthe helianthi OX=158607 GN=DHEL01 v206757 PE=3 SV=1
381	>tr|A0A2B7XRL9|A0A2B7XRL9 9EURO Uncharacterized protein OS=Helicocarpus griseus UAMH5409 OX=1447875 GN=AJ79 04902 PE=3 SV=1
382	>tr|A0A135V878|A0A135V878 9PEZI Fungal cellulose binding domain-containing protein OS=Colletotrichum salicis OX=1209931 GN=CSAL01 08445 PE=3 SV=1
383	>tr|A0A0F4ZL77|A0A0F4ZL77 9PEZI CBM1 domain-containing protein OS=Thielaviopsis punctulata OX=72032 GN=TD95 000828 PE=3 SV=1
384	>OCL02221.1 carbohydrate-binding module family 1 protein Glonium stellatum
385	>KAF3939799.1 hypothetical protein ABW19 dt0201598 Dactylella cylindrospora
386	>KAF3901704.1 hypothetical protein ABW20 dc0106822 Dactylellina cionopaga
387	>CAG7565365.1 unnamed protein product Fusarium equiseti
388	>XP 044642359.1 uncharacterized protein KVR01 008592 Diaporthe batatas
389	>XP 038790100.1 cellobiose dehydrogenase Alternaria burnsii
390	>tr|G1XR12|G1XR12 ARTOA CBM1 domain-containing protein OS=Arthrobotrys oligospora (strain ATCC 24927 / CBS 115.81 / DSM 1491) OX=756982 GN=AOL s00193g11 PE=3 SV=1
391	>tr|A0A7C8Q8H4|A0A7C8Q8H4 ORBOL CBM1 domain-containing protein OS=Orbilia oligospora OX=2813651 GN=TWF106 002478 PE=3 SV=1
392	>tr|A0A4U6X2K9|A0A4U6X2K9 9PEZI Cellobiose dehydrogenase OS=Colletotrichum tanaceti OX=1306861 GN=CDH-1 PE=3 SV=1
393	>tr|A0A420TAN1|A0A420TAN1 FUSOX Cellobiose dehydrogenase OS=Fusarium oxysporum OX=5507 GN=BFJ70 g6227 PE=3 SV=1
394	>tr|A0A219WGI5|A0A219WGI5 TERCL Cellobiose dehydrogenase OS=Termitomyces clypeatus OX=182030 PE=2 SV=1
395	>tr|A0A084RDP7|A0A084RDP7 STACH CBM1 domain-containing protein OS=Stachybotrys chartarum IBT 40288 OX=1283842 GN=S40288 03693 PE=3 SV=1
396	>tr|A0A066X3H0|A0A066X3H0 COLSU Putative fungal cellulose binding domain-containing protein OS=Colletotrichum sublineola OX=1173701 GN=CSUB01 06400 PE=3 SV=1
397	>KAH7481047.1 Cellobiose dehydrogenase Fusarium oxysporum f. sp. matthiolae
398	>KAH7224426.1 hypothetical protein BKA55DRAFT 655775 Fusarium redolens
399	>KAH6655717.1 cellobiose dehydrogenase Truncatella angustata

<table>
<tr><th></th><th></th></tr>
<tr><td>400</td><td>>KAG7411088.1 Cellobiose dehydrogenase Fusarium oxysporum f. sp. rapae</td></tr>
<tr><td>401</td><td>>KAF9774088.1 hypothetical protein IL306 007960 Fusarium sp. DS 682</td></tr>
<tr><td>402</td><td>>KAF4440228.1 putative cellobiose dehydrogenase Fusarium austroafricanum</td></tr>
<tr><td>403</td><td>>KAF3053995.1 hypothetical protein E8E11 005116 Didymella keratinophila</td></tr>
<tr><td>404</td><td>>KAF3045992.1 hypothetical protein E8E12 009990 Didymella heteroderae</td></tr>
<tr><td>405</td><td>>GJC79642.1 cellobiose dehydrogenase Colletotrichum spaethianum</td></tr>
<tr><td>406</td><td>>EXL50294.1 hypothetical protein FOCG 08599 Fusarium oxysporum f. sp. radicis-lycopersici 26381</td></tr>
<tr><td>407</td><td>>tr|N4VHA3|N4VHA3 COLOR Cellobiose dehydrogenase OS=Colletotrichum orbiculare (strain 104-T / ATCC 96160 / CBS 514.97 / LARS 414 / MAFF 240422) OX=1213857 GN=CDH-1-2 PE=3 SV=1</td></tr>
<tr><td>408</td><td>>tr|G2X591|G2X591 VERDV Cellobiose dehydrogenase OS=Verticillium dahliae (strain VdLs.17 / ATCC MYA-4575 / FGSC 10137) OX=498257 GN=VDAG 05396 PE=3 SV=1</td></tr>
<tr><td>409</td><td>>tr|A0A4V6QE96|A0A4V6QE96 9PEZI Cellobiose dehydrogenase OS=Colletotrichum spinosum OX=1347390 GN=CDH-1-0 PE=3 SV=1</td></tr>
<tr><td>410</td><td>>tr|A0A423VTV1|A0A423VTV1 9PEZI CBM1 domain-containing protein OS=Valsa sordida OX=252740 GN=VSDG 05939 PE=3 SV=1</td></tr>
<tr><td>411</td><td>>tr|A0A3D8RYI6|A0A3D8RYI6 9EURO Uncharacterized protein OS=Aspergillus mulundensis OX=1810919 GN=DSM5745 05964 PE=3 SV=1</td></tr>
<tr><td>412</td><td>>tr|A0A0G4N0Z5|A0A0G4N0Z5 9PEZI CBM1 domain-containing protein OS=Verticillium longisporum OX=100787 GN=BN1708 008084 PE=3 SV=1</td></tr>
<tr><td>413</td><td>>tr|A0A084QKV5|A0A084QKV5 STAC4 CBM1 domain-containing protein OS=Stachybotrys chlorohalonata (strain IBT 40285) OX=1283841 GN=S40285 09344 PE=3 SV=1</td></tr>
<tr><td>414</td><td>>tr|A0A084QG53|A0A084QG53 STAC4 CBM1 domain-containing protein OS=Stachybotrys chlorohalonata (strain IBT 40285) OX=1283841 GN=S40285 04393 PE=3 SV=1</td></tr>
<tr><td>415</td><td>>OCL14160.1 cellobiose dehydrogenase partial Glonium stellatum</td></tr>
<tr><td>416</td><td>>KAF6834738.1 cellobiose dehydrogenase Colletotrichum musicola</td></tr>
<tr><td>417</td><td>>KAF6822263.1 cellobiose dehydrogenase Colletotrichum plurivorum</td></tr>
<tr><td>418</td><td>>XP 033596331.1 cellobiose dehydrogenase Pseudovirgaria hyperparasitica</td></tr>
<tr><td>419</td><td>>tr|E3QNF6|E3QNF6 COLGM Fungal cellulose binding domain-containing protein OS=Colletotrichum graminicola (strain M1.001 / M2 / FGSC 10212) OX=645133 GN=GLRG 07538 PE=3 SV=1</td></tr>
<tr><td>420</td><td>>tr|A0A507B7Z4|A0A507B7Z4 9PEZI GMC OxRdtase N domain-containing protein OS=Phialemoniopsis curvata OX=1093900 GN=E0L32 000147 PE=3 SV=1</td></tr>
<tr><td>421</td><td>>tr|A0A4U0VEB4|A0A4U0VEB4 9PEZI Uncharacterized protein OS=Friedmanniomyces endolithicus OX=329885 GN=B0A54 02328 PE=3 SV=1</td></tr>
<tr><td>422</td><td>>tr|A0A423W726|A0A423W726 9PEZI CBM1 domain-containing protein OS=Valsa malicola OX=356882 GN=VMCG 06665 PE=3 SV=1</td></tr>
<tr><td>423</td><td>>tr|A0A2N3NFW8|A0A2N3NFW8 9PEZI CBM1 domain-containing protein OS=Lomentospora prolificans OX=41688 GN=jhhlp 003090 PE=3 SV=1</td></tr>
<tr><td>424</td><td>>tr|A0A084FYA1|A0A084FYA1 PSEDA Xyloglucan-specific exo-beta-14-glucanase OS=Pseudallescheria apiosperma OX=563466 GN=SAPIO CDS9070 PE=3 SV=1</td></tr>
<tr><td>425</td><td>>XP 044639396.1 uncharacterized protein KVR01 011687 Diaporthe batatas</td></tr>
<tr><td>426</td><td>>XP 043022198.1 uncharacterized protein INS49 012196 Diaporthe citri</td></tr>
<tr><td>427</td><td>>XP 040716697.1 cellobiose dehydrogenase Pseudomassariella vexata</td></tr>
<tr><td>428</td><td>>XP 028464229.1 cellobiose dehydrogenase Sodiomyces alkalinus F11</td></tr>
<tr><td>429</td><td>>tr|Q7S0Y1|Q7S0Y1 NEUCR Cellobiose dehydrogenase OS=Neurospora crassa (strain ATCC 24698 / 74-OR23-1A / CBS 708.71 / DSM 1257 / FGSC 987) OX=367110 GN=cdh-2 PE=3 SV=1</td></tr>
<tr><td>430</td><td>>tr|G4V131|G4V131 NEUT9 GMC OxRdtase N domain-containing protein OS=Neurospora tetrasperma (strain FGSC 2509 / P0656) OX=510952 GN=NEUTE2DRAFT 160289 PE=3 SV=1</td></tr>
<tr><td>431</td><td>>tr|F7W4V8|F7W4V8 SORMK WGS project CABT00000000 data contig 2.29 OS=Sordaria macrospora (strain ATCC MYA-333 / DSM 997 / K(L3346) / K-hell) OX=771870 GN=SMAC 06953 PE=3 SV=1</td></tr>
<tr><td>432</td><td>>tr|A0A2P5HZK2|A0A2P5HZK2 9PEZI Cellobiose dehydrogenase OS=Diaporthe helianthi OX=158607 GN=DHEL01 v205928 PE=3 SV=1</td></tr>
<tr><td>433</td><td>>tr|A0A0G2FE40|A0A0G2FE40 9PEZI Putative cellobiose dehydrogenase OS=Diaporthe ampelina OX=1214573 GN=UCDDA912 g07610 PE=3 SV=1</td></tr>
<tr><td>434</td><td>>tr|A0A0F8BLH1|A0A0F8BLH1 CERFI Cellobiose dehydrogenase OS=Ceratocystis fimbriata f. sp. platani OX=88771 GN=CDH-1 3 PE=3 SV=1</td></tr>
<tr><td>435</td><td>>tr|A0A0B0E3N2|A0A0B0E3N2 NEUCS GMC OxRdtase N domain-containing protein OS=Neurospora crassa OX=5141 GN=GE21DRAFT 9427 PE=3 SV=1</td></tr>
<tr><td>436</td><td>>KAH8784664.1 putative cellobiose dehydrogenase Hyaloscypha finlandica</td></tr>
<tr><td>437</td><td>>KAH8781783.1 putative cellobiose dehydrogenase Hyaloscypha sp. PMI 1271</td></tr>
<tr><td>438</td><td>>KAF2496966.1 cellobiose dehydrogenase Lophium mytilinum</td></tr>
<tr><td>439</td><td>>tr|K2RVZ2|K2RVZ2 MACPH Glucose-methanol-choline oxidoreductase OS=Macrophomina phaseolina (strain MS6) OX=1126212 GN=MPH 03764 PE=3 SV=1</td></tr>
<tr><td>440</td><td>>tr|A0A2C5XFU5|A0A2C5XFU5 9PEZI Cellobiose dehydrogenase OS=Ceratocystis fimbriata CBS 114723 OX=1035309 GN=CDH-1 1 PE=3 SV=1</td></tr>
<tr><td>441</td><td>>KAF7536217.1 cellobiose dehydrogenase Neopestalotiopsis clavispora</td></tr>
<tr><td>442</td><td>>KAF3004614.1 hypothetical protein E8E14 008457 Neopestalotiopsis sp. 37M</td></tr>
<tr><td>443</td><td>>KAF2455665.1 cellobiose dehydrogenase Lineolata rhizophorae</td></tr>
<tr><td>444</td><td>>tr|W3X8R3|W3X8R3 PESFW GMC OxRdtase N domain-containing protein OS=Pestalotiopsis fici (strain W106-1 / CGMCC3.15140) OX=1229662 GN=PFICI 06779 PE=3 SV=1</td></tr>
<tr><td>445</td><td>>KAH7368170.1 cellobiose dehydrogenase Plectosphaerella cucumerina</td></tr>
<tr><td>446</td><td>>KAG7124919.1 Cellobiose dehydrogenase like protein Verticillium longisporum</td></tr>
<tr><td>447</td><td>>XP 040715940.1 fungal cellulose binding domain-containing protein Pseudomassariella vexata</td></tr>
<tr><td>448</td><td>>XP 023628271.1 related to cellobiose dehydrogenase Ramularia collo-cygni</td></tr>
<tr><td>449</td><td>>tr|A0A4U0UY87|A0A4U0UY87 9PEZI CBM1 domain-containing protein OS=Friedmanniomyces endolithicus OX=329885 GN=B0A54 07625 PE=3 SV=1</td></tr>
<tr><td>450</td><td>>tr|A0A3N4IRD6|A0A3N4IRD6 ASCIM CBM1 domain-containing protein OS=Ascobolus immersus RN42 OX=1160509 GN=BJ508DRAFT 202535 PE=3 SV=1</td></tr>
<tr><td>451</td><td>>tr|A0A2J6QKR6|A0A2J6QKR6 9HELO GMC OxRdtase N domain-containing protein OS=Hyaloscypha hepaticicola OX=2082293 GN=NA56DRAFT 667964 PE=3 SV=1</td></tr>
<tr><td>452</td><td>>XP 044698466.1 uncharacterized protein H6S33 009651 Morchella sextelata</td></tr>
<tr><td>453</td><td>>tr|A0A3N4L930|A0A3N4L930 9PEZI FAD/NAD(P)-binding domain-containing protein OS=Morchella conica CCBAS932 OX=1392247 GN=P167DRAFT 557737 PE=3 SV=1</td></tr>
<tr><td>454</td><td>>tr|A0A3M7J8A4|A0A3M7J8A4 HORWE CBM1 domain-containing protein OS=Hortaea werneckii OX=91943 GN=D0859 01858 PE=3 SV=1</td></tr>
<tr><td>455</td><td>>tr|A0A3M7IJ27|A0A3M7IJ27 HORWE CBM1 domain-containing protein OS=Hortaea werneckii OX=91943 GN=D0859 10466 PE=3 SV=1</td></tr>
<tr><td>456</td><td>>tr|A0A3M7GKK5|A0A3M7GKK5 HORWE CBM1 domain-containing protein OS=Hortaea werneckii OX=91943 GN=D0860 07649 PE=3 SV=1</td></tr>
<tr><td>457</td><td>>tr|A0A3M7FEF8|A0A3M7FEF8 HORWE CBM1 domain-containing protein OS=Hortaea werneckii OX=91943 GN=D0861 05461 PE=3 SV=1</td></tr>
<tr><td>458</td><td>>tr|A0A139HG69|A0A139HG69 9PEZI Uncharacterized protein OS=Pseudocercospora eumusae OX=321146 GN=AC578 9541 PE=3 SV=1</td></tr>
<tr><td>459</td><td>>KAH8887786.1 hypothetical protein GQ53DRAFT 872520 Thozetella sp. PMI 491</td></tr>
<tr><td>460</td><td>>tr|J3P869|J3P869 GAET3 Uncharacterized protein OS=Gaeumannomyces tritici (strain R3-111a-1) OX=644352 GN=20350162 PE=3 SV=1</td></tr>
<tr><td>461</td><td>>tr|A0A0C4DS53|A0A0C4DS53 MAGP6 Uncharacterized protein OS=Magnaporthiopsis poae (strain ATCC 64411 / 73-15) OX=644358 GN=MAPG 02728 PE=3 SV=1</td></tr>
<tr><td>462</td><td>>KAH6663555.1 fungal cellulose binding domain-containing protein Plectosphaerella plurivora</td></tr>
<tr><td>463</td><td>>XP 024726713.1 uncharacterized protein K444DRAFT 648721 Hyaloscypha bicolor E</td></tr>
<tr><td>464</td><td>>XP 040772113.1 uncharacterized protein M406DRAFT 64170 Cryphonectria parasitica EP155</td></tr>
<tr><td>465</td><td>>XP 033673698.1 carbohydrate-binding module family 1 protein Zasmidium cellare ATCC 36951</td></tr>
<tr><td>466</td><td>>tr|A0A4U0XZ00|A0A4U0XZ00 9PEZI CBM1 domain-containing protein (Fragment) OS=Friedmanniomyces simplex OX=329884 GN=B0A55 02376 PE=3 SV=1</td></tr>
<tr><td>467</td><td>>tr|M3AYA9|M3AYA9 PSEFD Carbohydrate-binding module family 1 protein/Non-catalytic module family CDH OS=Pseudocercospora fijiensis (strain CIRAD86) OX=383855 GN=MYCFIDRAFT 215593 PE=3 SV</td></tr>
<tr><td>468</td><td>>tr|A0A4U0U3H6|A0A4U0U3H6 9PEZI Uncharacterized protein OS=Hortaea thailandica OX=706561 GN=B0A50 03459 PE=3 SV=1</td></tr>
<tr><td>469</td><td>>tr|A0A150VAE5|A0A150VAE5 9PEZI Uncharacterized protein OS=Acidomyces richmondensis BFW OX=766039 GN=M433DRAFT 2830 PE=3 SV=1</td></tr>
<tr><td>470</td><td>>XP 029750062.1 hypothetical protein PpBr36 05357 Pyricularia pennisetigena</td></tr>
<tr><td>471</td><td>>tr|L7J170|L7J170 MAGOP Cellobiose dehydrogenase OS=Magnaporthe oryzae (strain P131) OX=1143193 GN=OOW P131scaffold01138g36 PE=3 SV=1</td></tr>
<tr><td>472</td><td>>tr|A0A6P8BCC4|A0A6P8BCC4 MAGGR uncharacterized protein OS=Magnaporthe grisea OX=148305 GN=PgNI 03000 PE=3 SV=1</td></tr>
<tr><td>473</td><td>>TLD20394.1 hypothetical protein PspLS 08540 Pyricularia sp. CBS 133598</td></tr>
<tr><td>474</td><td>>tr|A0A3M7H1K1|A0A3M7H1K1 HORWE CBM1 domain-containing protein OS=Hortaea werneckii OX=91943 GN=D0862 04339 PE=3 SV=1</td></tr>
<tr><td>475</td><td>>tr|A0A3M7DMS3|A0A3M7DMS3 HORWE CBM1 domain-containing protein OS=Hortaea werneckii OX=91943 GN=D0863 09177 PE=3 SV=1</td></tr>
<tr><td>476</td><td>>tr|A0A3M7D4W9|A0A3M7D4W9 HORWE CBM1 domain-containing protein OS=Hortaea werneckii OX=91943 GN=D0865 02126 PE=3 SV=1</td></tr>
<tr><td>477</td><td>>tr|A0A3M7AUW4|A0A3M7AUW4 HORWE Uncharacterized protein OS=Hortaea werneckii OX=91943 GN=D0866 07430 PE=3 SV=1</td></tr>
<tr><td>478</td><td>>KAE9373117.1 hypothetical protein N431DRAFT 438484 Chalara longipes BDJ</td></tr>
<tr><td>479</td><td>>tr|A0A2S6BTF8|A0A2S6BTF8 9PEZI CBM1 domain-containing protein OS=Cercospora berteroae OX=357750 GN=CBER1 08121 PE=3 SV=1</td></tr>
<tr><td>480</td><td>>tr|A0A1V8USU8|A0A1V8USU8 9PEZI CBM1 domain-containing protein OS=Rachicladosporium sp. CCFEE 5018 OX=1974281 GN=B0A51 08275 PE=3 SV=1</td></tr>
<tr><td>481</td><td>>tr|A0A1V8TRW3|A0A1V8TRW3 9PEZI CBM1 domain-containing protein OS=Rachicladosporium antarcticum OX=1507870 GN=B0A48 00917 PE=3 SV=1</td></tr>
<tr><td>482</td><td>>tr|M2YMP3|M2YMP3 DOTSN Uncharacterized protein OS=Dothistroma septosporum (strain NZE10 / CBS 128990) OX=675120 GN=DOTSEDRAFT 153605 PE=3 SV=1</td></tr>
<tr><td>483</td><td>>KAH3650691.1 Cellobiose dehydrogenase Fulvia fulva</td></tr>
<tr><td>484</td><td>>XP 044661807.1 uncharacterized protein CKM354 001041500 Cercospora kikuchii</td></tr>
<tr><td>485</td><td>>XP 023451360.1 Cellobiose dehydrogenase Cercospora beticola</td></tr>
<tr><td>486</td><td>>tr|A0A2J6RTE8|A0A2J6RTE8 9HELO Cellobiose dehydrogenase protein OS=Hyaloscypha variabilis F OX=1149755 GN=L207DRAFT 582213 PE=3 SV=1</td></tr>
<tr><td>487</td><td>>tr|S3BP98|S3BP98 OPHP1 Cellobiose dehydrogenase OS=Ophiostoma piceae (strain UAMH 11346) OX=1262450 GN=F503 08708 PE=3 SV=1</td></tr>
<tr><td>488</td><td>>tr|N1QJA2|N1QJA2 SPHMS FAD/NAD(P)-binding domain-containing protein OS=Sphaerulina musiva (strain SO2202) OX=692275 GN=SEPMUDRAFT 67241 PE=3 SV=1</td></tr>
<tr><td>489</td><td>>tr|A0A2P7YL03|A0A2P7YL03 9PEZI Cellobiose dehydrogenase OS=Elsinoe australis OX=40998 GN=B9Z65 1839 PE=3 SV=1</td></tr>
<tr><td>490</td><td>>tr|A0A5N5MIY7|A0A5N5MIY7 9PEZI GMC OxRdtase N domain-containing protein OS=Coniochaeta sp. 2T2.1 OX=1571157 GN=GE09DRAFT 1173764 PE=3 SV=1</td></tr>
<tr><td>491</td><td>>tr|A0A5N5L9R6|A0A5N5L9R6 9PEZI GMC OxRdtase N domain-containing protein OS=Coniochaeta sp. 2T2.1 OX=1571157 GN=GE09DRAFT 1176268 PE=3 SV=1</td></tr>
<tr><td>492</td><td>>tr|A0A2I7VT65|A0A2I7VT65 9PEZI Cellobiose dehydrogenase OS=Coniochaeta hoffmannii OX=91930 PE=3 SV=1</td></tr>
<tr><td>493</td><td>>tr|A0A0F2LZ47|A0A0F2LZ47 SPOSC GMC OxRdtase N domain-containing protein OS=Sporothrix schenckii 1099-18 OX=1397361 GN=SPSK 04896 PE=3 SV=1</td></tr>
<tr><td>494</td><td>>tr|A0A0C2ILW6|A0A0C2ILW6 9PEZI GMC OxRdtase N domain-containing protein OS=Sporothrix brasiliensis 5110 OX=1398154 GN=SPBR 06510 PE=3 SV=1</td></tr>
<tr><td>495</td><td>>KAG8628087.1 hypothetical protein KVT40 003960 Elsinoe batatas</td></tr>
<tr><td>496</td><td>>KAH8902648.1 carbohydrate-binding module family 1 protein Coniochaeta sp. PMI 546</td></tr>
<tr><td>497</td><td>>tr|A0A1J7INB3|A0A1J7INB3 9PEZI Carbohydrate-binding module family 1 protein OS=Coniochaeta ligniaria NRRL 30616 OX=1408157 GN=CONLIGDRAFT 681997 PE=3 SV=1</td></tr>
<tr><td>498</td><td>>KAF4556542.1 Cyclase atC-like protein Elsinoe fawcettii</td></tr>
<tr><td>499</td><td>>tr|A0A2K1R1J5|A0A2K1R1J5 9PEZI Cellobiose dehydrogenase OS=Sphaceloma murrayae OX=2082308 GN=CAC42 3480 PE=3 SV=1</td></tr>
<tr><td>500</td><td>>tr|A0A420XVV9|A0A420XVV9 9PEZI CBM1 domain-containing protein OS=Coniochaeta pulveracea OX=177199 GN=DL546 000103 PE=3 SV=1</td></tr>
<tr><td>501</td><td>>XP 038743215.1 cellobiose dehydrogenase Colletotrichum karsti</td></tr>
</table>

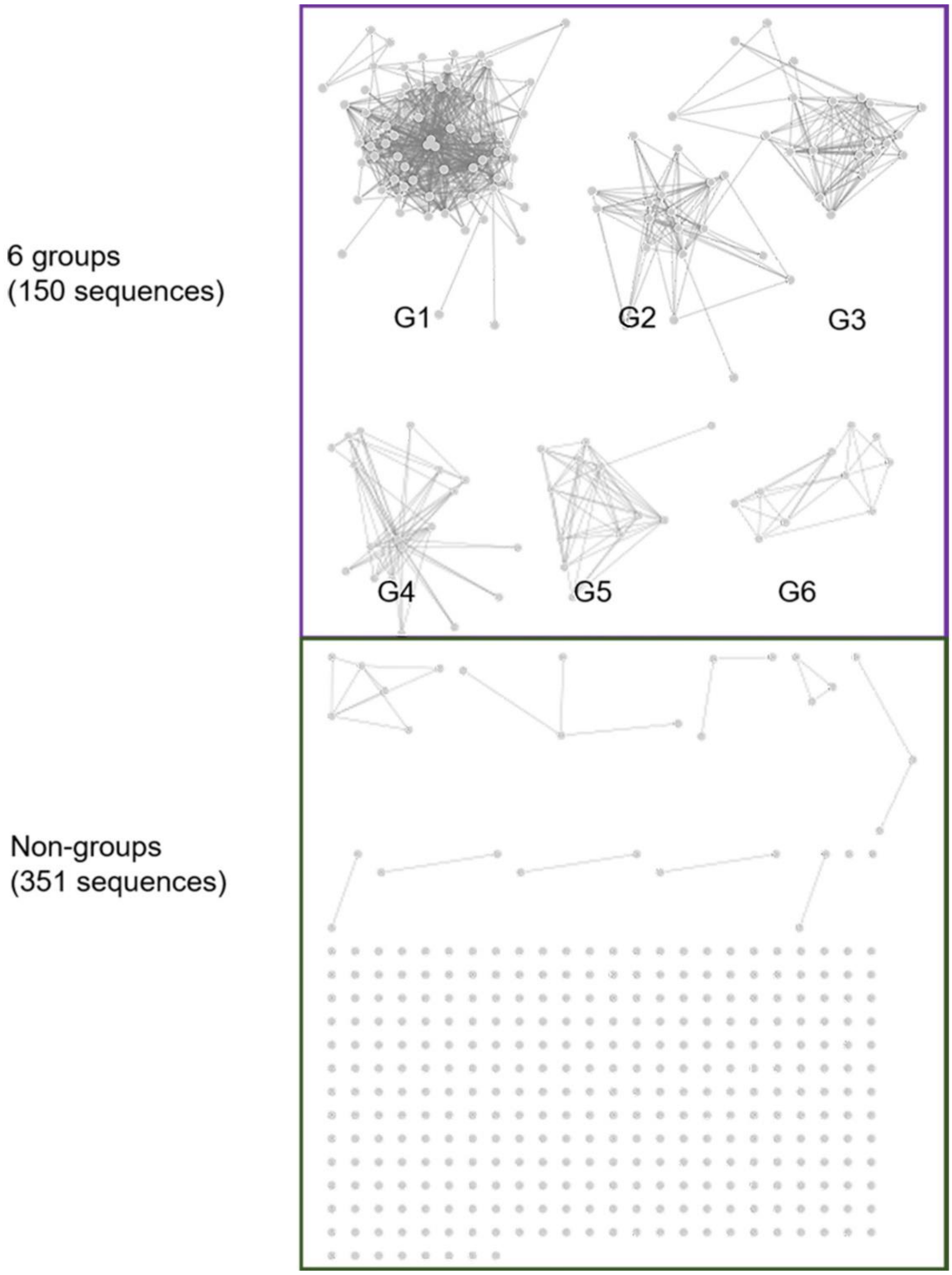

Figure S3, Sequence similarity network (SSN) of 501 Class II CDH linkers with an alignment score 1×10^{-10}. When using this strict alignment score 351/501 sequences were not assigned to one of the shown six groups. This strict cut-off was used to define meaningful groups for further analysis. The aligned sequences of each group are shown in Figure S4.

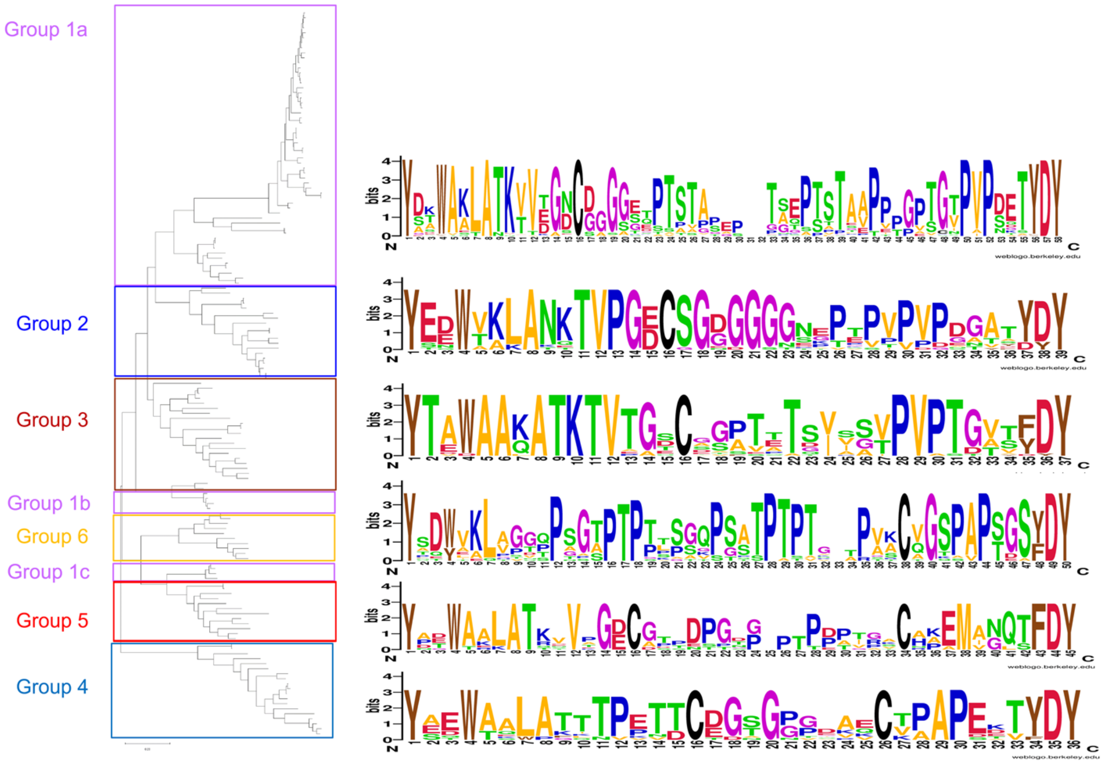

Figure S4. Phylogenetic tree of Class II cellobiose dehydrogenase linkers clustered by SSN. There are three groups separated by phylogenetic analysis that were clustered in Group 1 by SSN named Group 1a, 1b and 1c. Figure S5 shows the sequence alignment of the six linker groups classified by SSN.

Group 1 (all sequences from groups 1a, 1b, and 1c)

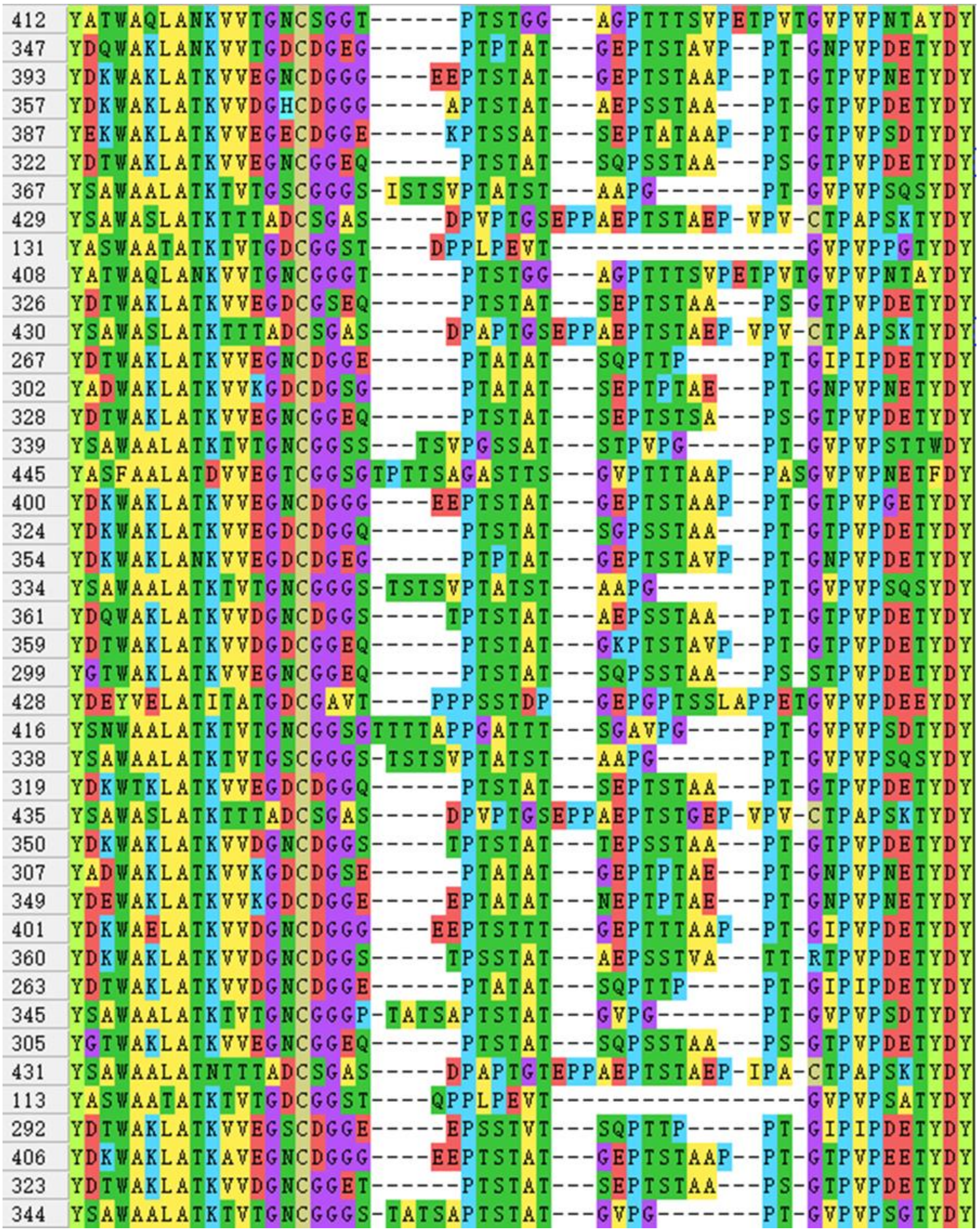

364	YDKWAKLATKVVDGNCDGGG-----QPTSTAT---AEPSSTAA---PT-GTPVPDETYDY
118	YASWAATATKEVTGDCGGST----EPPVPEVT-----------------GVPVPSGTYDY
327	YDTWAKLATKVVEGSCSGEQ------PTSTAT---SQPSSTAA---PS-GTPVPDETYDY
321	YGTWAKLATKVVEGNCGGEQ------PTSTAT---SQPTSTAA---PS-GTPVPDETYDY
306	YDDWVKLATKTVEGDCDGG-------PSPTAT---GEPTATAAP--PS-GTPVPDETYDY
226	YASWAALATKTVTGSCGGAT--------TTTT---SVPTATSVP-----GVPVPTATYDY
351	YDEWAKLATKVVDGDCDGGT-----EPTATAT---NEPSPTAE---PT-GKPVPDETYDY
405	YSAWAALATKTVSGNCGGGSTTTSAPPSSTTT---RAPTG------PT-GVPVPSGTYDY
407	YSAWAALATKTVTGNCGGGSSSTSMPPGATST---STLVPG-----PT-GVPVPSQTYDY
337	YDKWAKLATKVVDGNCDGGG-----TPTSTAT---AGPSSTAA---PT-GTPVPDETYDY
348	YDKWAKLATKVVDGNCDGGG-----TPTSTAT---AEPSSTAT---PT-GSPVPDETYDY
379	YEKWAKLATKVVEGECDGGG-----KPTSTAT---SEPTVTAAP--PT-GSPVPSETYDY
297	YDTWAKLATKVVEGDCGSGQ------PTSTAT---SQPTSTAA---PS-GTPVPDETYDY
122	YASWAATATKTVTGDCGGST----EPPVPEVT-----------------GVPVPSETYDY
398	YDKWAELATKVVDGNCDGSG----EEPTSTAT---GEPTTTAAP--PT-GIPVPDDTYDY
325	YDTWAKLATKVVEGDCGSGQ------PTSTAT---SEPTSTAA---PS-GTPVPDETYDY
358	YDKWAKLATKVVDGNCDGGS-----TPTSTAT---AEPSSTAA---PT-GTPVPDETYDY
362	YDKWAKLATKIVDGNCDGGS-----TPTSTAT---AEPSSTAA---PT-GTPVPNETYDY
363	YDTWAKLATKVVDGDCGGEQ------PTSTAT---GKPTSTAAP--PT-GTPVPDETYDY
409	YSAWAALATKTVTGNCGGGSSSTLVPPGATST---STPVPG-----PT-GVPVPSQTYDY

Group 1b

122	YASWAATATKTVTGDCGGSTEPPVPEVTGVPVPSETYDY
118	YASWAATATKEVTGDCGGSTEPPVPEVTGVPVPSGTYDY
113	YASWAATATKTVTGDCGGSTQPPLPEVTGVPVPSATYDY
131	YASWAATATKTVTGDCGGSTDPPLPEVTGVPVPPGTYDY

Group 1c

429	YSAWASLATKTTTADCSGASDPVPTGSEPPAEPTSTAEPVPVCTPAPSKTYDY
435	YSAWASLATKTTTADCSGASDPVPTGSEPPAEPTSTGEPVPVCTPAPSKTYDY
430	YSAWASLATKTTTADCSGASDPAPTGSEPPAEPTSTAEPVPVCTPAPSKTYDY
431	YSAWAALATNTTTADCSGASDPAPTGTEPPAEPTSTAEPIPACTPAPSKTYDY

According to phylogenetic analysis (Figure S4) Group 1 is divided into three branches, The majority being in Group 1a, members of Group 1b are: 113, 118, 122, 131, members of Group 1c are: 429, 430, 431, 435.

Group 2

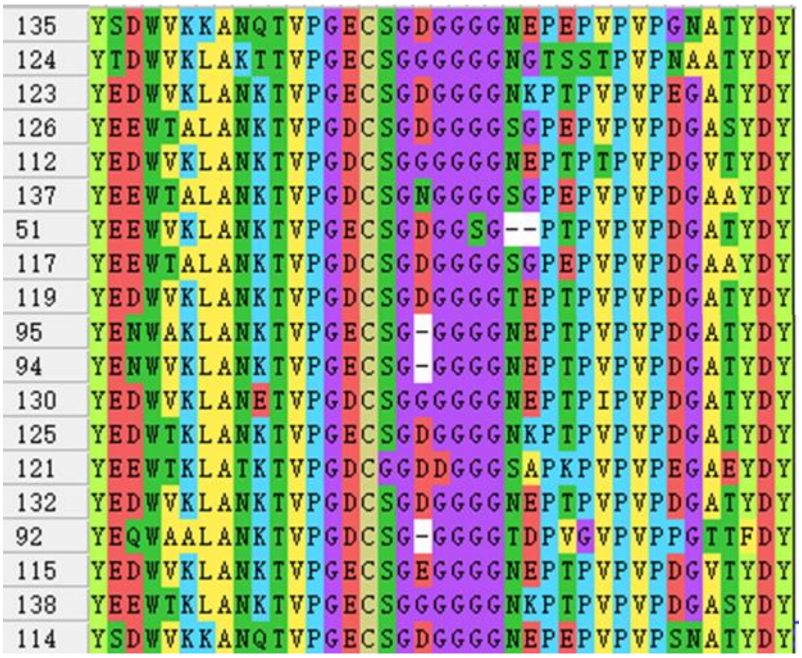
135 YSDWVKKANQTVPGECSGDGGGGNEPEPVPVPGNATYDY
124 YTDWVKLAKTTVPGECSGGGGGGNGTSSTPVPNAATYDY
123 YEDWVKLANKTVPGECSGDGGGGNKPTPVPVPEGATYDY
126 YEEWTALANKTVPGDCSGDGGGGSGPEPVPVPDGASYDY
112 YEDWVKLANKTVPGDCSGGGGGGNEPTPTPVPDGVTYDY
137 YEEWTALANKTVPGDCSGNGGGGSGPEPVPVPDGAAYDY
51 YEEWVKLANKTVPGECSGDGGSG--PTPVPVPDGATYDY
117 YEEWTALANKTVPGDCSGDGGGGSGPEPVPVPDGAAYDY
119 YEDWVKLANKTVPGDCSGDGGGGTEPTPVPVPDGATYDY
95 YENWAKLANKTVPGECSG-GGGGNEPTPVPVPDGATYDY
94 YENWVKLANKTVPGECSG-GGGGNEPTPVPVPDGATYDY
130 YEDWVKLANETVPGDCSGGGGGGNEPTPIPVPDGATYDY
125 YEDWTKLANKTVPGECSGDGGGGNKPTPVPVPDGATYDY
121 YEEWTKLATKTVPGDCGGDDGGGSAPKPVPVPEGAEYDY
132 YEDWVKLANKTVPGDCSGDGGGGNEPTPVPVPDGATYDY
92 YEQWAALANKTVPGDCSG-GGGGTDPVGVPVPPGTTFDY
115 YEDWVKLANKTVPGECSGEGGGGNEPTPVPVPDGVTYDY
138 YEEWTKLANKTVPGECSGGGGGGNKPTPVPVPDGASYDY
114 YSDWVKKANQTVPGECSGDGGGGNEPEPVPVPSNATYDY

Group 3

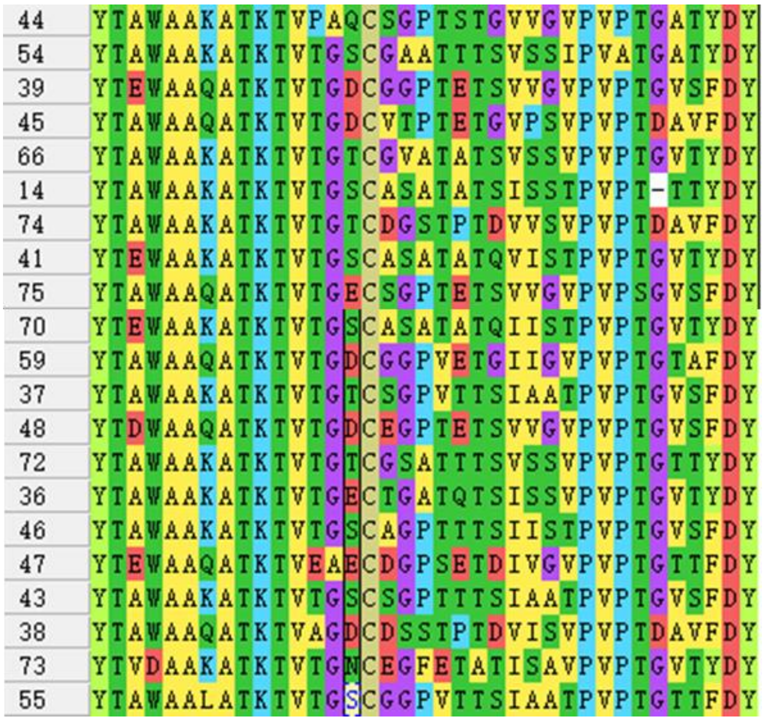

Group 4

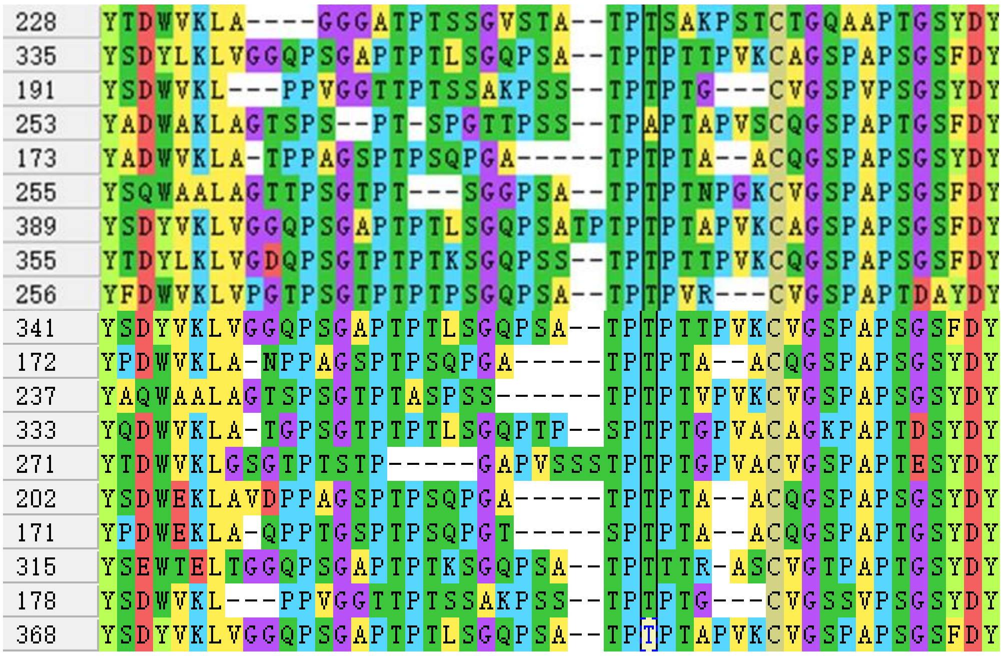

Group 5

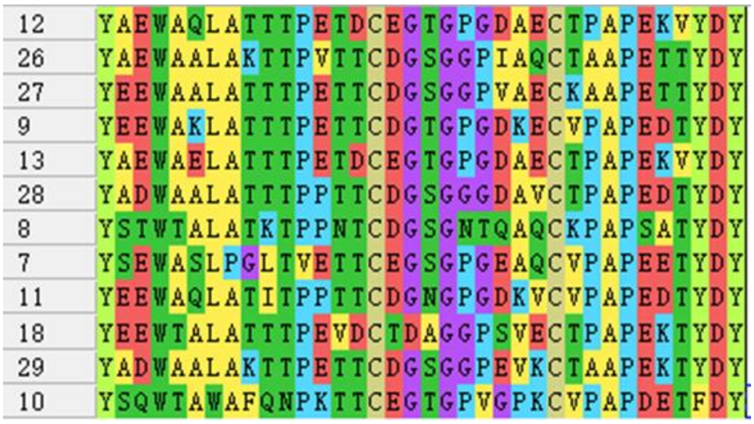

Group 6

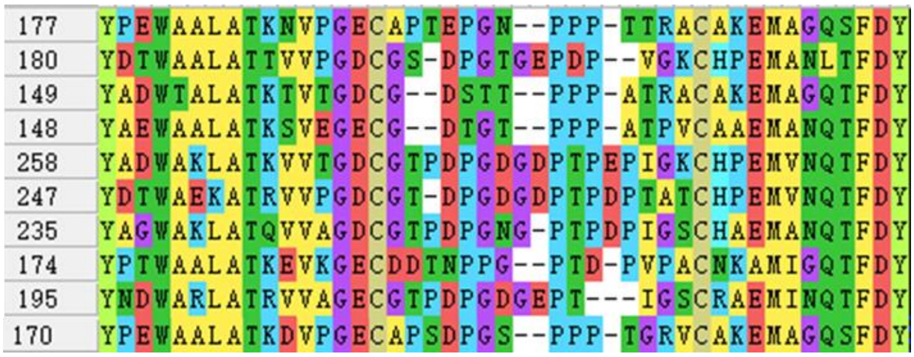

Figure S5. Multiple sequence alignment of 150 Class II CDH linker sequences with an SSN alignment score 1×10^{-10} separated into six groups.

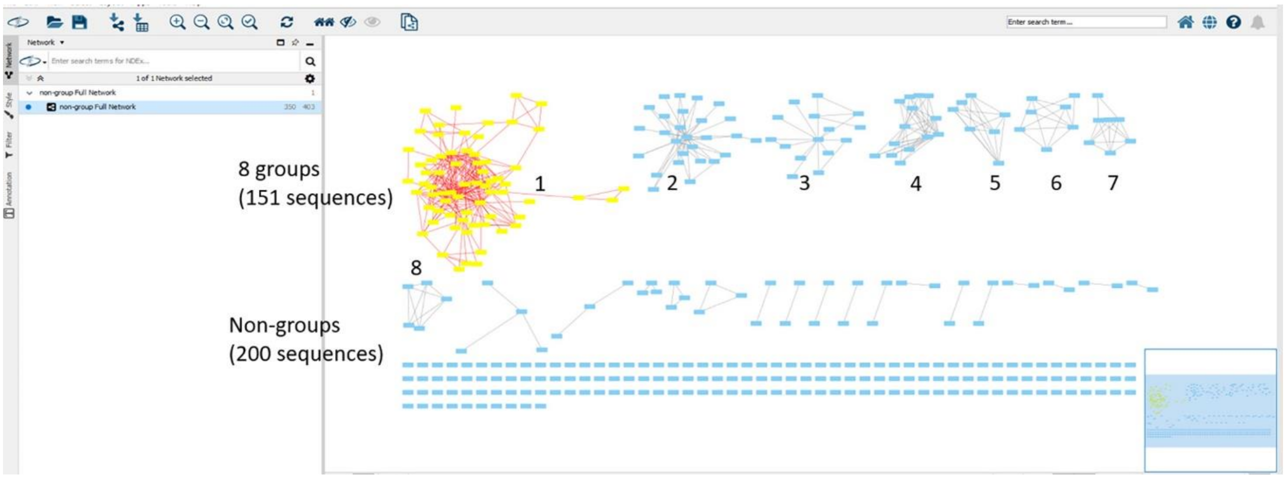

Figure S6. SSN of the remaining 351 Class II CDH linker sequences with an alignment score of 1×10^{-5} (the lowest possible cut-off). The aligned sequences of each group are shown in Figure S8.

Group R01

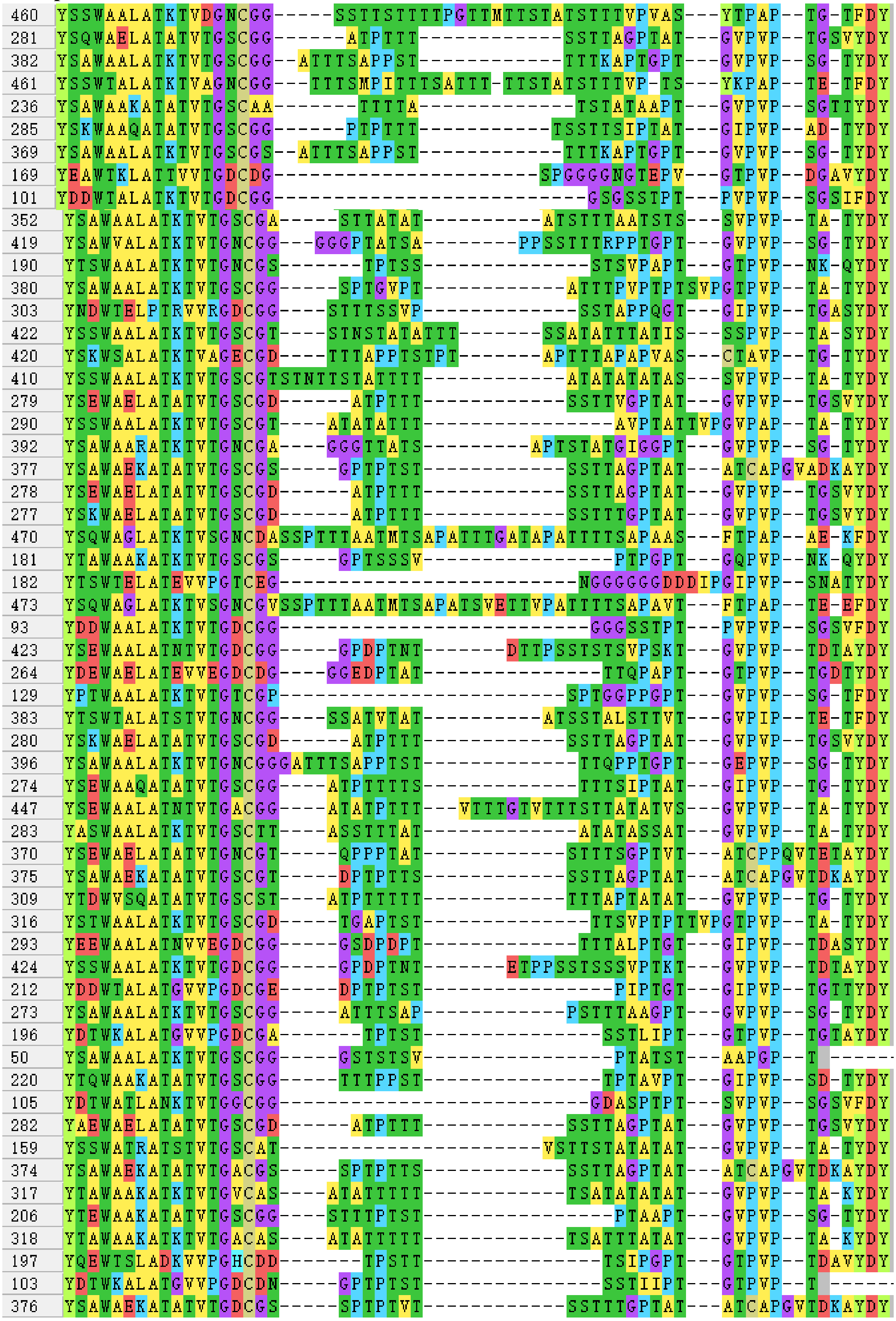

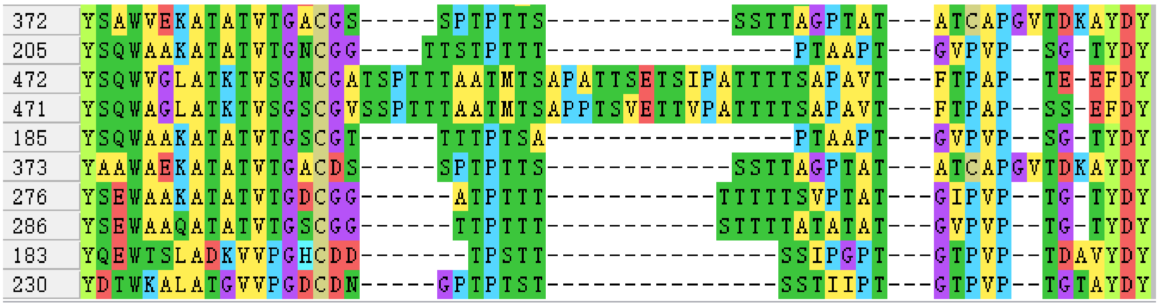

372 YSAWVEKATATVTGACGS-----SPTPTTS-------------SSTTAGPTAT---ATCAPGVTDKAYDY
205 YSQWAAKATATVTGNCGG----TTSTPTTT-----------------PTAAPT---GVPVP--SG-TYDY
472 YSQWVGLATKTVSGNCGATSPTTTAATMTSAPATTSETSIPATTTTSAPAVT---FTPAP--TE-EFDY
471 YSQWAGLATKTVSGSCGVSSPTTTAATMTSAPPTSVETTVPATTTTSAPAVT---FTPAP--SS-EFDY
185 YSQWAAKATATVTGSCGT-----TTTPTSA-----------------PTAAPT---GVPVP--SG-TYDY
373 YAAWAEKATATVTGACDS-----SPTPTTS-------------SSTTAGPTAT---ATCAPGVTDKAYDY
276 YSEWAAKATATVTGDCGG------ATPTTT------------TTTTTSVPTAT---GIPVP--TG-TYDY
286 YSEWAAQATATVTGSCGG------TTPTTT------------STTTTATATAT---GVPVP--TG-TYDY
183 YQEWTSLADKVVPGHCDD-------TPSTT----------------SSIPGPT---GTPVP--TDAVYDY
230 YDTWKALATGVVPGDCDN-----GPTPTST----------------SSTIIPT---GTPVP--TGTAYDY

Group R02

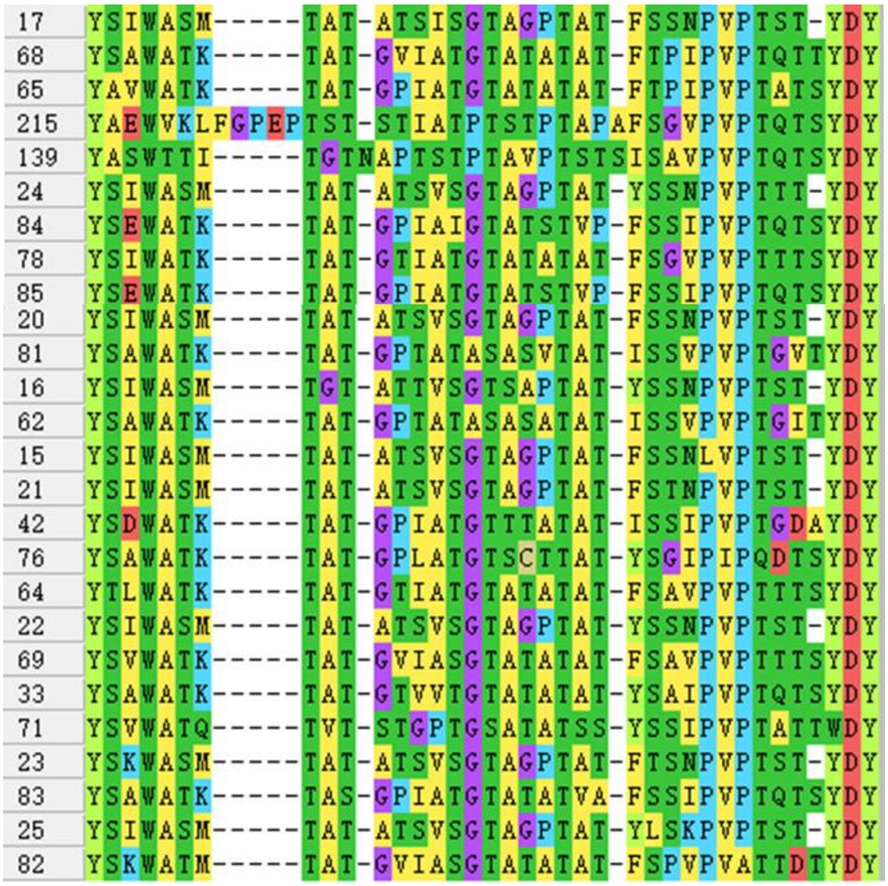

17 YSIWASM------TAT-ATSISGTAGPTAT-FSSNPVPTST-YDY
68 YSAWATK------TAT-GVIATGTATATAT-FTPIPVPTQTTYDY
65 YAVWATK------TAT-GPIATGTATATAT-FTPIPVPTATSYDY
215 YAEWVKLFGPEPTST-STIATPTSTPTAPAFSGVPVPTQTSYDY
139 YASWTTI------TGTNAPTSTPTAVPTSTSISAVPVPTQTSYDY
24 YSIWASM------TAT-ATSVSGTAGPTAT-YSSNPVPTTT-YDY
84 YSEWATK------TAT-GPIAIGTATSTVP-FSSIPVPTQTSYDY
78 YSIWATK------TAT-GTIATGTATATAT-FSGVPVPTTTSYDY
85 YSEWATK------TAT-GPIATGTATSTVP-FSSIPVPTQTSYDY
20 YSIWASM------TAT-ATSVSGTAGPTAT-FSSNPVPTST-YDY
81 YSAWATK------TAT-GPTATASASVTAT-ISSVPVPTGVTYDY
16 YSIWASM------TGT-ATTVSGTSAPTAT-YSSNPVPTST-YDY
62 YSAWATK------TAT-GPTATASASATAT-ISSVPVPTGITYDY
15 YSIWASM------TAT-ATSVSGTAGPTAT-FSSNLVPTST-YDY
21 YSIWASM------TAT-ATSVSGTAGPTAT-FSTNPVPTST-YDY
42 YSDWATK------TAT-GPIATGTTTATAT-ISSIPVPTGDAYDY
76 YSAWATK------TAT-GPLATGTSCTTAT-YSGIPIPQDTSYDY
64 YTLWATK------TAT-GTIATGTATATAT-FSAVPVPTTTSYDY
22 YSIWASM------TAT-ATSVSGTAGPTAT-YSSNPVPTST-YDY
69 YSVWATK------TAT-GVIASGTATATAT-FSAVPVPTTTSYDY
33 YSAWATK------TAT-GTVVTGTATATAT-YSAIPVPTQTSYDY
71 YSVWATQ------TVT-STGPTGSATATSS-YSSIPVPTATTWDY
23 YSKWASM------TAT-ATSVSGTAGPTAT-FTSNPVPTST-YDY
83 YSAWATK------TAS-GPIATGTATATVA-FSSIPVPTQTSYDY
25 YSIWASM------TAT-ATSVSGTAGPTAT-YLSKPVPTST-YDY
82 YSKWATM------TAT-GVIASGTATATAT-FSPVPVATTDTYDY

Group R03

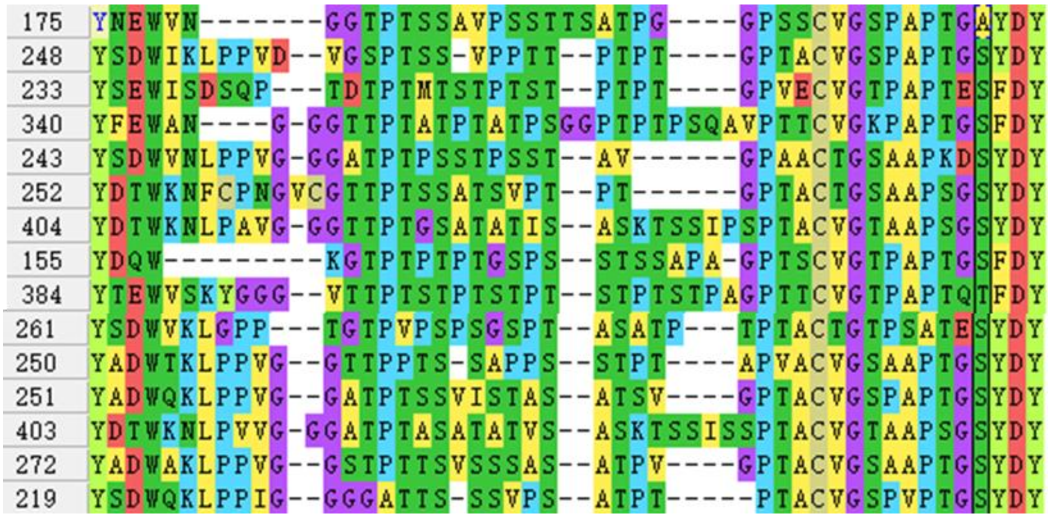

Group R04

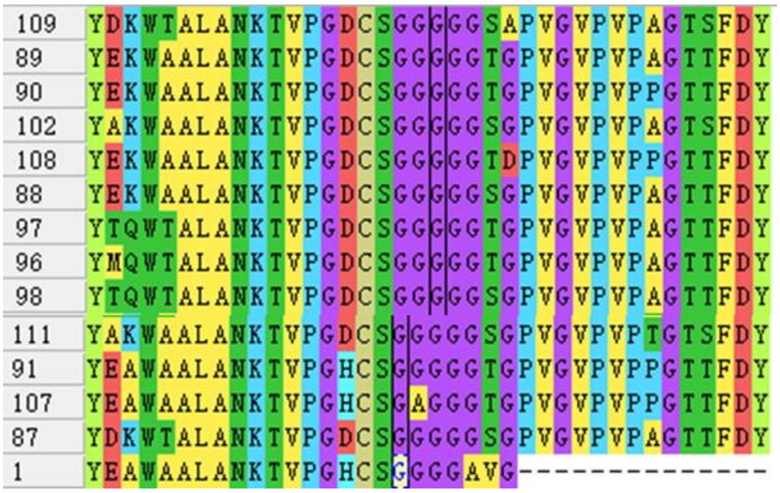

Group R05

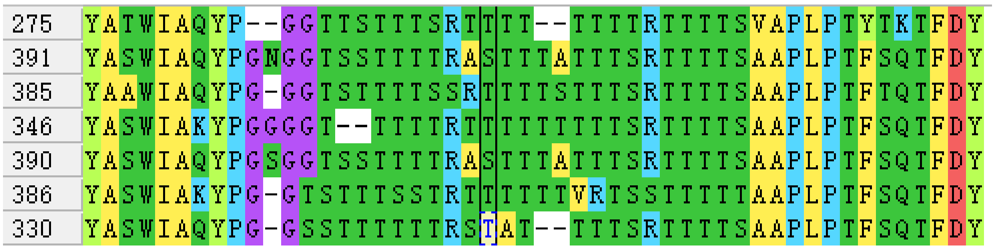

Group R06

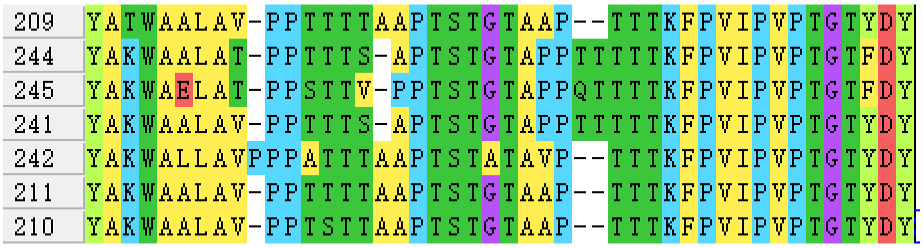

Group R07

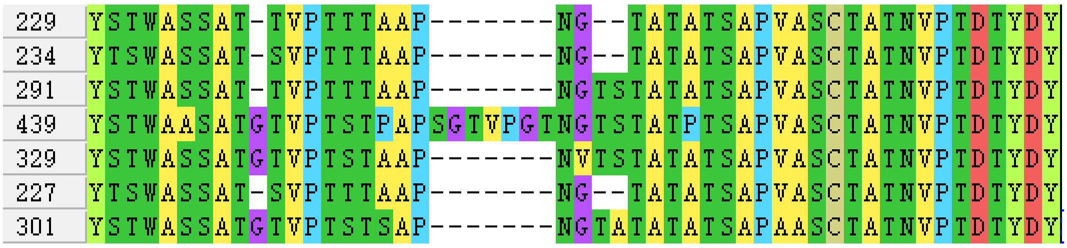

Group R08

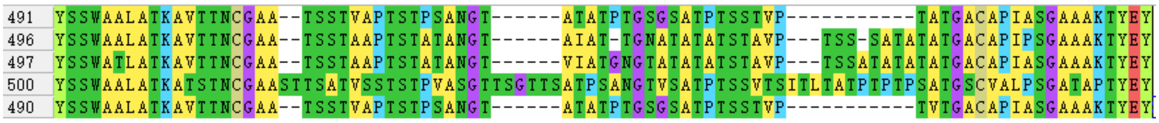

Figure S7. Sequence alignment of 151 of the remaining 351 Class II CDH linker sequences with an SSN alignment score of 1×10^{-5} into eight groups.

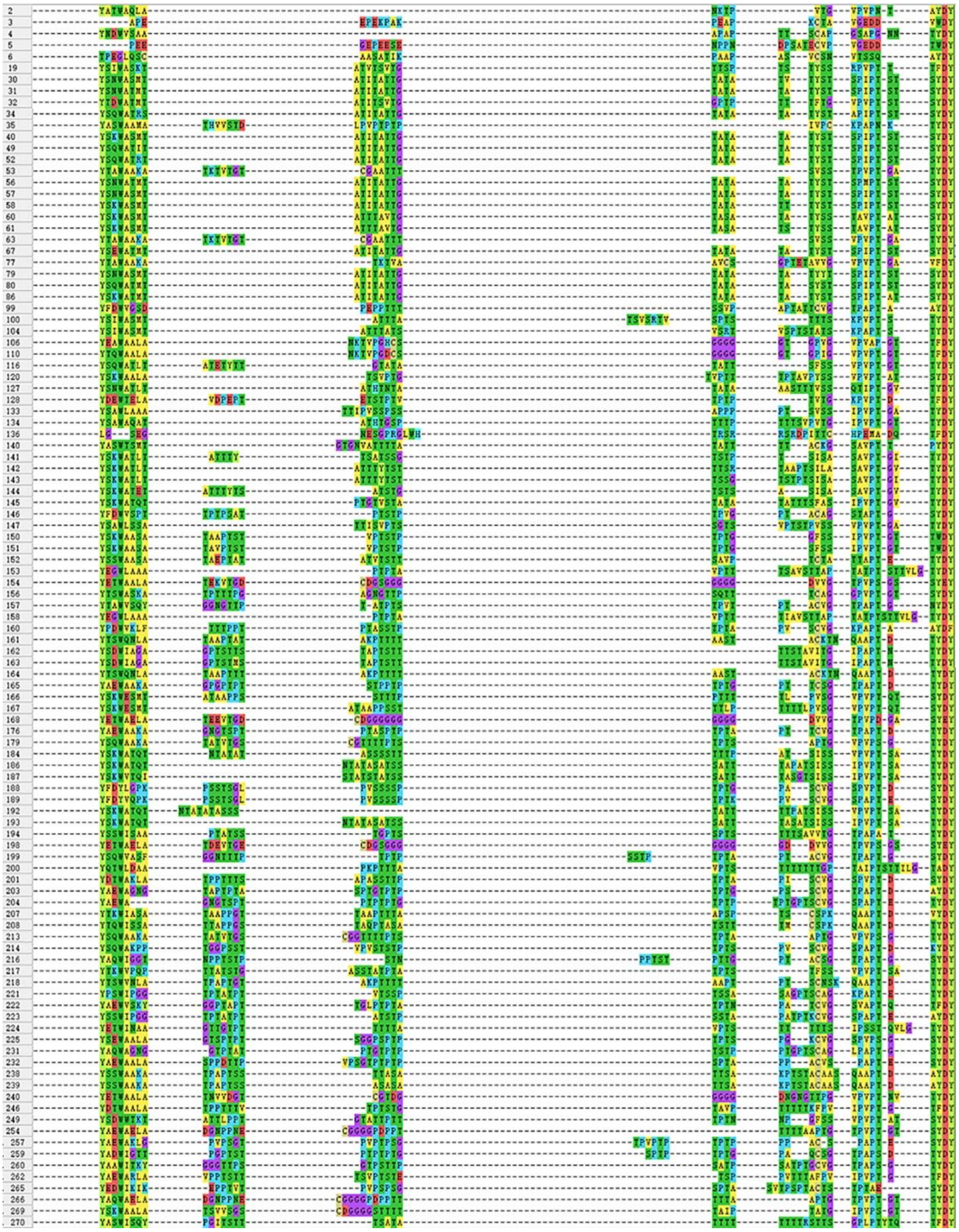

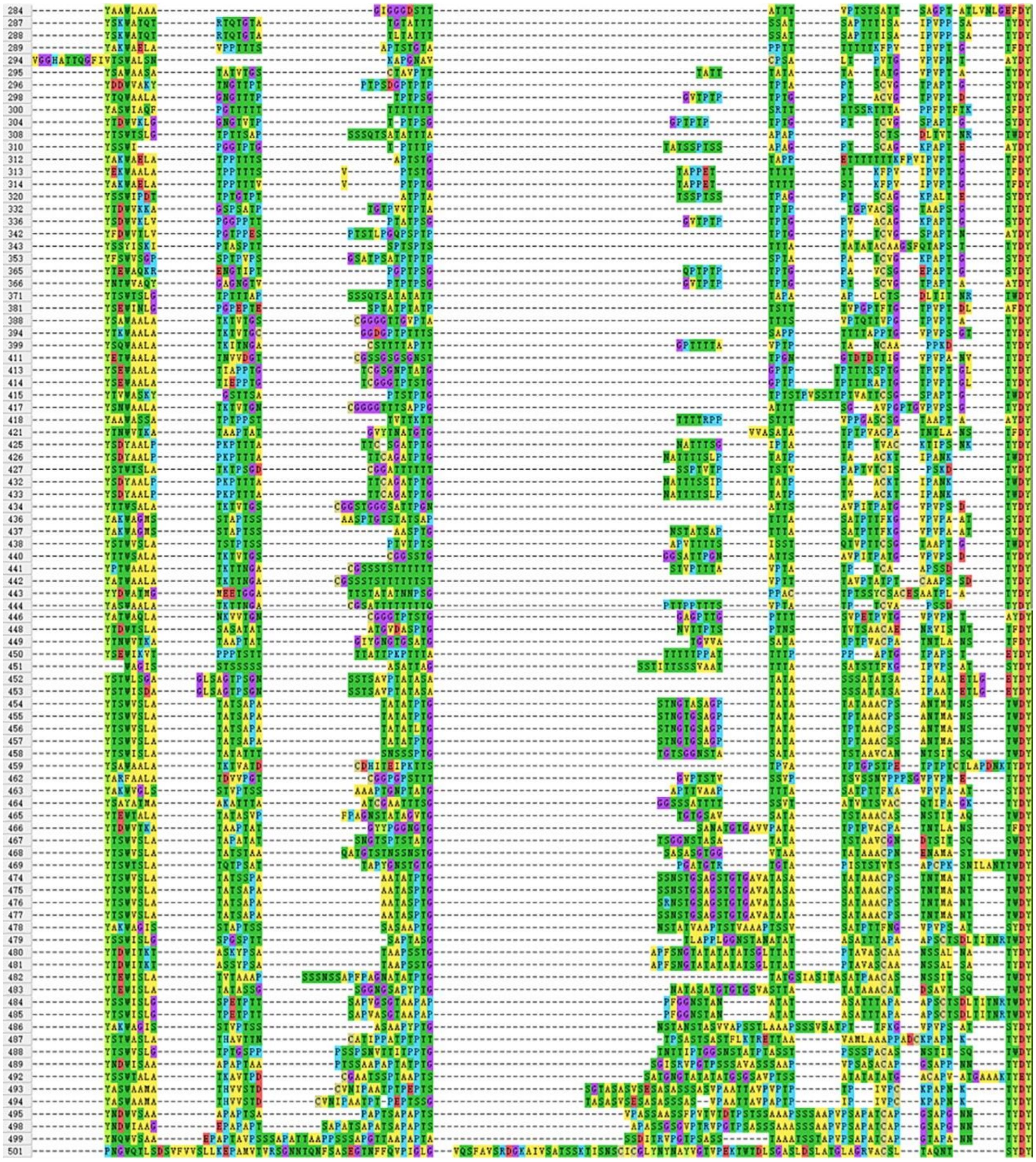

Figure S8. Ungrouped sequences of the 200 remaining ascomycete Class II CDH linkers.

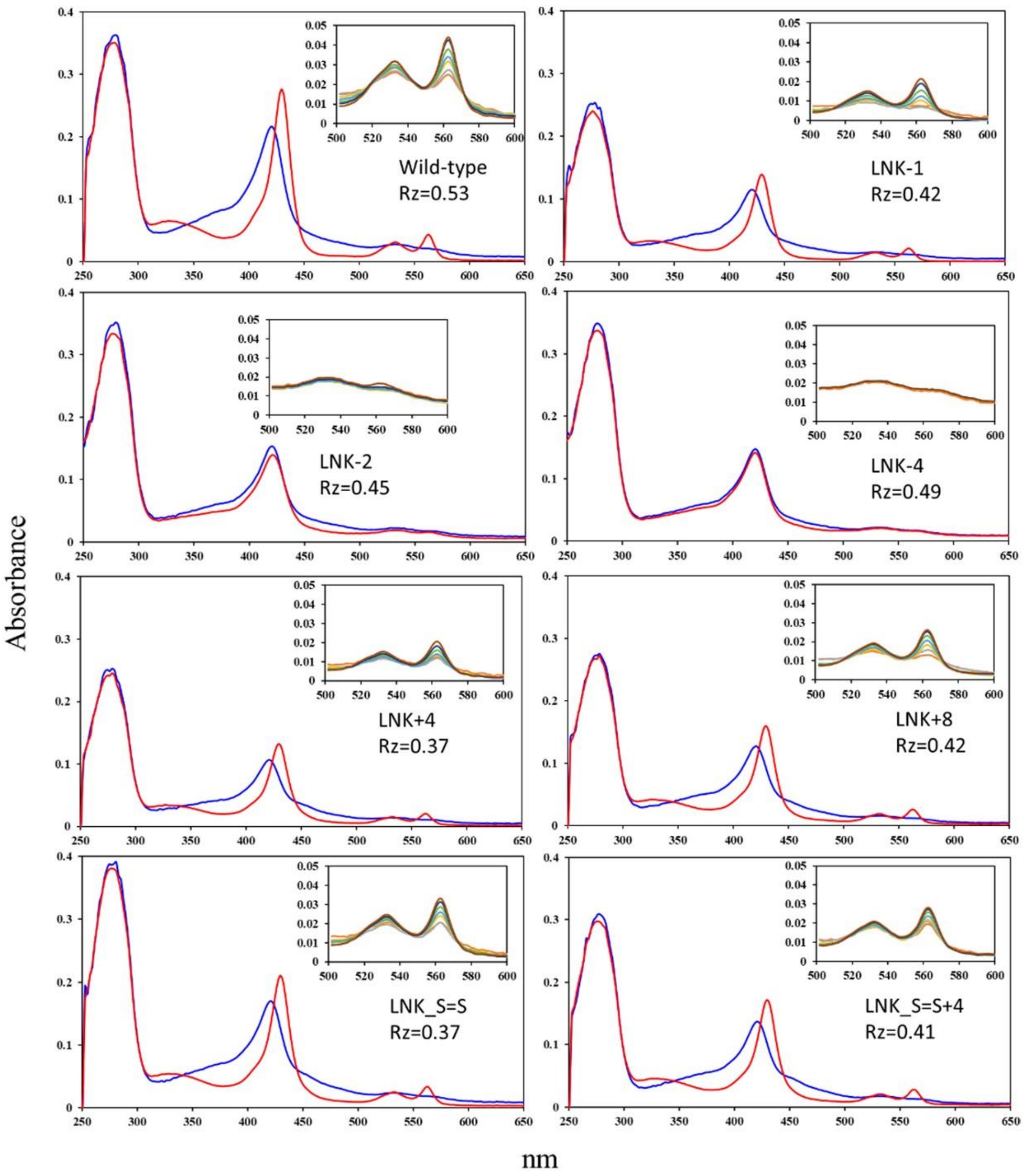

Figure S9. Spectra of purified wild-type and linker variants of CDH in both their oxidized and reduced forms (cellobiose addition) at different time points. The data were taken from the recordings of the diode array during stopped-flow measurements.

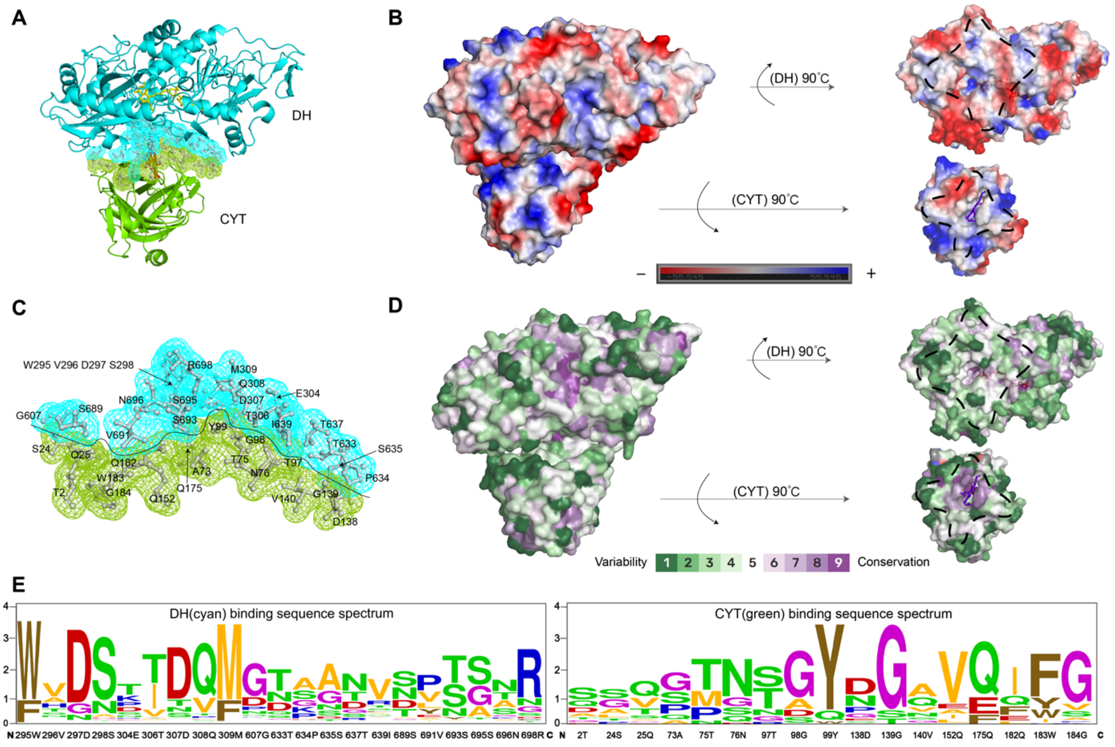

Figure S10. Domain interface analysis of CDH. A: *Nc*CDHIIA structure in close-state conformation, protein in carton, interfaces in spheres; B: Surface potential diagram, the dotted line covers the domain interfaces; C: Interacting amino acids in interfaces, DH interface in cyan, CYT interface in green; D: Conservation diagram of CDH, the dotted line covers the domain interfaces; E: Binding sequence spectra of domain interfaces.

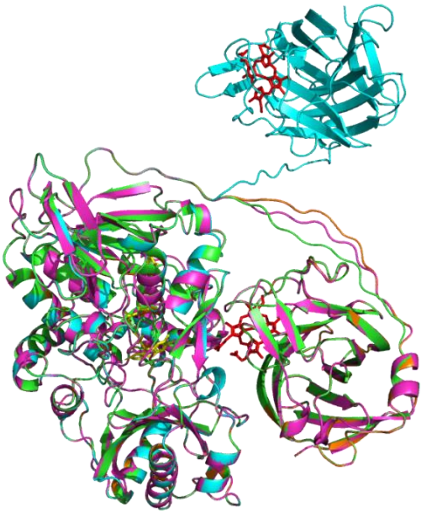

Figure S11. The comparison of the modeled structures of wild-type *Nc*CDHIIA, LNK-1, LNK-2, and LNK-4. SWISS-MODEL was used to build the variants' structures. Wild-type *Nc*CDHIIA: orange; LNK-1: magenta; LNK-2: green: LNK-4: cyan; FAD: yellow; heme: red.

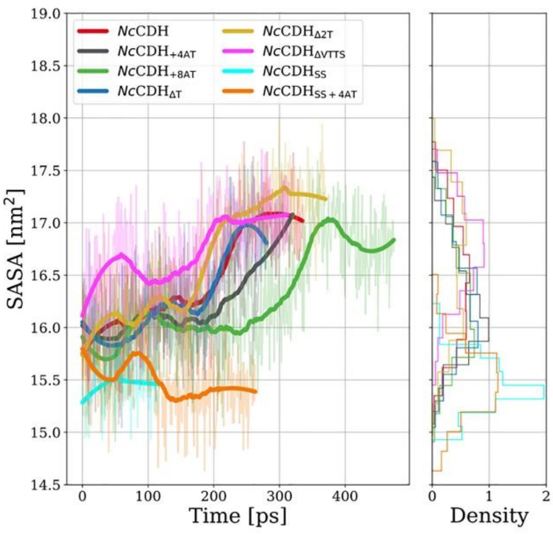

Figure S12. Solvent accessible surface area (SASA) of the C-terminal wild-type and variant linker parts. The solvent accessible surface area increases with an extended distance between the domains.

Table S3. Simulation time of the steered molecular dynamics simulations. (shown in Figure S14)

variant	simulation time [ps]
Wild-type	336
LNK+4	321
LNK+8	475
LNK–1	281
LNK–2	371
LNK–4	313
LNK_S=S	118
LNK_S=S+4	264

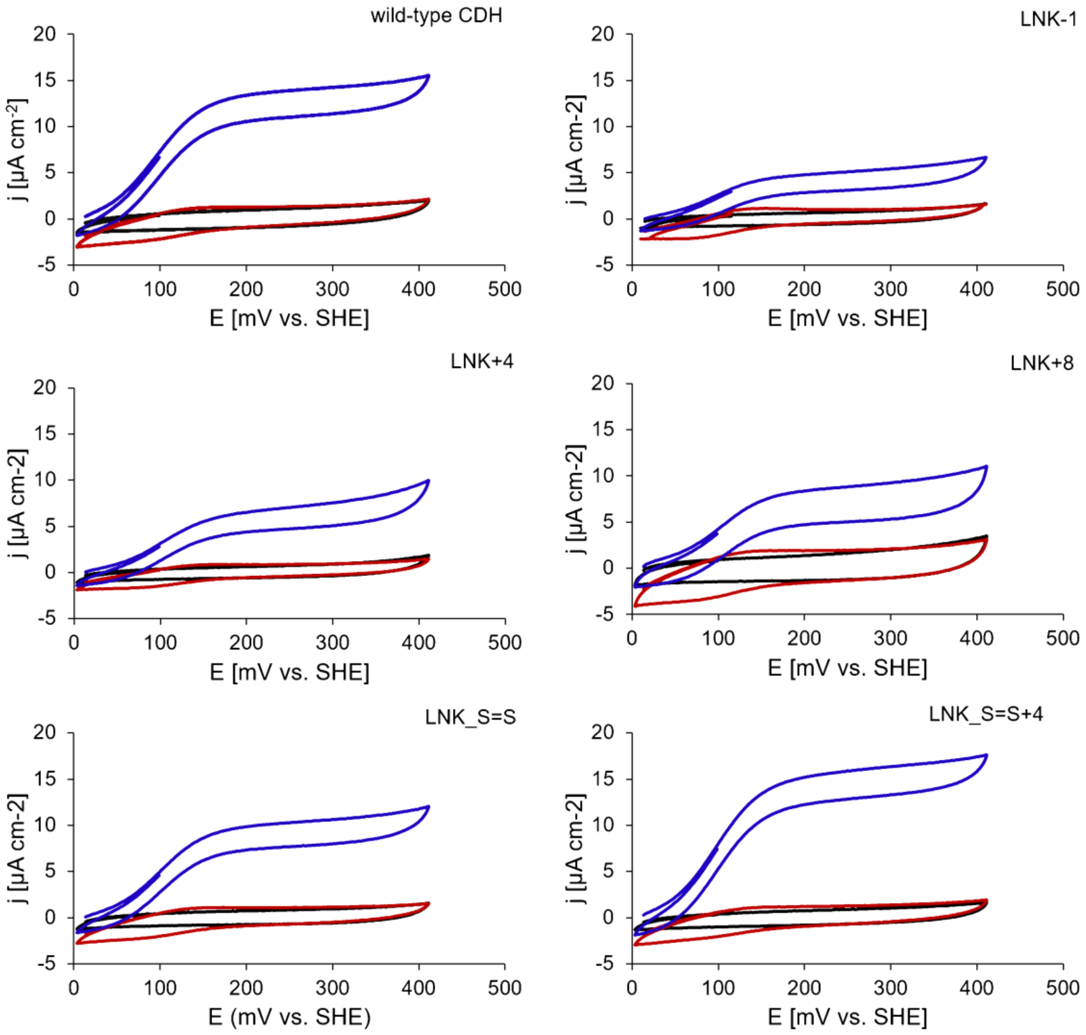

Figure S13. Cyclic voltammograms of wild-type and linker variants of CDH at 25 mV s^{-1} on thiol-glycerol modified gold electrodes. Before (red curve) and after addition of cellobiose (final concentration = 10 mM, blue curve).

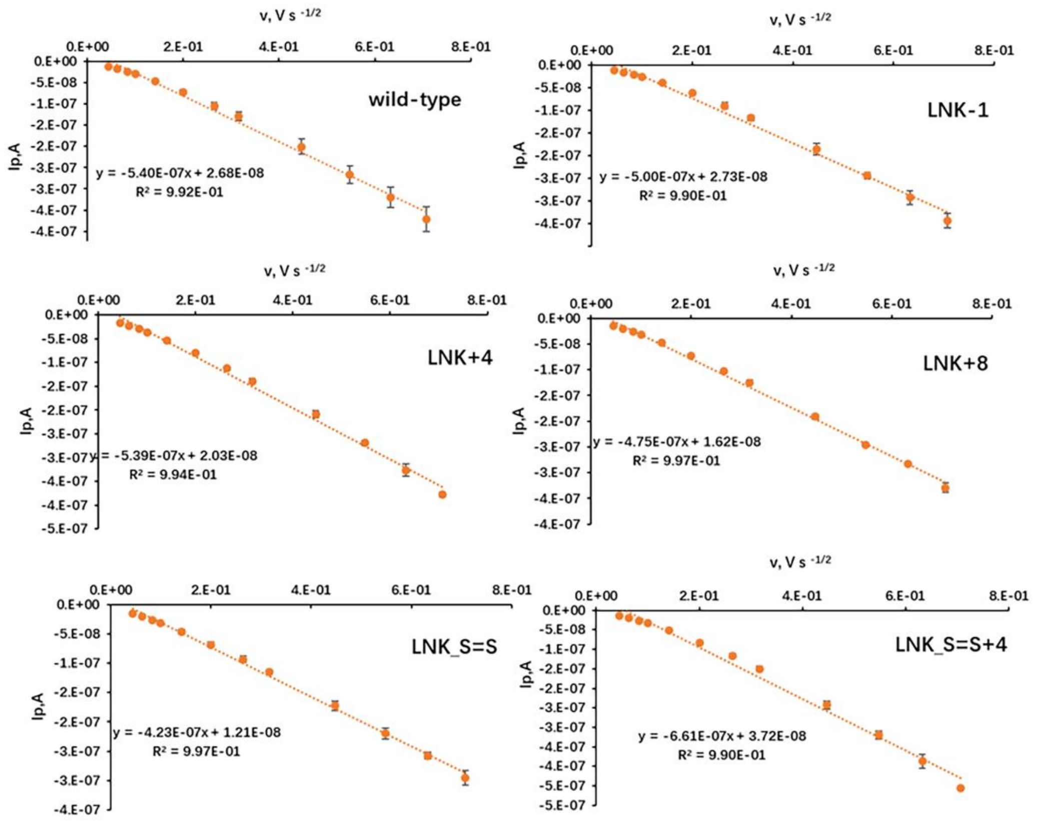

Figure S14. Determination of the state (adsorbed or diffusing) of the CDH linker variants on 1-thioglycerol-modified gold electrodes. Mean values and standard deviations are calculated from 3 independent electrodes.

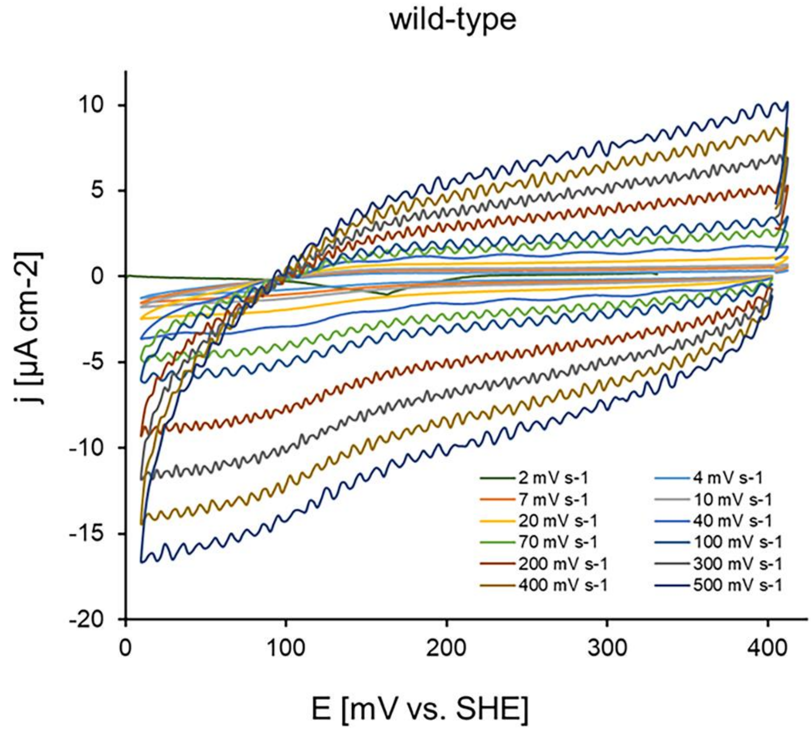

Figure S15. Cyclic voltammogram of wild-type *Nc*CDHIIA. Recorded on a 1-thioglycerol-modified gold electrode with scan rates ranging from 2 to 500 mV s^{-1}.

Table S4. Primers used for the construction of CDH linker variants.

Primer ID	Primer Sequence 5’-3’
LNK–1 - fw	AGTATTGCCGCCACTCCTGTTC
LNK–1 - rv	CGGCAATACTCGTGACTGGACCACTGCAAGTACC
LNK–2 - fw	AGTATTGCCGCCACTCCTGTTC
LNK–2 - rv	TGGCGGCAATACTGACTGGACCACTGCAAGTACCG
LNK–4 - fw	ATTGCCGCCACTCCTGTTCCCAC
LNK–4 - rv	GAGTGGCGGCAATTGGACCACTGCAAGTACCGGT
LNK+4 - fw	GCTACTGCTACTACCAGTATTGCCGCCACTC
LNK+4 - rv	AGTAGCAGTAGCCGTGACTGGACCACTGCAAG
LNK+8 - fw	GCTACTGCTACTACCAGTATTGCCGCCACTCC
LNK+8 - rv	AGTAGCAGTAGCAGTAGCAGTAGCCGTGACTGGACC
LNK_S=S - fw	CAGTCACGACCAGTTGTGCCGCCACTCCTGTTC
LNK_S=S - rv	ACTGGTCGTGACTGGACCAC
LNK_S=S + 4 AT - fw	GCTACTGCTACTACCAGTTGTGCCGCCACTCC
LNK_S=S + 4 AT - rv	AGTAGCAGTAGCCGTGACTGGACCACTGCAAG
